# Synthesis, properties, and material hybridization of bare aromatic polymers enabled by dendrimer support

**DOI:** 10.1038/s41467-022-33100-7

**Published:** 2022-09-16

**Authors:** Shusei Fujiki, Kazuma Amaike, Akiko Yagi, Kenichiro Itami

**Affiliations:** 1 Department of Chemistry, Graduate School of Science, Nagoya University, Chikusa, Nagoya, 464-8602 Japan; 2 Institute of Transformative Bio-Molecules (WPI-ITbM), Nagoya University, Chikusa, Nagoya, 464-8602 Japan

**Keywords:** Organic chemistry, Polymer chemistry

## Abstract

Aromatic polymers are the first-choice platform for current organic materials due to their distinct optical, electronic, and mechanical properties as well as their biocompatibility. However, bare aromatic polymer backbones tend to strongly aggregate, rendering them essentially insoluble in organic solvent. While the typical solution is to install many solubilizing substituents on the backbones, this often provokes undesired property changes. Herein, we report the synthesis of bare aromatic polymers enabled by a dendrimer support. An initiator arene containing a diterpenoid-based dendrimer undergoes Pd-catalyzed polymerization with monomers bearing no solubilizing substituents to furnish bare aromatic polymers such as polythiophenes and poly(*para*-phenylene)s. The high solubility of dendrimer-ligated polymers allows not only the unveiling of the properties of unsubstituted π-conjugated backbone, but also mild release of dendrimer-free aromatic polymers and even transfer of aromatic polymers to other materials, such as silica gel and protein, which may accelerate the creation of hybrid materials nowadays challenging to access.

## Abstract (web summary)

Unsubstituted aromatic polymers are materials with multiple potential applications, but their preparation remains challenging. Here, the authors report a dendrimer-enabled synthesis of soluble bare aromatic polymers and explore their properties; these compounds can be further transformed into other materials.

## Introduction

**Fig. 1:**
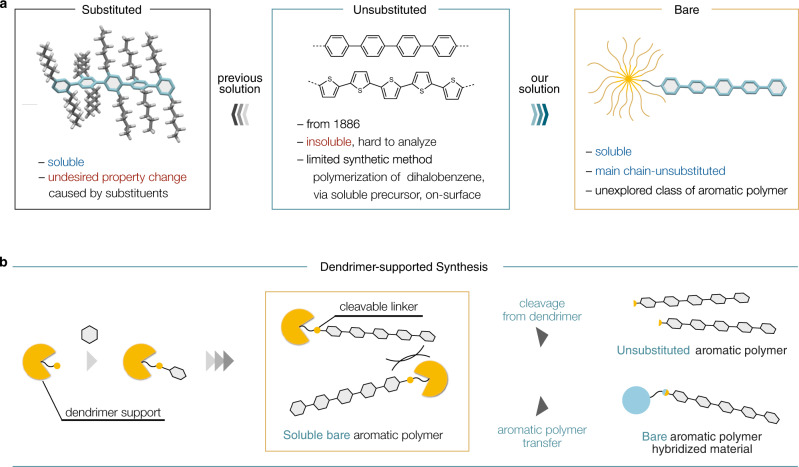
Synthesis of bare aromatic polymers. **a** Unsubstituted aromatic polymers such as poly(*para*-phenylene) (PPP) and polythiophene (PT) (middle); main chain-substituted aromatic polymers (left); main chain-unsubstituted bare aromatic polymers (right). **b** Dendrimer-supported synthesis and transformations of bare aromatic polymers.

Aromatic polymers are one of the central and essential materials supporting our daily life and serve as the first-choice platform for next-generation materials due to their distinct optical, electronic, and mechanical properties as well as their biocompatibility. In particular, poly(*para*-phenylene)s (PPPs) and polythiophenes (PTs) have received a tremendous amount of interest due to their high performance as conducting and luminescent materials (Fig. 1a)^1^. As their properties critically depend on structural factors, chemists have devoted decades of efforts in the precise synthesis of aromatic polymers^2,3^. The first syntheses of PPPs and PTs date back to 1886^4^ and 1941^5^, respectively, and since then these attractive polymers have been synthesized by the classical Wurtz–Fittig^6^ coupling and/or electrochemical polymerization^7^. With the advent of transition metal-mediated coupling reactions, the use of Kumada–Tamao coupling^8^ and Yamamoto coupling^9^ of difunctionalized arenes has become the standard in the field. However, due to strong intermolecular *π*−*π* interactions, the parent PPPs and PTs (without any substituents) are insoluble to solvents; these molecules are already barely soluble at six monomer units (e.g., the solubility of terphenylene; 8.5 g/L vs sexiphenylene; < 0.01 g/L)^10^. Because of this solubility problem, the direct synthesis, property analysis, and material manipulation/transfer of long bare aromatic polymers have remained elusive. While several alternative methods using well-designed soluble precursors^11–16^ or surface-assisted synthesis^17–22^ have been developed for PPP synthesis, bare PPPs can only be obtained as insoluble aggregates which are hard to use for any analysis or application.

As a means of circumventing the solubility problem in aromatic polymers, the concept of introducing solubilizing side chains onto the polymer backbones emerged in the late 1980s (Fig. 1a)^23–26^. Long side chains can confer the desired solubility to the aromatic polymers resulting in good processability and easy handling. Indeed, most of the aromatic polymers reported today are these side chain-containing polymers. On the other hand, the long side chains cause undesired property changes, including the diminution of thermal stability and carrier mobility as well as the blue-shift of emission bands derived from the distortion of the *π*-conjugated backbone^27,28^. From the viewpoints of improving performance and unveiling these lost properties, a groundbreaking strategy for the synthesis of soluble but main chain-unsubstituted bare aromatic polymers is highly sought-after. This will clearly lead to an alternative stream of escape from main chain modification in *π*-conjugated polymer science^29^.

Herein, we report a general synthetic method for bare aromatic polymers, which are otherwise quite difficult to synthesize because of the solubility problem (Fig. 1b). We envisioned that even just one substituent at the terminal end can solubilize aromatic polymers if the substituent is giant enough to prevent aggregation, while maintaining bare aromatic polymer backbones (Fig. 1a). The key is to utilize a dendrimer molecule as the solitary solubilizing group for bare aromatic polymers. The dendrimer molecule enables the catalytic polymerization of unsubstituted arene monomers in solution phase. Notably, dendrimer-ligated bare aromatic polymers are highly soluble in common organic solvents such as chloroform, tetrahydrofuran (THF) and hexane, which enables the measurement of physical properties as well as the hybridization of aromatic polymers with other functional materials such as protein and silica gel. Furthermore, at-will release of aromatic polymers from the dendrimer has been achieved and successfully afforded truly unsubstituted aromatic polymers.

## Results and discussion

### Design and synthesis of dendrimer unit

Dendrimers are known as highly branched, multivalent and mono-dispersed molecules with synthetic elasticity^30^. They have a large number of end groups at its periphery, giving various functions to themselves. They have been applied to various fields including biomedical research^31^, metal encapsulation^32^ and liquid crystals^33^. As a pioneering work to utilize dendrimers for solubilizing insoluble molecules, Fréchet reported the on-dendrimer synthesis of oligothiophene (up to *n* = 9) in 1999^34^. We assumed that aromatic polymers ligated to a giant dendrimer can gain high solubility owing to the steric hindrance preventing the aggregation. Based on this concept, we designed a dendrimer **5** as a solubilizing group for aromatic polymers. The three-dimensional tetraarylmethane motif was employed as the core to create a spherically expanded dendrimer. Branched alkyl chains, which generally afford better solubility than linear ones^35,36^, were installed at the periphery of the dendrimer to achieve high steric hindrance. The terminal aryl iodide moiety serves as the reaction site of aromatic polymer synthesis and the ester moiety provides facile cleavage of aromatic polymer from the dendrimer.

**Fig. 2:**
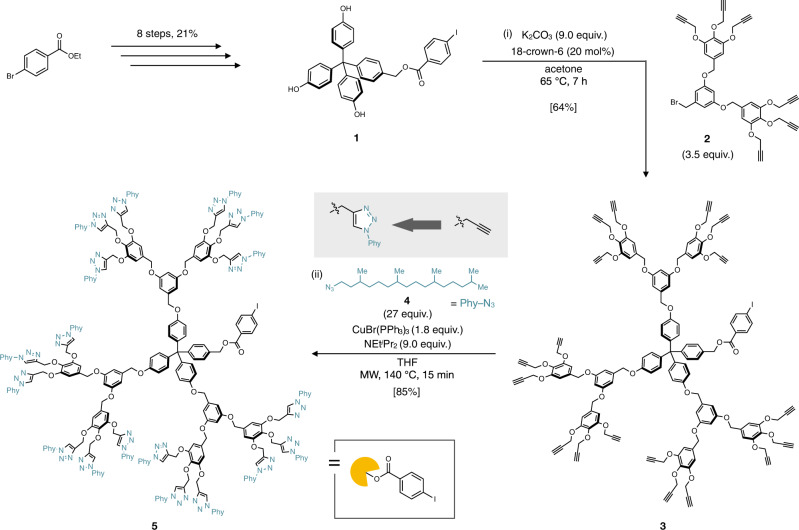
Synthesis of dendrimer support. Reagents and conditions: (i) **2** (3.5 equiv.), K_2_CO_3_ (9.0 equiv.), 18-crown-6 (20 mol%), acetone, 65 °C, 7 h; (ii) **4** (27 equiv.), CuBr(PPh_3_)_3_ (1.8 equiv.), NEt^*i*^Pr_2_ (9.0 equiv.), THF, microwave, 140 °C, 15 min. Ph phenyl, Et ethyl, ^*i*^Pr *iso*-propyl,THF tetrahydrofuran.

The designed dendrimer support **5** was synthesized as shown in Fig. 2. First, tetraarylmethane core **1** was synthesized in eight steps from commercially available ethyl 4-bromobenzoate (see the Supplementary Information (SI) for details). The thus-obtained dendrimer core **1** was coupled with second-generation dendron **2** in the presence of K_2_CO_3_ (9.0 equiv.) and 18-crown-6 (20 mol%) in acetone at 65 °C to furnish alkyne-terminated dendrimer **3** in decent yield. Finally, diterpenoid alkyl chain was installed to the dendrimer surface by highly efficient Huisgen cycloaddition of **3** with phytyl azide (**4**), in the presence of CuBr(PPh_3_)_3_ (1.8 equiv.) and NEt^*i*^Pr_2_ (9.0 equiv.) in THF at 140 °C under microwave irradiation, to furnish the desired dendrimer **5**. This dendrimer support **5**, which has 18 long branched alkyl chains on its periphery, is a sticky solid and exhibits excellent solubility in chloroform, THF and hexane but is poorly soluble in methanol and acetone.

### On-dendrimer polythiophene synthesis by catalyst-transfer Suzuki–Miyaura polymerization

The designed on-dendrimer synthesis of bare aromatic polymers can in principle be applied to any type of polymerization reactions. In this study, we focused on the use of catalyst-transfer Suzuki–Miyaura polymerization, which has been established by Yokozawa^37^ and McCullough^38^. In this reaction, the oxidative addition of the Pd complex works as the initiator of polymerization. After the transmetalation and reductive elimination with monomer, intramolecular oxidative addition proceeds owing to the interaction between palladium complex and aromatic ring. By this mechanism, conducting the polymerization only on the dendrimer is theoretically possible in a manner akin to graft polymerization. Recent investigation of catalyst-transfer polymerization revealed that dialkylbiarylphosphine ligand–Pd(0) species works as the excellent initiator to afford the desired polymer with a narrow polydispersity index and high molecular weight^39–41^.

**Fig. 3:**
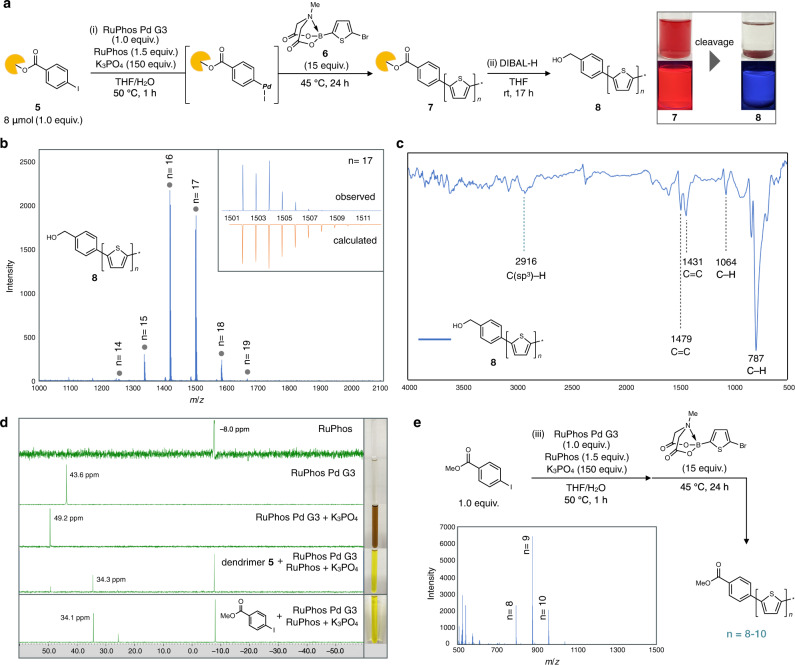
On-dendrimer synthesis of polythiophenes. **a** Synthesis of polythiophenes **7** and **8**. Reagents and conditions: (i) RuPhos Pd G3 (1.0 equiv.), RuPhos (1.5 equiv.), K_3_PO_4_ (150 equiv.), THF/H_2_O, 50 °C, 1 h; then **6** (15 equiv.), 45 °C, 24 h; (ii) DIBAL-H (excess), THF, room temperature, 17 h. Abbreviations: RuPhos Pd G3, (2-dicyclohexylphosphino-2′,6′-diisopropoxy-1,1′-biphenyl)[2-(2′-amino-1,1′-biphenyl)]palladium(II) methanesulfonate; RuPhos, 2-dicyclohexylphosphino-2′,6′-diisopropoxybiphenyl; DIBAL-H, diisobutylaluminium hydride. Chloroform solution of dendrimer-ligated polythiophene **7** exhibited red color and orange fluorescence under 365 nm UV irradiation (left picture). Unsubstituted polythiophene **8** in chloroform did not exhibit red fluorescence under 365 nm UV irradiation (right picture). **b** LDI-TOF mass spectrum of **8** (blue line) and calculated isotopic pattern of **8** (*n* = 17) (orange line). **c** IR spectrum of **8**. **d** ^31^P-NMR analysis on the formation of initiator. **e** Off-dendrimer synthesis of polythiophene. Reagents and conditions: (iii) RuPhos Pd G3 (1.0 equiv.), RuPhos (1.5 equiv.), K_3_PO_4_ (150 equiv.), THF/H_2_O, 50 °C, 1 h; then **6** (15 equiv.), 45 °C, 24 h. Source data are provided as a Source Data file.

We began by investigating the on-dendrimer polythiophene synthesis following Choi’s conditions^39^ (Fig. 3a). Thus, the dendritic aryl iodide **5** was treated with RuPhos Pd G3 precatalyst (RuPhos = 2-dicyclohexylphosphino-2′,6′-diisopropoxy-1,1′-biphenyl) in the presence of RuPhos (1.5 equiv.) and K_3_PO_4_ (150 equiv.) in THF/H_2_O to generate the corresponding oxidative-addition complex in situ. The polymerization reaction was initiated by adding 15 equiv. of 5-bromo-2-thiopheneboronic acid MIDA ester (**6**) (MIDA = *N*-methyliminodiacetic acid) to the resulting oxidative-addition complex. The catalyst-transfer polymerization proceeded smoothly under these conditions and the reaction mixture became a completely clear red solution after 24 h heating, indicating the formation of polythiophene on the dendrimer that should remain in solution. The reaction mixture was quenched by the addition of 1 M HCl aq., extracted with chloroform and precipitated with acetone, then the dendrimer-ligated polythiophene **7** was obtained as a red solid in 97% yield. Albeit detailed characterization has not been realized due to broad signals presumably derived from intermolecular interaction of **7** in the concentration for ^1^H NMR measurement, the analysis of model reaction with 2,2′-bithiophene boronic acid ester proved the bond formation between dendrimer support and thiophene (see Section 6-4 in SI for details).

To better understand the solution behavior of dendrimer-ligated polythiophene **7**, the hydrodynamic radius of **7** was determined by dynamic light scattering measurement (Supplementary Fig. 8). The particle radius derived from the sphere approximation of chloroform solution of **7** (5.0 mg/mL) was estimated to be 3.6 nm. Given that the theoretical length of polythiophene with 15 thiophene rings is 5.7 nm, dendrimer-ligated polythiophene **7** does not form multimolecular aggregate (e.g. supramolecular polymer or insoluble aggregate) in at least 5.0 mg/mL in chloroform. The formation of a main chain-unsubstituted polythiophene structure in **7** was verified by liberating unsubstituted polythiophene from the dendrimer support by reduction of the ester linker moiety. To achieve this, the dendrimer-ligated polythiophene **7** was treated with an excess amount of diisobutylaluminum hydride (DIBAL-H) in THF. After the cleavage reaction, the red color of the solution disappeared and an insoluble red precipitate was generated (Fig. 3a). Laser desorption/ionization-time of flight (LDI-TOF) mass spectroscopy analysis of the precipitate unambiguously exhibited the mass distribution corresponding to unsubstituted polythiophene **8** with 14−19 thiophene rings (Fig. 3b). Mass distribution shows good consistency with the chosen monomer/initiator ratio of 15:1 and the end-group was also confirmed to be a benzylic alcohol structure by the mass spectrum. Furthermore, the isotopic distribution of unsubstituted polythiophene **8** shows good agreement with the calculated distribution (*n* = 17). These results indicate that the catalyst-transfer polymerization reaction successfully proceeded on the dendrimer **5**. Moreover, as unsubstituted polythiophene containing more than 7 thiophene rings is known to be essentially insoluble^42^, the current results also clearly show that bare polythiophene with up to 19 thiophene rings can be solubilized by our dendrimer support.

To investigate the limit of dendrimer-supported synthesis in terms of the polymer length, polymerization reaction was conducted with increased monomer/initiator ratio. While full identification of the product has not been successful due to severe limitation in analytical methods for high molecular weight polymers (especially LDI-TOF MS), on-dendrimer polymerization with at least up to 50 equiv. of monomer proceeded, judging from its clear solution and ^1^H-NMR spectrum (see Section 6-3 in SI for details).

Our method grants access to essentially unsubstituted polythiophene in rather high purity. Thus, we conducted infrared spectroscopy (IR) analysis of dendrimer-free bare polythiophene **8** by the KBr method (Fig. 3c). The out-of-plane C–H stretching mode is clearly observed at 787 cm^–1^. Other characteristic absorption bands at 1064, 1431 and 1479 cm^–1^ are assigned to C–H aromatic in-plane bending vibration, C=C symmetric vibration and C=C asymmetric vibration of thiophene ring, respectively^43^. In addition, C(sp^3^)–H stretching vibration of the chain-end benzylic alcohol moiety was also observed at 2917 cm^–1^.

We also investigated the reaction mechanism (initiator formation) by ^31^P-NMR analysis to verify our hypothesis that polymerization proceeds on the dendrimer support (Fig. 3d). In THF, signals of RuPhos and RuPhos Pd G3 precatalyst were observed at −8.0 ppm and 43.6 ppm, respectively. When the precatalyst was activated by K_3_PO_4_, the mixture turned brown and new signal at 49.2 ppm was clearly observed, indicating that the precatalyst was transformed into the active palladium species. Next, we repeated the same process in the presence of 1.0 equiv. of dendritic aryl iodide **5** and additional RuPhos ligand. A signal at 34.3 ppm emerged and the distinct difference of solution color implies the formation of oxidative-addition complex. When methyl 4-iodobenzoate was used instead of **5**, the signal at 34.1 ppm was also observed as the major signal. Thus, we conclude that the polymerization-active, oxidative-addition complex was successfully formed on the dendrimer support and it works as the initiator in the thiophene polymerization.

To confirm the critical role of the dendrimer, we also conducted control experiments without using the dendrimer support (Fig. 3e). When methyl 4-iodobenzoate was used instead of **5** as the initiator, red precipitate was generated after polymerization reaction. The red solid was insoluble to various organic solvents such as chloroform and THF. The LDI-TOF mass spectrum shows signals corresponds to oligothiophene exclusively with 8–10 thiophene units and no signal more than 11 thiophene units was observed. These results strongly support our concept of dendrimer-enabled, solution-phase synthesis of bare aromatic polymers.

### On-dendrimer synthesis of poly(*para*-phenylene) and other bare aromatic polymers

**Fig. 4:**
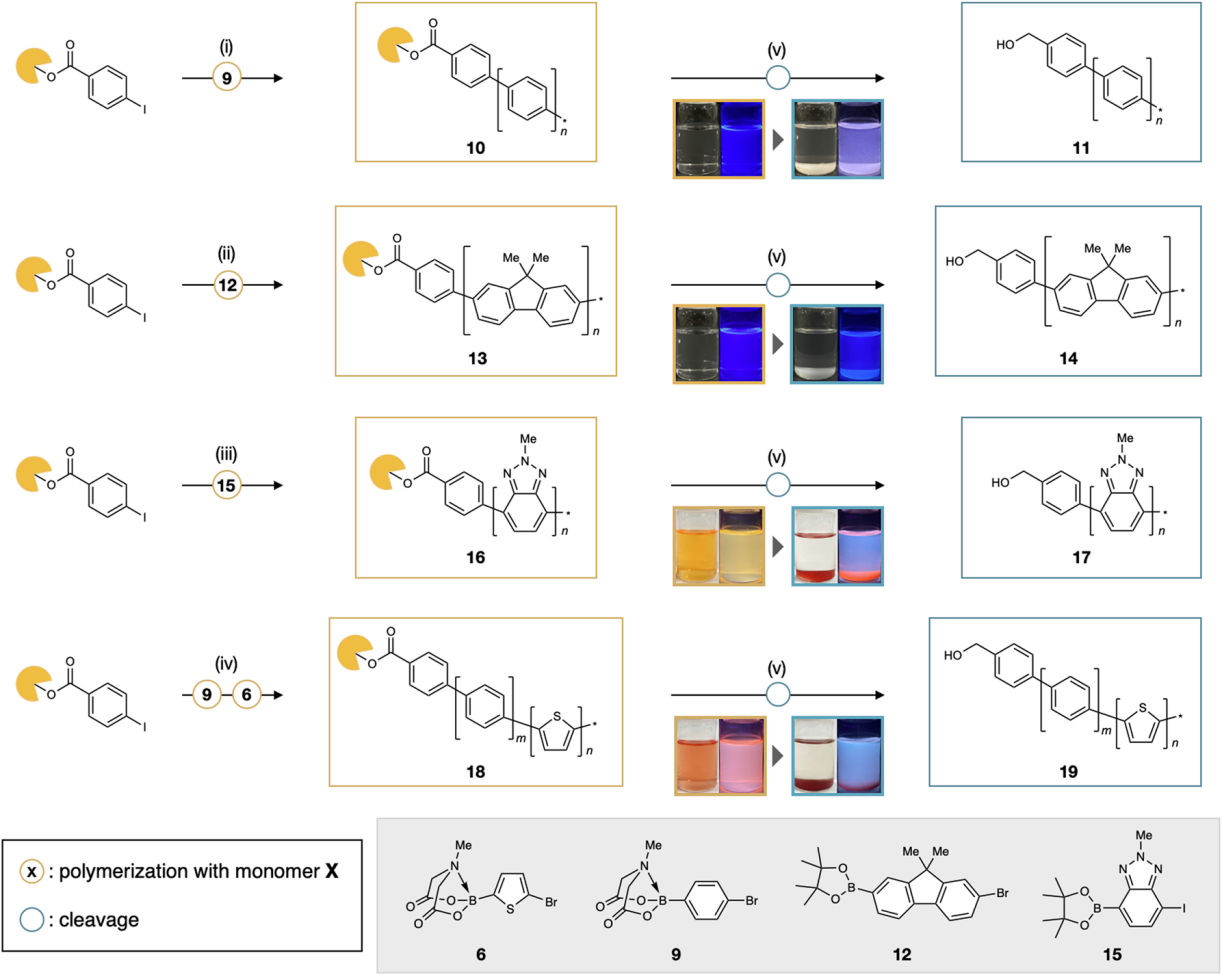
Synthesis of dendrimer-ligated and dendrimer-free bare aromatic polymers. Reagents and conditions: (i) RuPhos Pd G3 (1.0 equiv.), RuPhos (1.5 equiv.), K_3_PO_4_ (150 equiv.), THF/H_2_O, 50 °C, 1 h; then **9** (15 equiv.), 50 °C, 24 h; (ii) ^*t*^Bu_3_P Pd G2 (1.0 equiv.), 2 M Na_2_CO_3_ aq. (0.35 mL), THF, room temperature, 1 h; then **12** (9.0 equiv.), 0 °C, 17 h; (iii) RuPhos Pd G3 (1.0 equiv.), RuPhos (1.5 equiv.), K_3_PO_4_ (150 equiv.), THF/H_2_O, 50 °C, 1 h; then **15** (10 equiv.), 40 °C, 24 h; (iv) RuPhos Pd G3 (1.0 equiv.), RuPhos (1.5 equiv.), K_3_PO_4_ (150 equiv.), THF/H_2_O, 50 °C, 1 h; then **9** (7.0 equiv.), 50 °C, 18 h; then **6** (7.0 equiv.), 45 °C, 12 h; (v) DIBAL-H (excess), THF, room temperature, 17–24 h. ^*t*^Bu_3_P Pd G2 chloro[(tri-*tert*-butylphosphine)-2-(2-aminobiphenyl)]palladium(II). The pictures in yellow marks are chloroform solutions of dendrimer-ligated aromatic polymers and their fluorescence under 365 nm UV irradiation. The pictures in green marks are unsubstituted aromatic polymers precipitating in chloroform, which are taken under 365 nm UV irradiation.

Having established the proof-of-concept synthesis of bare polythiophenes, we embarked on the on-dendrimer synthesis of other aromatic polymers including historical and benchmark poly(*para*-phenylene)s (PPPs) and other previously inaccessible bare aromatic polymers (Fig. 4). At the outset, PPP was synthesized by the same catalyst-transfer polymerization reaction with monomer **9**. The thus-obtained dendrimer-ligated PPP **10** was highly soluble in common organic solvents such as chloroform, THF and hexane and shows blue fluorescence. LDI-TOF mass spectroscopy after cleavage reaction shows a mass distribution of main chain-unsubstituted PPP **11** with 6–16 benzene rings (Supplementary Fig. 10). Moreover, IR spectroscopy of **11** shows an identical absorption band to previously synthesized unsubstituted PPP (see SI for details)^15^. The on-dendrimer synthesis can be applied not only to rather familiar PPPs and PTs^11–16,44,45^, but also to other bare aromatic polymers with unprecedented structures. For example, dendrimer-ligated bare polyfluorene (d-PFL) **13** and polybenzotriazole (d-PBT) **16** were synthesized by catalyst-transfer polymerization of monomer **12** and **15** on the same dendrimer support^40,46^. The dendrimer-cleaving reactions of **13** and **16** afforded insoluble white and orange solid, respectively. Their LDI-TOF mass spectra showed a mass distribution corresponding to unsubstituted polyfluorene **14** and polybenzotriazole **17** (Supplementary Figs. 11, 12). Furthermore, the synthesis of block copolymer (d-PPP-PT) **18** containing benzene and thiophene units was also demonstrated. LDI-TOF mass spectroscopy after the cleavage reaction shows a complicated mass distribution indicating the generation of unsubstituted phenylene-thiophene block copolymer **19** (Supplementary Figs. 13, 14).

### Photophysical properties of bare aromatic polymers

**Fig. 5:**
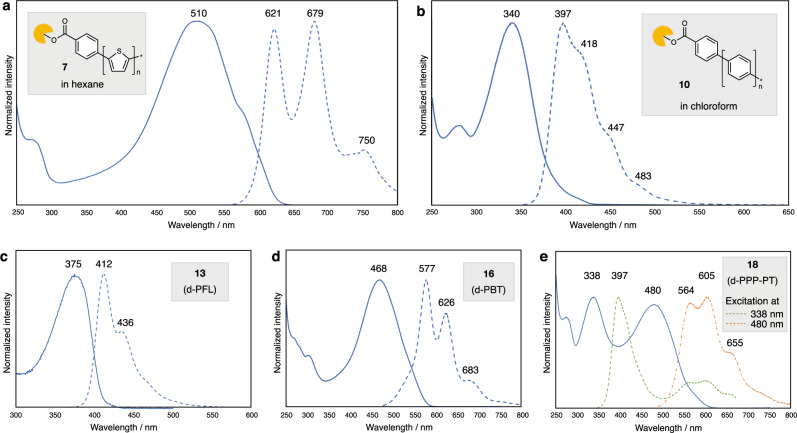
Photophysical properties of polythiophenes 7 and 8. **a** UV/vis absorption (solid line) and fluorescence (broken line) spectra of the hexane solution of **7**. Fluorescence spectrum was taken upon excitation at 510 nm. **b** UV/vis absorption (solid line) and fluorescence (broken line) spectra of the chloroform solution of **10**. Fluorescence spectrum was taken upon excitation at 340 nm. **c** UV/vis absorption (solid line) and fluorescence (broken line) spectra of the chloroform solution of **13** (d-PFL). Fluorescence spectrum was taken upon excitation at 360 nm. **d** UV/vis absorption (solid line) and fluorescence (broken line) spectra of the chloroform solution of **16** (d-PBT). Fluorescence spectrum was taken upon excitation at 468 nm. **e** UV/vis absorption (solid line) and fluorescence (broken line) spectra of the chloroform solution of **18** (d-PPP-PT). Fluorescence spectrum was taken upon excitation at 338 nm (green broken line) and 480 nm (orange broken line). Source data are provided as a Source Data file.

As already mentioned, long and bare polythiophenes have remained synthetically out of reach due to the solubility problem. Thus, we are now in a position to experimentally uncover a range of properties of bare polythiophene structures in solution, such as UV/vis absorption and fluorescence. In a hexane solution, dendrimer-ligated polythiophene **7** shows an absorption maximum at 510 nm and exhibits orange fluorescence, with peaks at 621, 679, 750 nm (Fig. 5a). On the other hand, regioregular poly(3-hexylthiophene) (P3HT) (*M*_n_ = 30–40 kg mol^–1^) is known to have its maximum absorption at 455 nm^47^. One of the reasons for this stark difference between our bare polythiophene **7** and P3HT is presumably derived from deplanarization of polymer backbone in P3HT caused by introduced alkyl chain. Experimental absorption maxima of unsubstituted polythiophene^48^ (> 36-mer) and planar polythiophenes^49,50^ (18-mer, 32-mer) are 536 nm, 517 nm and ca. 530 nm, respectively. These values indicate that polythiohene backbone of **7** has a planar structure similar to its unsubstituted analog. In addition, the photophysical properties of dendrimer-ligated PPP **10** were also investigated (Fig. 5b). The maximum absorption wavelength appears at 340 nm, and the fluorescence spectrum exhibits emission maximum at 397 nm and shoulder peaks at 418, 447 and 483 nm. These values are highly consistent with those of solid-state unsubstituted PPP reported by Müllen^15^. It is worth noting that our soluble bare PPP shows similar characteristics to its insoluble unsubstituted analog. In other words, dendrimer support **5** can grant high solubility to bare aromatic polymers without much property change. Furthermore, photophysical properties of other bare aromatic polymers (d-PFL, d-PBT, d-PPP-PT) were measured. Polymers d-PFL and d-PBT have absorption maxima at 375 nm and 468 nm, and show blue (*λ*_max_ = 412 nm) and yellow (*λ*_max_ = 577 nm) fluorescence, respectively. In case of block copolymer d-PPP-PT, absorption maxima of 338 nm and 480 nm were observed, which respectively derive from oligo(*para*-phenylene) and oligothiophene units. Overall, photophysical properties of unprecedented bare aromatic polymers were revealed by our dendrimer-supported strategy (Fig. 5c–e).

### Transfer of bare aromatic polymers to other functional materials

**Fig. 6:**
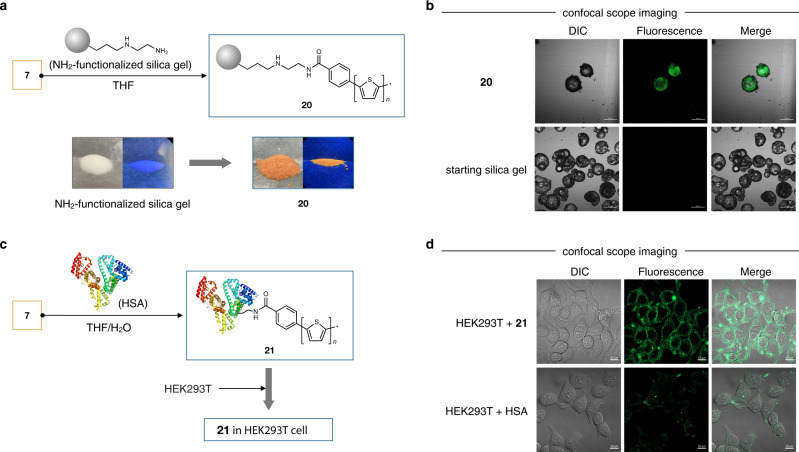
Transfer of bare aromatic polymers to other functional materials. **a** Synthesis of polythiophene-attached silica gel **20**. Starting white NH_2_-functionalized silica gel shows no fluorescence. Red-colored **20** shows orange fluorescence under 365 nm UV irradiation. **b** Confocal scope images of **20** (top) and starting silica gel (bottom). **c** Synthesis of polythiophene-attached albumin protein **21** and introducing **21** into HEK293T cells. **d** Confocal scope images of HEK293T cells with **21** (top) and HSA protein (bottom).

By taking advantage of the high solubility and steric protection conferred by our diterpenoid-based dendrimer support, a range of bare aromatic polymer structures can now be easily synthesized, purified, spectroscopically analyzed, and handled in solution, which should pave the way for their application in these forms. While on-demand release of insoluble aromatic polymers is also possible (Figs. 3a, 4), we discovered that these soluble dendrimer-ligated aromatic polymers can serve as polymer-transferring reagents. In doing so, the attractive structures and associated functions of bare aromatic polymers can be easily installed into a range of functional materials. In this work, we demonstrate two representative examples merging bare polythiophenes with inorganic silica gel and biological protein (Fig. 6).

When dendrimer-ligated polythiophene **7** and amine-functionalized silica gel was stirred at 66 °C in THF for 24 h, polythiophene-attached silica gel **20** was obtained (Fig. 6a). Confocal microscopic imaging of resulting red-colored silica gel **20** showed that the silica gel surface exhibits fluorescence by excitation at 488 nm, whereas the precursor silica gel has no fluorescence (Fig. 6b). In order to rule out nonspecific adsorption of polythiophene to silica gel, IR spectroscopy was conducted (Supplementary Fig. 17). The hybrid silica gel **20** shows a new band around 1602 cm^–1^ which corresponds to an amide C=O stretching mode^51^. Furthermore, the C(*sp*^**3**^)–H stretching mode of the long alkyl chains in the dendrimer support in **7** was not observed in the IR spectrum of **20**. These results support that bare polythiophene was successfully transferred from dendrimer to silica gel. This surface functionalization can be potentially applicable to the preparation of smart surfaces^52^.

Separately, our bare polythiophene backbone was also transferred to a protein. Human serum albumin (HSA), the most abundant plasma protein, was reacted with dendrimer-ligated polythiophene **7** in THF/phosphate-buffered saline (PBS) at 37 °C (Fig. 6c). Successful covalent transfer of bare polythiophene to HSA (most likely at lysine residues) was confirmed in gel fluorescence analysis (Supplementary Fig. 18), albeit in MALDI-TOF MS analysis polythiophene-conjugated HSA was not detected due to its low transfer efficiency. With the polythiophene-conjugated HSA in hand, its dynamics in a human cellular environment were investigated. Human embryonic kidney 293T (HEK293T) cells were incubated with a PBS solution of the polythiophene-conjugated HSA at 37 °C. Confocal microscopic images showed fluorescence of polythiophene in the cellular membrane and cytoplasm of HEK293T cell^53^, indicating that bare polythiophene can be internalized in the cell (Fig. 6d). In addition, the cell was healthy even in the presence of polythiophene-conjugated HSA as judged from its cell morphology. This result strongly indicates the possibility for bare aromatic polymers to be utilized in biological applications.

In summary, we have developed a synthetic method for main chain-unsubstituted bare aromatic polymers, which employs a diterpenoid-based dendrimer as a solubilizing group. Bare aromatic polymers containing polythiophene, poly(*para*-phenylene), polyfluorene, polybenzotriazole and poly(*para*-phenylene)-*block*-polythiophene have been successfully synthesized and their properties were unveiled. The present study not only enables the hitherto difficult synthesis of bare aromatic polymers, but also illuminates the power and value of our synthetic methodology, which provides access to versatile aggregative molecules such as large planar aromatic molecules and giant nanocarbons. Taking advantage of their solubility in various solvents, the hybridization of bare aromatic polymers with inorganic- and bio-materials has also been achieved. Due to their excellent photophysical and conductive properties, aromatic polymers have long been in great demand for organic-inorganic hybrid materials and bioconjugates^54–56^. The creation of unprecedented materials will be realized by transferring bare aromatic polymers, which may open a new vista of materials science. Our strategy can overcome the solubility problem which stands widely in chemistry^57^ and holds promise for new materials design and applications.

## Methods

### Synthesis of dendrimer-ligated polythiophene 7

Oxidative-addition complex formation: To a screw capped tube was added K_3_PO_4_ (257.9 mg, 1.2 mmol) and dried-up under vacuum. Dendrimer support **5** (66.7 mg, 8.0 μmol), RuPhos Pd G3 (6.7 mg, 8.0 μmol) and RuPhos (5.6 mg, 12.0 μmol) were added to the screw capped tube. The tube was filled with nitrogen followed by addition of THF (2.0 mL) and H_2_O (0.72 mL). The reaction mixture was stirred at 50 °C for 1 h. The color of thus-obtained solution was clear yellow.

Polymerization reaction: To a 50-mL two-necked flask was added thiophene monomer **6** (38.1 mg, 120 μmol). The flask was filled with nitrogen and dry THF (18.0 mL) was added. Pre-prepared catalyst solution was added in one portion to the flask by syringe. 2.0 mL of THF was used for washing the vessel. The reaction mixture was stirred at 45 °C for 24 h. The reaction was quenched by addition of 1 M HCl aq. at room temperature. The mixture was extracted with CHCl_3_ (ca. 50 mL × 3) and combined organic layers were dried over Na_2_SO_4_. It was filtrated by membrane filter (pore size: 0.2 μm) and filtrate was concentrated under reduced pressure. Resulting crude product was reprecipitated from acetone to afford **7** (74.2 mg) as a red solid.

### Reporting summary

Further information on research design is available in the Nature Research Reporting Summary linked to this article.

## Supplementary information

Supplementary Information

Reporting Summary

## Data Availability

Materials and methods, experimental procedures, photophysical studies and NMR spectra are available in the Supplementary Information or from the corresponding authors upon request.  Source data are provided with this paper.

## Source data

Source Data
