# Supplementary material for: Synthesis, properties, and material hybridization of bare aromatic polymers enabled by dendrimer support
Source: Nat Commun. 2022 Sep 16;13:5358. doi: 10.1038/s41467-022-33100-7 (PMC9481634; doi:10.1038/s41467-022-33100-7)
Supplement: Supplementary file 1 — Supplementary Information [file 41467_2022_33100_MOESM1_ESM.pdf]

Supplementary Information

Synthesis, properties, and material hybridization of bare aromatic polymers enabled by dendrimer support

Shusei Fujiki,[1] Kazuma Amaike,[1] Akiko Yagi,[1,2,]* and Kenichiro Itami[1,2,]*

[1] *Graduate School of Science, Nagoya University, Chikusa, Nagoya 464-8602, Japan*

[2] *Institute of Transformative Bio-Molecules (WPI-ITbM), Nagoya University, Chikusa, Nagoya 464-8602, Japan*

E-mail: itami@chem.nagoya-u.ac.jp (KI), yagi.akiko@itbm.nagoya-u.ac.jp (AY)

Table of Contents

1.	Materials and Methods	1
2.	Preparation of Dendrimer Support	2–15
3.	Preparation of Monomer	16–20
4.	Polymerization Reaction on Dendrimer Support	21–23
5.	Cleavage Reaction between Dendrimer Support and Aromatic Polymer	24–25
6.	Control Experiment of Polymerization	26–33
7.	Spectroscopic Measurements	34–41
8.	Material Hybridization	42–44
9.	Computational Data	45–46
10.	Supplementary References	47–48
11.	^{1}H and ^{13}C NMR Spectra	49–82

1. Materials and Methods

Unless otherwise noted, all reactants or reagents including dry solvents were obtained from commercial suppliers and used as received. Tetrahydrofuran (THF), toluene, and *N*,*N*-dimethylformamide (DMF) were purified by passing through a solvent purification system (Glass Contour). $CuBr(PPh_3)_3$ was synthesized according to the procedure reported in the literature[1]. Amino-functionalized silica gel was purchased from Fuji Silysia Chemical Ltd. All reactions were performed with dry solvents under an atmosphere of nitrogen or argon in dried glassware using standard vacuum-line techniques. All work-up and purification procedures were carried out with reagent-grade solvents under air.

Analytical thin-layer chromatography (TLC) was performed using E. Merck silica gel 60 F254 precoated plates (0.25 mm). The developed chromatograms were analyzed by a UV lamp (254 or 365 nm). Flash column chromatography was performed with KANTO Silica Gel 60N (spherical, neutral, 40-100 μm) or Biotage Isolera® equipped with Biotage SNAP Cartridge KP-Sil columns. Preparative recycling gel permeation chromatography (GPC) was performed with a JAI LC-9260 II NEXT instrument equipped with JAIGEL-2HR columns using chloroform as an eluent. The high-resolution mass spectra (HRMS) were obtained from a Thermo Fisher Scientific Exactive (atmospheric pressure chemical ionization, APCI or electrospray ionization, ESI), a JEOL JMS-T100GCV (Direct EI), a JEOL JMS-700 (fast atom bombardment, FAB) with 3-nitrobenzyl alcohol (NBA) as matrix and JEOL JMS-S3000 SpiralTOF using polyethylene glycol mixture as external standard (MALDI and LDI). Nuclear magnetic resonance (NMR) spectra were recorded on a JEOL ECS-600 (^{1}H 600 MHz, ^{13}C 150 MHz) spectrometer or a JEOL ECS-500 (^{1}H 500 MHz, ^{13}C 125 MHz) spectrometer. Chemical shifts for ^{1}H NMR are expressed in parts per million (ppm) relative to $CDCl_3$ (7.26 ppm), dimethyl sulfoxide (DMSO)-d_6 (2.50 ppm) or tetrachloroethane (TCE)-d_2 (6.00 ppm). Chemical shifts for ^{13}C NMR are expressed in parts per million (ppm) relative to $CDCl_3$ (77.16 ppm) or DMSO-d_6 (39.52 ppm). Data are reported as follows: chemical shift, multiplicity (s = singlet, d = doublet, t = triplet, dd = doublet of doublets, sep = septet, m = multiplet, br = broad), coupling constant (Hz), and integration. Source data are provided with this paper.

2. Preparation of Dendrimer Support

2-1. Synthesis of Alkyl Chain

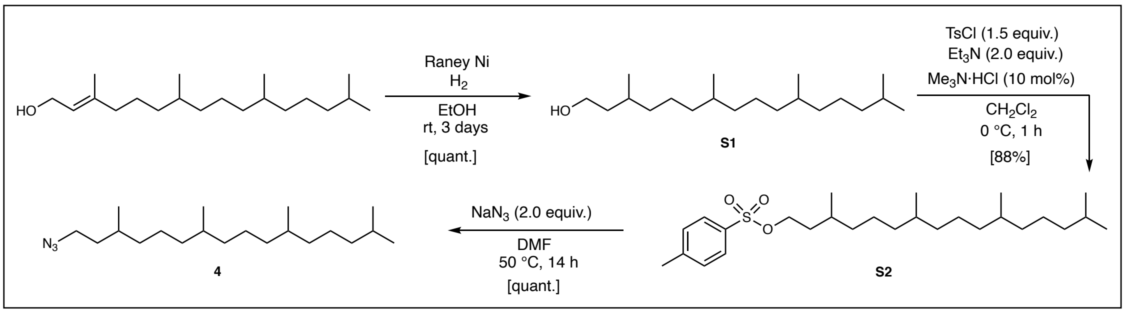

3,7,11,15-tetramethylhexadecan-1-ol (S1)

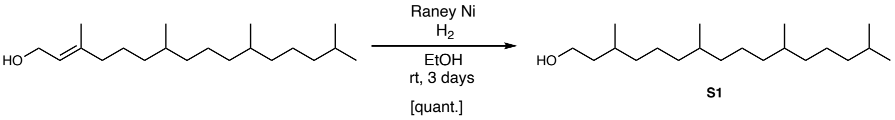

To a 300-mL flask were added phytol (80 mL), ethanol (150 mL) and Raney Ni (50% in H_2O, 7 mL). This suspension was stirred at room temperature for 3 days under hydrogen atmosphere. Raney Ni was carefully removed by Celite® filtration and washed by ethanol. Filtrate was evaporated to afford **S1** in quantitative yield as a colorless oil. It was used for the next reaction without further purification. ^{1}H NMR spectrum of **S1** agrees with reported data[2].

3,7,11,15-tetramethylhexadecyl 4-methylbenzenesulfonate (S2)

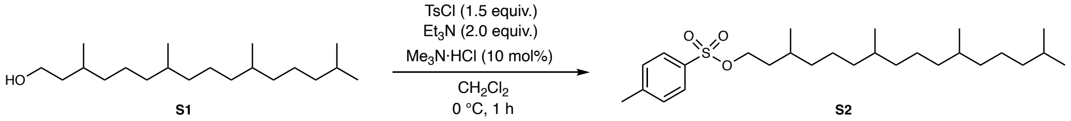

To a 200-mL flask were added **S1** (15 g, 50 mmol), CH_2Cl_2 (50 mL), Et_3N (14 mL, 100 mmol) and Me_3N•HCl (475 mg, 5 mmol). After cooling to 0 °C, *p*-toluenesulfonyl chloride (TsCl) (14 g, 75 mmol) dissolved in 50 mL of CH_2Cl_2 was slowly added to the flask. The reaction mixture was stirred at 0 °C for 2 h. In order to remove excess TsCl, *N,N*-dimethylethylenediamine (5.0 mL) was added to the reaction mixture and the mixture was further stirred at 0 °C for 30 min. Then water was added to reaction mixture. Aqueous layer was extracted with CH_2Cl_2, dried over Na_2SO_4 and then concentrated *in vacuo*. The crude product was purified by silica gel column chromatography (eluent: hexane/AcOEt = 100:0 → 85:15) to afford **S2** (19.9 g, 88%) as a colorless oil.

^{1}H NMR (600 MHz, $CDCl_3$) δ 7.79 (d, *J* = 8.2 Hz, 2H), 7.34 (d, *J* = 8.3 Hz, 2H), 4.08–3.95 (m, 2H), 2.45 (s, 3H), 1.68–0.96 (m, 24H), 0.90–0.78 (m, 15H).

^{13}C NMR (150 MHz, $CDCl_3$) δ 144.75, 133.34, 129.92, 128.02, 69.26, 39.49, 37.58, 37.52, 37.41,

37.32, 37.12, 37.09, 35.90, 35.82, 32.90, 32.87, 29.33, 28.11, 24.94, 24.61, 24.33, 22.86, 22.76, 21.78, 19.88, 19.82, 19.80, 19.73, 19.32, 19.25.

HRMS (ESI^+): calcd. for $C_{27}H_{48}NaO_3S$ ($[M+Na]^+$): 475.3216, found: 475.3212 (error: 0.84 ppm).

1-azido-3,7,11,15-tetramethylhexadecane (4)

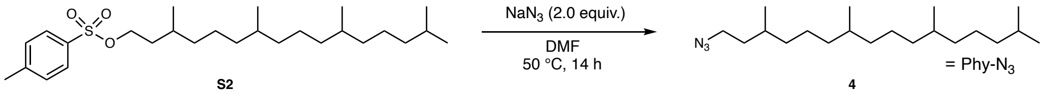

To a 300-mL flask were added **S2** (6.78 g, 15 mmol) and DMF (75 mL) followed by addition of NaN_3 (1.95 g, 30 mmol). The reaction mixture was stirred at 50 °C for 14 h. After cooling to room temperature, H_2O (ca. 150 mL) was added and the mixture was extracted with diethyl ether three times, washed with H_2O and brine. Combined organic layer was dried over $MgSO_4$ and then concentrated *in vacuo* to afford **4** (4.93 g) as a colorless oil in quantitative yield. It was used for the next reaction without further purification. ^{1}H NMR spectrum of **4** agrees with reported data[3].

2-2. Synthesis of Dendron 2

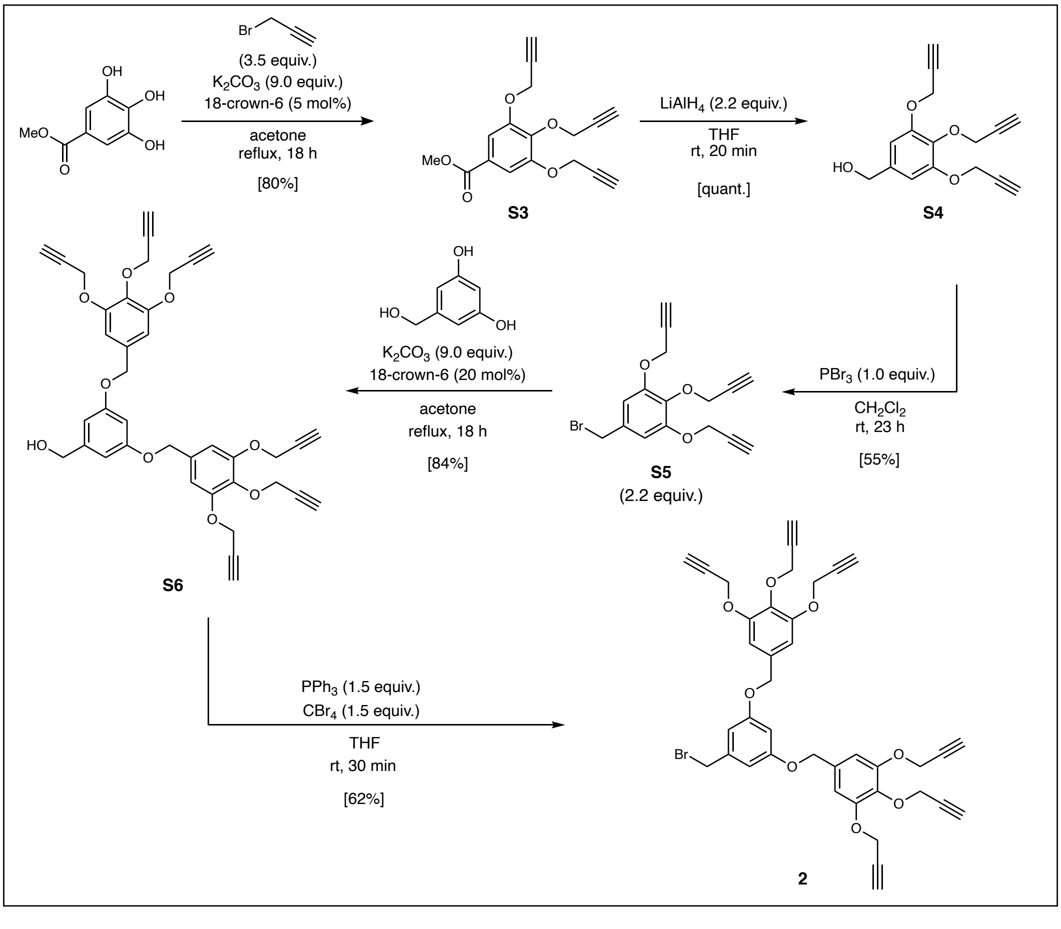

Note: **S3-S6** and dendron **2** were synthesized based on the procedures reported in the literature[4,5].

methyl 3,4,5-tris(prop-2-yn-1-yloxy)benzoate (S3)

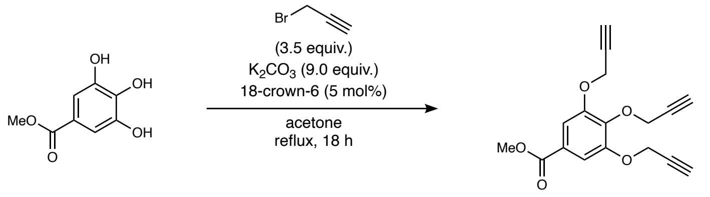

S3

To a 500-mL two-necked flask were added methyl gallate (9.23 g, 50 mmol), K_2CO_3 (62.36 g, 451 mmol) and 18-crown-6 (655 mg, 2.5 mmol). The flask was filled with nitrogen followed by addition of acetone (150 mL). Propargyl bromide (9.2 M in toluene, 19.2 mL, 175 mmol) was added to the vigorously stirred solution. The reaction mixture was stirred for 18 h at 60 °C. Acetone was removed by evaporation and then water was added until all inorganic salts dissolve. The aqueous solution was extracted with $CHCl_3$ for three times. Combined organic layer was washed with brine, dried over Na_2SO_4 and then concentrated *in vacuo*. Crude product was purified by recrystallization from hot MeOH to afford **S3** (11.8 g, 80%) as a white solid. ^{1}H NMR spectrum of **S3** agrees with reported data[4].

(3,4,5-tris(prop-2-yn-1-yloxy)phenyl)methanol (S4)

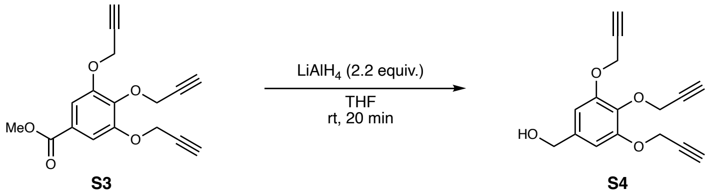

S3 **S4**

To a flame dried 500-mL two-necked flask was added THF (300 mL). $LiAlH_4$ (3.34 g, 88 mmol) was slowly added to the flask under gentle nitrogen flow and then flask was cooled to 0 °C. **S3** (11.8 g, 40 mmol) was dissolved to THF (60 mL) under inert atmosphere and the resultant solution was added dropwise to the $LiAlH_4$ suspension. Then the reaction mixture was warmed up to room temperature and stirred for 12 h at room temperature. In order to quench the reaction, H_2O (3.3 mL), 15% NaOH aq. (3.3 mL) and H_2O (9.9 mL) was added at 0 °C in this order. The solution

was stirred at room temperature until supernatant of the solution becomes clear. The white precipitate was removed by filtration and filtrate was concentrated under reduced pressure to afford pure **S4** (10.8 g, quant.) as a white solid. ^{1}H NMR spectrum of **S4** agrees with reported data[4].

5-(bromomethyl)-1,2,3-tris(prop-2-yn-1-yloxy)benzene (S5)

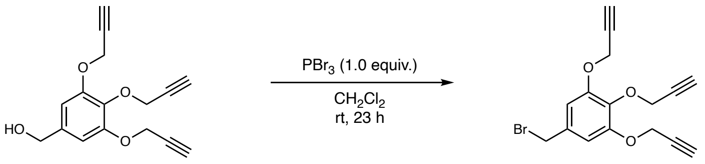

S4 S5

To a flame dried 300-mL two-necked flask was added **S4** (9.72 g, 36 mmol). The flask was filled with nitrogen followed by addition of CH_2Cl_2 (150 mL). PBr_3 (3.4 mL, 36 mmol) was slowly added to the solution. The reaction mixture was stirred for 23 h at room temperature. H_2O was slowly added to the reaction mixture at 0 °C to quench the reaction and aqueous layer was extracted with CH_2Cl_2 for three times. Combined organic layer was washed with sat. $NaHCO_3$ aq. until neutralized. Organic layer was dried over Na_2SO_4 and concentrated under reduced pressure. Crude product was purified by reprecipitation from $CHCl_3$/hexane to afford **S5** (6.62 g, 55%) as a white solid. ^{1}H NMR spectrum of **S5** agrees with reported data[4].

(3,5-bis((3,4,5-tris(prop-2-yn-1-yloxy)benzyl)oxy)phenyl)methanol (S6)

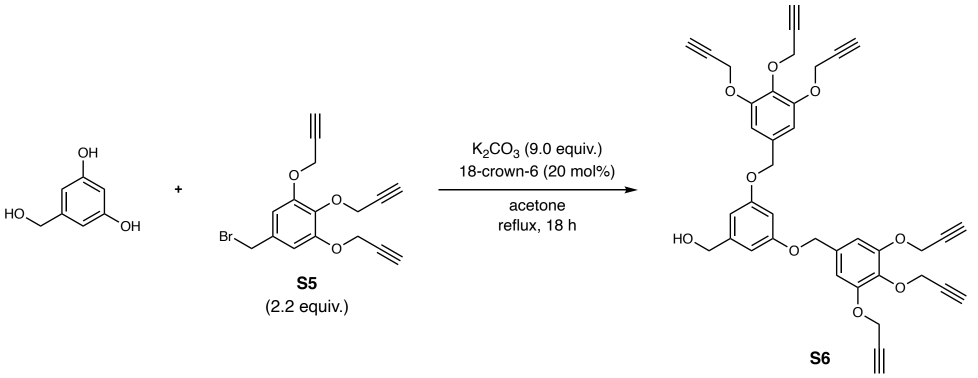

To a 200-mL two-necked flask were added 3,5-dihydroxybenzyl alcohol (1.74 g, 12.4 mmol), 18-crown-6 (655 mg, 2.5 mmol) and K_2CO_3 (15.4 g, 111 mmol). The flask was filled with nitrogen followed by addition of acetone (70 mL). **S5** (9.10 g, 27.3 mmol) was added to the flask

under nitrogen flow and then reaction mixture was stirred for 18 h at 65 °C. The reaction mixture was concentrated under reduced pressure. H_2O was added to the flask until all inorganic salts dissolve and the aqueous solution was extracted with AcOEt for three times. Combined organic layer was washed with brine, dried over Na_2SO_4 and concentrated under reduced pressure. Crude product was purified by silica gel column chromatography (eluent: CH_2Cl_2/AcOEt = 20:1 → 15:1) to afford **S6** (6.69 g, 84%) as a white solid. ^{1}H NMR spectrum of **S6** agrees with reported data[4].

5,5'-(((5-(bromomethyl)-1,3-phenylene)bis(oxy))bis(methylene))bis(1,2,3-tris(prop-2-yn-1-yloxy)benzene) (2)

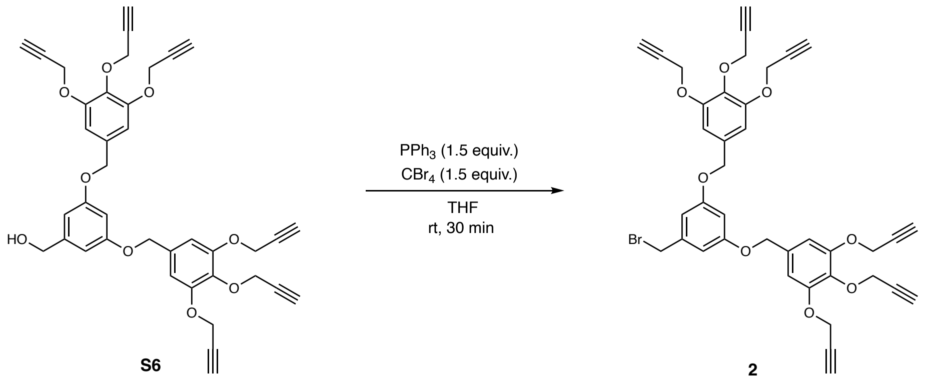

To a 200-mL two-necked flask were added **S6** (7.02 g, 10.9 mmol) and CBr_4 (5.40 g, 16.3 mmol). The flask was filled with nitrogen followed by addition of THF (40 mL). PPh_3 (4.28 g, 16.3 mmol) was added to the flask under nitrogen flow at 0 °C. The reaction mixture was stirred for 30 min at room temperature. The reaction mixture was concentrated under reduced pressure and triphenylphosphine oxide was roughly removed by passing through short silica gel pad (eluent: hexane → CH_2Cl_2). Thus-obtained crude product was purified by silica gel column chromatography (eluent: hexane/CH_2Cl_2 = 1:5 → 100:0) to afford **2** (4.82 g, 62%) as a white solid. ^{1}H NMR spectrum of **2** agrees with reported data[4].

2-3. Synthesis of Dendrimer Core 1

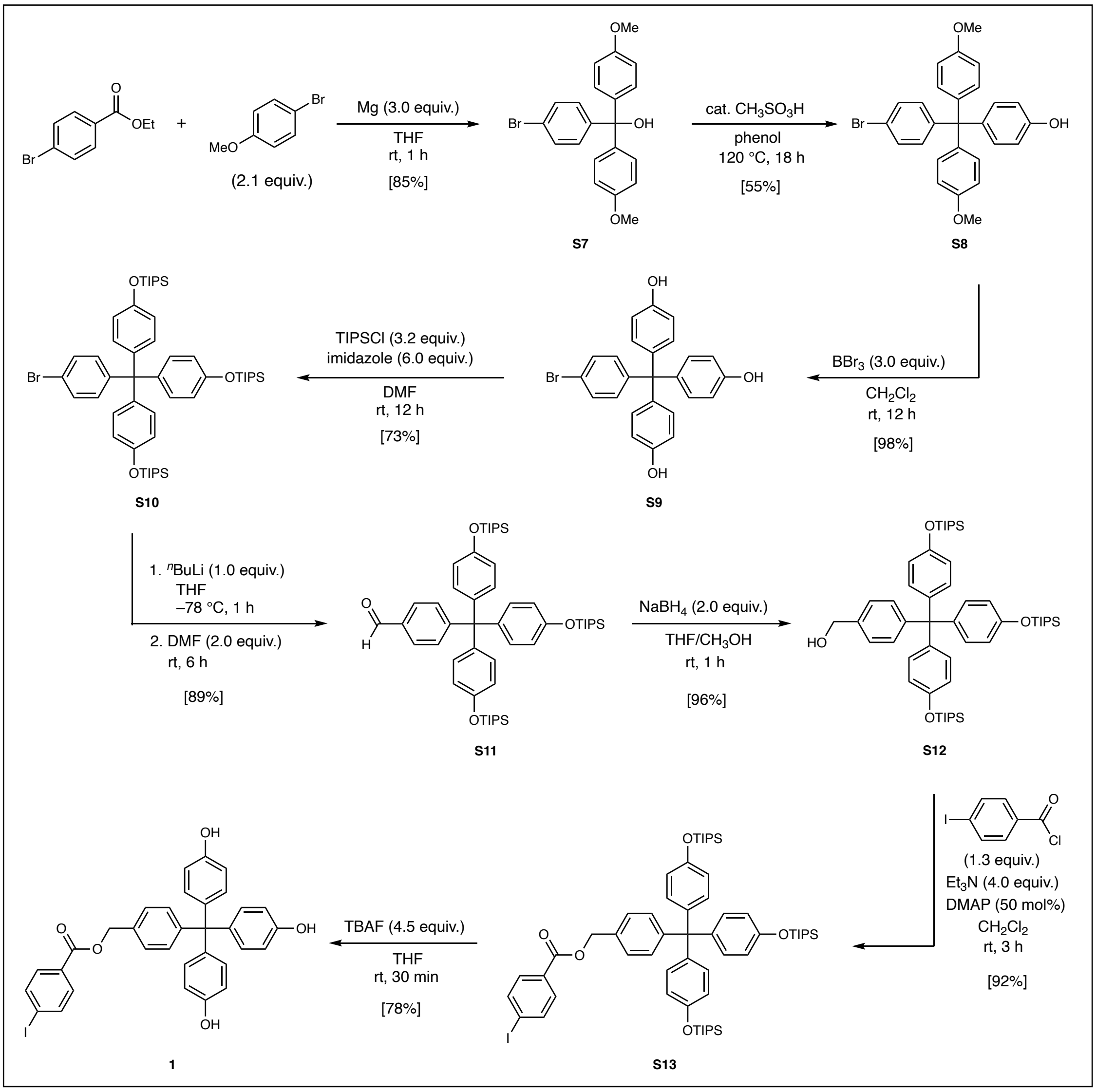

Note: **S7** and **S8** were synthesized based on the procedures reported in the literature[6].

(4-bromophenyl)bis(4-methoxyphenyl)methanol (S7)

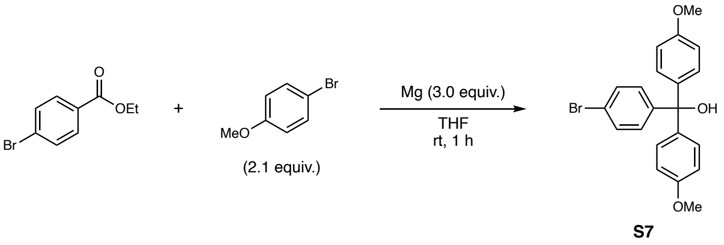

A 100-mL two-necked flask containing Mg powder (729 mg, 30 mmol) was heated by heat gun under vacuum for several minutes. The flask was filled with nitrogen followed by addition of

THF (40 mL). 4-Bromoanisole (3.93 g, 21 mmol) was slowly added to the solution with great care of the temperature. The reaction mixture was stirred for 1 h without heating. Ethyl 4-bromobenzoate (1.66 g, 10 mmol) was then added to the flask at 0 °C. The reaction mixture was stirred for 1 h at room temperature. The reaction was quenched by addition of sat. NH_4Cl aq. and aqueous layer was extracted with Et_2O for three times. Combined organic layer was washed with brine, dried over Na_2SO_4 and concentrated under reduced pressure. Crude product was purified by silica gel column chromatography (eluent: hexane/AcOEt = 7:1 → 4:1) to afford **S7** (4.04 g, 85%) as a colorless viscose oil. ^{1}H NMR spectrum of **S7** agrees with reported data[6].

4-((4-bromophenyl)bis(4-methoxyphenyl)methyl)phenol (S8)

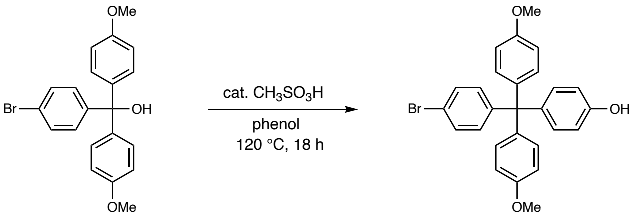

S7 **S8**

To a 100-mL round-bottom flask were added **S7** (4.04 g, 8.5 mmol) and phenol (7.0 mL). CH_3SO_3H (0.05 mL) was added to the flask with vigorous stirring. The reaction mixture was stirred for 18 h at 120 °C. H_2O was added to the reaction mixture and aqueous layer was extracted with AcOEt for three times. Combined organic layer was washed with brine, dried over Na_2SO_4 and concentrated under reduced pressure. Crude product was purified by silica gel column chromatography (eluent: hexane/AcOEt = 7:1 → 4:1) to afford **S8** (2.25 g, 55%) as a colorless viscose oil. ^{1}H NMR spectrum of **S8** agrees with reported data[6].

4,4',4''-((4-bromophenyl)methanetriyl)triphenol (S9)

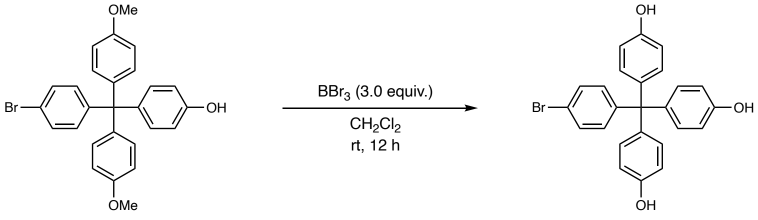

S8 **S9**

To a flame dried 200-mL two-necked flask was added **S8** (2.25 g, 4.7 mmol). The flask was degassed followed by addition of CH_2Cl_2 (94 mL). After cooling to –78 °C, BBr_3 (ca. 1.0 M solution in CH_2Cl_2, 14.2 mL, 14.2 mmol) was slowly added to the flask. Then the reaction mixture was warmed up to room temperature and stirred for 12 h at room temperature. Water was then

added to the flask to quench the reaction. Aqueous layer was extracted with AcOEt three times, dried over Na_2SO_4 and then concentrated *in vacuo*. Crude product was purified by silica gel column chromatography (eluent: hexane/AcOEt = 60:40 → 50:50) to afford **S9** (2.08 g, 98%) as a reddish solid.

^{1}H NMR (600 MHz, DMSO-d_6) δ 9.34 (s, 3H), 7.45 (d, J = 8.6 Hz, 2H), 7.00 (d, J = 8.6 Hz, 2H), 6.83 (d, J = 8.6 Hz, 6H), 6.65 (d, J = 8.6 Hz, 6H).

^{13}C NMR (150 MHz, DMSO-d_6) δ155.20, 147.34, 137.07, 132.61, 131.40, 130.24, 118.92, 114.28, 61.96.

HRMS (APCI$^-$): calcd. for $C_{25}H_{18}BrO_3$ ([M–H]$^-$): 445.0445, found: 445.0434 (error: 2.47 ppm).

((((4-bromophenyl)methanetriyl)tris(benzene-4,1-diyl))tris(oxy))tris(triisopropylsilane) (S10)

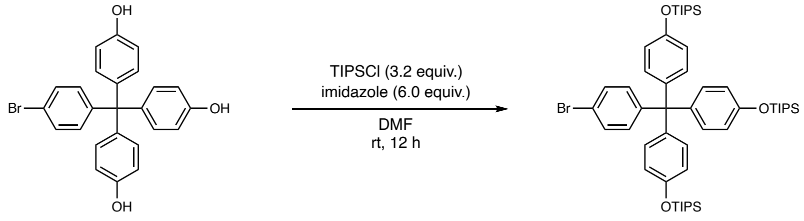

S9 S10

To a 100-mL flask were added **S9** (2.13 g, 4.76 mmol) and DMF (25.0 mL). After cooling to 0 °C, triisopropylsilyl chloride (TIPSCl) (3.20 mL, 15.2 mmol) and imidazole (1.96 g, 28.8 mmol) were then added to the flask. The reaction mixture was stirred at room temperature for 12 h. NH_4Cl aq. was added and the mixture was extracted with hexane/$CHCl_3$ = 4:1 three times, washed with H_2O and brine. Combined organic layer was dried over Na_2SO_4 and then concentrated *in vacuo*. The crude product was purified by silica gel column chromatography (eluent: hexane/$CHCl_3$ = 100:0 → 85:15) to afford **S10** (3.01 g, 73%) as a white solid.

^{1}H NMR (500 MHz, $CDCl_3$) δ 7.32 (d, J = 8.6 Hz, 2H), 7.01 (d, J = 8.9 Hz, 2H), 6.93 (d, J = 8.9 Hz, 6H), 6.73 (d, J = 8.9 Hz, 6H), 1.23 (sep, J = 7.3 Hz, 9H), 1.08 (d, J = 7.4 Hz, 54H).

^{13}C NMR (150 MHz, $CDCl_3$) δ 154.17, 147.05, 139.49, 132.97, 132.10, 130.38, 119.92, 118.87, 62.83, 18.03, 12.74.

HRMS (FAB$^+$): calcd. for $C_{52}H_{79}BrO_3Si_3$ ([M]$^+$): 914.4520, found: 914.4511 (error: 0.98 ppm).

4-(tris(4-((triisopropylsilyl)oxy)phenyl)methyl)benzaldehyde (S11)

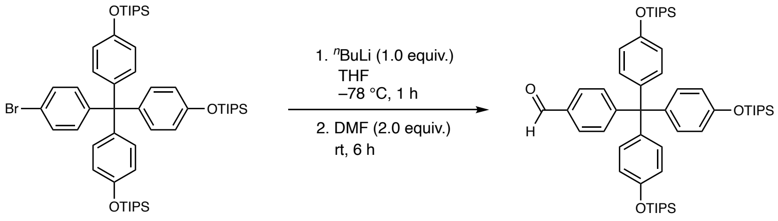

S10 **S11**

To a flame dried 200-mL two-necked flask was added **S10** (5.80 g, 6.30 mmol). The flask was degassed followed by addition of THF (63 mL). After cooling to –78 °C, *n*-BuLi (1.6 M solution in hexane, 3.95 mL, 6.30 mmol) was slowly added to the flask. The reaction mixture was stirred for 1 h at –78 °C. DMF (1.45 mL, 18.9 mmol) was slowly added at –78 °C and the reaction mixture was stirred at room temperature for 6 h. The reaction was quenched by addition of 1M HCl aq. at 0 °C. Aqueous layer was extracted with diethyl ether three times, dried over $MgSO_4$ and then concentrated *in vacuo*. Crude product was purified by silica gel column chromatography (eluent: hexane/$CHCl_3$ = 89:11 → 80:20) to afford **S11** (4.84 g, 89%) as a white solid.

^{1}H NMR (500 MHz, $CDCl_3$) δ 9.98 (s, 1H), 7.73 (d, *J* = 8.3 Hz, 2H), 7.34 (d, *J* = 8.3 Hz, 2H), 6.95 (d, *J* = 8.9 Hz, 6H), 6.75 (d, *J* = 8.6 Hz, 6H), 1.24 (sep, *J* = 7.2 Hz, 9H), 1.08 (d, *J* = 7.2 Hz, 54H).

^{13}C NMR (150 MHz, $CDCl_3$) δ 192.22, 155.18, 154.29, 139.15, 134.15, 132.10, 131.77, 128.84, 119.03, 63.56, 18.05, 12.75.

HRMS (APCI$^+$): calcd. for $C_{53}H_{81}O_4Si_3$ ([M+H]$^+$): 865.5437, found: 865.5442 (error: 0.58 ppm).

(4-(tris(4-((triisopropylsilyl)oxy)phenyl)methyl)phenyl)methanol (S12)

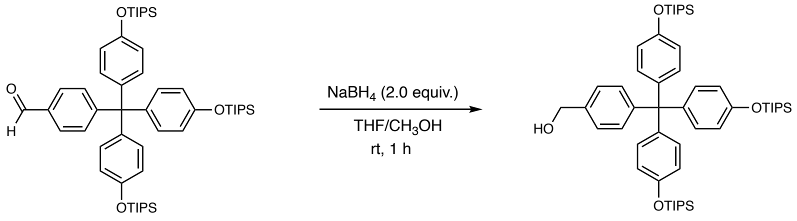

S11 **S12**

To a 100-mL flask were added **S11** (4.84 g, 5.60 mmol), THF (28.0 mL) and methanol (28 mL). $NaBH_4$ (424 mg, 11.2 mmol) was added to the solution in one portion. The reaction mixture was stirred at room temperature for 1 h. NH_4Cl aq. was added to quench the reaction and the aqueous layer was extracted with CH_2Cl_2 three times. Combined organic layer was dried over Na_2SO_4 and then concentrated *in vacuo* to afford **S12** (4.65 g, 96%) as a white solid. It is used for the next reaction without further purification.

^{1}H NMR (600 MHz, $CDCl_3$) δ 7.22 (d, *J* = 8.3 Hz, 2H), 7.14 (d, *J* = 8.6 Hz, 2H), 6.95 (d, *J* = 8.9

Hz, 6H), 6.73 (d, J = 8.9 Hz, 6H), 4.67 (s, 2H), 1.23 (sep, J = 7.2 Hz, 9H), 1.08 (d, J = 7.2 Hz, 54H).

^{13}C NMR (150 MHz, $CDCl_3$) δ 154.03, 147.48, 139.96, 138.25, 132.19, 131.46, 126.08, 118.78, 65.32, 63.00, 18.05, 12.76.

HRMS (FAB$^+$): calcd. for $C_{53}H_{82}O_4Si_3$ ([M]$^+$): 866.5521, found: 866.5533 (error: 1.38 ppm).

4-(tris(4-((triisopropylsilyl)oxy)phenyl)methyl)benzyl 4-iodobenzoate (S13)

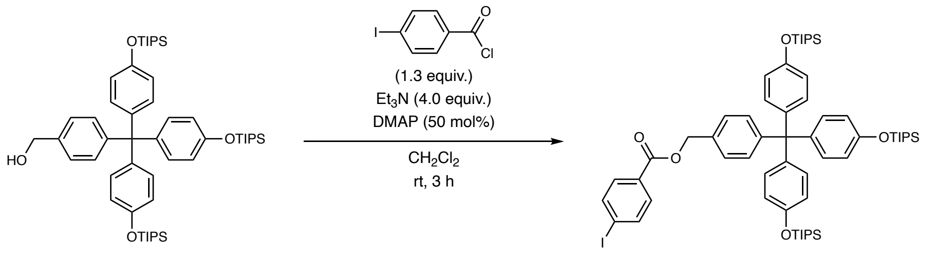

To a 100-mL flask were added **S12** (4.65 g, 5.36 mmol), 4-iodobenzoyl chloride (1.86 g, 6.97 mmol), 4-dimethylaminopyridine (DMAP) (327 mg, 2.68 mmol) and CH_2Cl_2 (54 mL). Et_3N (2.98 mL, 21.4 mmol) was then added to the solution. The reaction mixture was stirred at room temperature for 3 h. NH_4Cl aq. was added to quench the reaction and the aqueous layer was extracted with CH_2Cl_2 three times. Combined organic layer was dried over Na_2SO_4 and then concentrated *in vacuo*. The crude product was purified by silica gel column chromatography (eluent: hexane/AcOEt = 100:0 → 93:7) to afford **S13** (5.44 g, 92%) as a white solid.

^{1}H NMR (500 MHz, TCE-d_2) δ 7.83 (d, J = 8.6 Hz, 2H), 7.79 (d, J = 8.6 Hz, 2H), 7.29 (d, J = 8.6 Hz, 2H), 7.17 (d, J = 8.4 Hz, 2H), 6.98 (d, J = 8.6 Hz, 6H), 6.75 (d, J = 8.6 Hz, 6H), 5.34 (s, 2H), 1.23 (sep, J = 7.5 Hz, 9H), 1.08 (d, J = 7.5 Hz, 54H).

^{13}C NMR (150 MHz, $CDCl_3$) δ 166.15, 154.06, 148.16, 139.82, 137.87, 133.11, 132.17, 131.45, 131.31, 129.80, 127.21, 118.80, 100.98, 66.82, 63.04, 18.05, 12.75.

HRMS (FAB$^+$): calcd. for $C_{60}H_{85}IO_5Si_3$ ([M]$^+$): 1096.4749, found: 1096.4769 (error: 1.82 ppm).

4-(tris(4-hydroxyphenyl)methyl)benzyl 4-iodobenzoate (1)

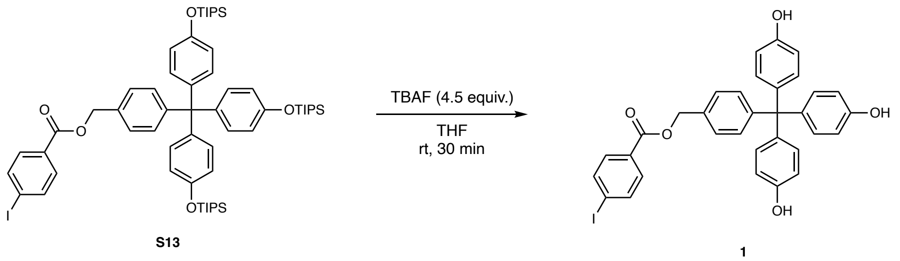

To a 100-mL flask were added **S13** (5.44 g, 4.96 mmol) and THF (100 mL). Tetrabutylammoniun fluoride (TBAF) (1 M solution in THF, 22.3 mL, 22.3 mmol) was then added dropwise to the solution. The reaction mixture was stirred at room temperature for 30 min. NH_4Cl aq. was added to quench the reaction and the aqueous layer was extracted with AcOEt three times. Combined organic layer was dried over Na_2SO_4 and then concentrated under reduced pressure. The crude product was purified by silica gel column chromatography (eluent: $CHCl_3$/THF = 87:13 → 50:50) followed by reprecipitation from diethyl ether to afford **1** (2.44 g, 78%) as a white solid.

^{1}H NMR (600 MHz, DMSO-d_6) δ 9.31 (s, 3H), 7.92 (d, J = 7.9 Hz, 2H), 7.74 (d, J = 8.3 Hz, 2H), 7.34 (d, J = 8.2 Hz, 2H), 7.10 (d, J = 8.2 Hz, 2H), 6.85 (d, J = 8.6 Hz, 6H), 6.64 (d, J = 8.6 Hz, 6H), 5.30 (s, 2H).

^{13}C NMR (150 MHz, DMSO-d_6) δ 165.32, 155.08, 147.89, 137.85, 137.45, 132.99, 131.45, 131.01, 130.56, 129.04, 127.03, 114.19, 102.03, 66.12, 62.14.

HRMS (APCI$^-$): calcd. for $C_{33}H_{24}IO_5$ ([M–H]$^-$): 627.0674, found: 627.0661 (error: 2.07 ppm).

2-4. Synthesis of Alkyne-terminated Dendrimer

4-(tris(4-((3,5-bis((3,4,5-tris(prop-2-yn-1-yloxy)benzyl)oxy)benzyl)oxy)phenyl)methyl)benzyl 4-iodobenzoate (3)

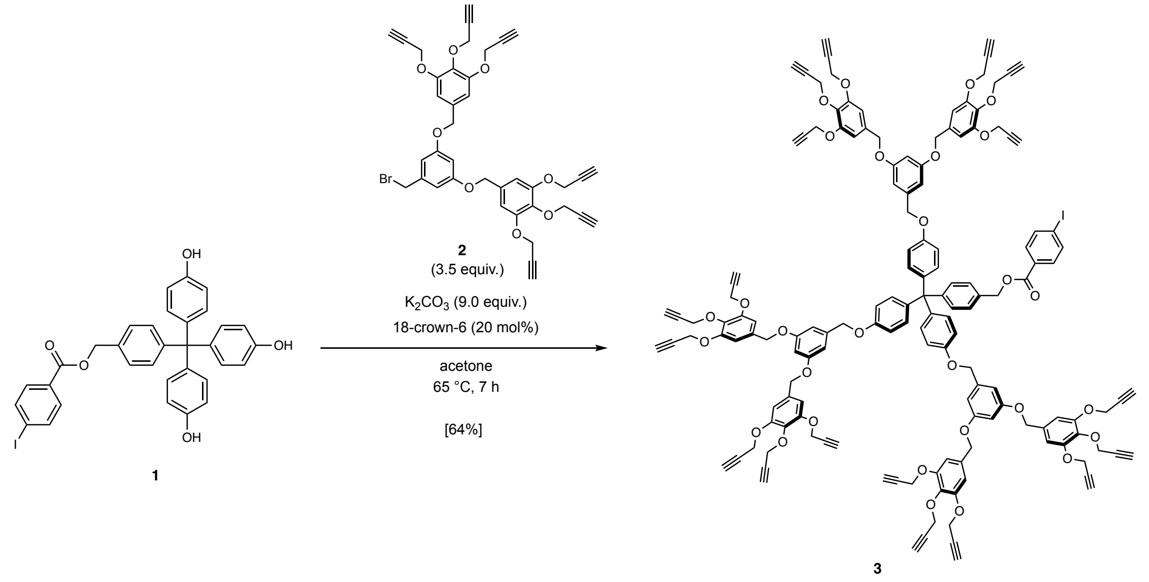

To a flame dried 50-mL two-necked flask were added **1** (628 mg, 1.0 mmol), **2** (2.47 g, 3.5 mmol), K_2CO_3 (1.25 g, 9.0 mmol) and 18-crown-6 (52.8 mg, 0.20 mmol). The flask was degassed followed by addition of acetone (10.0 mL). The reaction mixture was stirred for 7 h at 65 °C. Water was added to the reaction mixture and aqueous layer was extracted with diethyl ether/THF = 5:1 three times. Combined organic layer was washed with brine. After that organic layer was dried over $MgSO_4$ and then concentrated *in vacuo.* Crude product was purified by silica gel column chromatography (eluent: CH_2Cl_2/AcOEt = 100:0 → 95:5) to afford **3** (1.59 g, 64%) as a white solid.

^{1}H NMR (600 MHz, $CDCl_3$) δ 7.80 (d, *J* = 8.9 Hz, 2H), 7.77 (d, *J* = 8.6 Hz, 2H), 7.29 (d, *J* = 8.6 Hz, 2H), 7.20 (d, *J* = 8.6 Hz, 2H), 7.08 (d, *J* = 8.9 Hz, 6H), 6.86–6.80 (m, 18H), 6.67 (d, *J* = 2.0 Hz, 6H), 6.55 (t, *J* = 2.0 Hz, 3H), 5.32 (s, 2H), 4.98 (s, 12H), 4.95 (s, 6H), 4.75 (d, *J* = 2.0 Hz, 24H), 4.73 (d, *J* = 2.8 Hz, 12H), 2.47 (t, *J* = 2.4 Hz, 12H), 2.45 (t, *J* = 2.4 Hz, 6H).

^{13}C NMR (150 MHz, $CDCl_3$) δ 166.12, 160.10, 156.88, 151.90, 147.76, 139.75, 139.65, 137.91, 137.11, 133.44, 133.04, 132.21 (2C), 131.31, 129.75, 127.38, 113.76, 108.13, 106.85, 101.94, 101.04, 79.28, 78.51, 76.18, 75.44, 70.16, 70.08, 66.69, 63.00, 60.50, 57.26.

MS (MALDI$^+$): calcd. for $C_{150}H_{115}KIO_{29}$ ([M+K]$^+$): 2545.62, found: 2545.4. (Mode: linear mode, Matrix: *trans*-2-[3-(4-*tert*-butylphenyl)-2-methyl-2-propenylidene]malononitrile, DCTB)

2-5. Huisgen Cyclization Reaction of Dendrimer 3 with Alkyl Azide 4

Synthesis of $CuBr(PPh_3)_3$ for Huisgen cyclization reaction

$CuBr(PPh_3)_3$ was prepared according to the reported procedure[1].

A 500-mL round-bottom flask containing MeOH (170 mL) was heated to 65 °C. PPh_3 (9.98 g, 38 mmol) was added to the flask followed by portionwise addition of $CuBr_2$ (2.0 g, 9.0 mmol). The reaction mixture was stirred at 65 °C for 10 min. The reaction mixture was cooled to room temperature and the thus-generated white precipitate was collected by filtration. Filtrate cake was washed with EtOH and Et_2O and dried *in vacuo* to afford $CuBr(PPh_3)_3$ (7.64 g, 91%) as a white solid.

Synthesis of dendrimer support 5

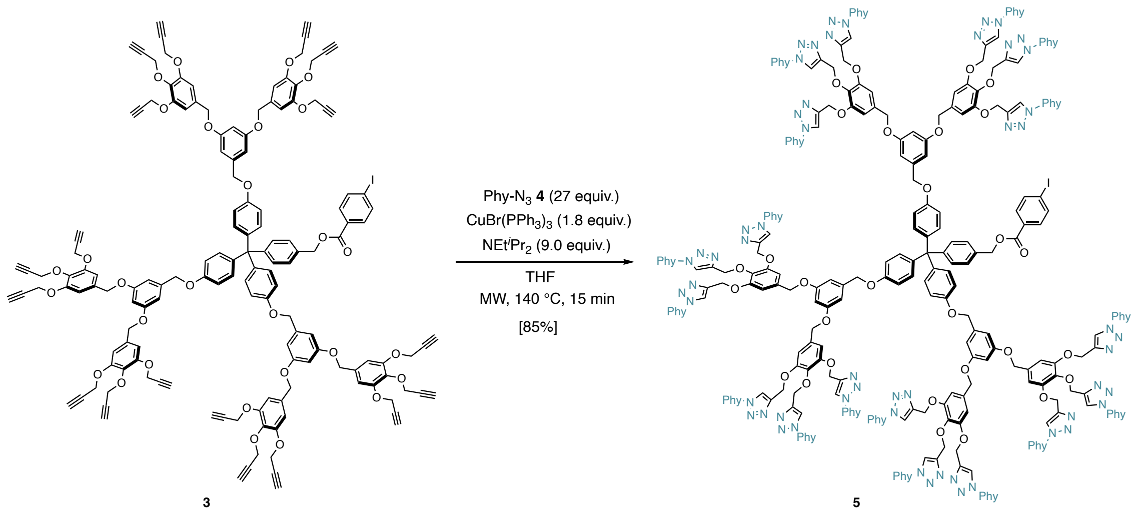

3 5

To a microwave vessel were added **3** (251 mg, 0.1 mmol, × 2 batches) and $CuBr(PPh_3)_3$ (167 mg, 0.18 mmol, × 2 batches). $PhyN_3$ **4** (875 mg, 2.7 mmol, × 2 batches) and NEt^iPr_2 (117 mg, 0.9 mmol, × 2 batches) dissolved in THF (3.0 mL, × 2 batches) were then added to the microwave vessel. The reaction mixture was stirred under microwave irradiation at 140 °C for 15 min. Reaction mixture was diluted by $CHCl_3$ and washed with NH_4Cl aq. three times. Organic layer was dried over Na_2SO_4 and then concentrated *in vacuo*. The crude product was purified by GPC to afford **5** (1.41 g, 85%, 2 batches total) as a sticky beige solid.

^{1}H NMR (600 MHz, $CDCl_3$) δ 7.88–7.71 (m, 22H), 7.29 (d, J = 8.4 Hz, 2H), 7.22 (d, J = 8.4 Hz, 2H), 7.14 (d, J = 9.0 Hz, 6H), 6.89 (d, J = 9.0 Hz, 6H), 6.83 (s, 12H), 6.73 (s, 6H), 6.58 (s, 3H), 5.31 (s, 2H), 5.22 (s, 24H), 5.16 (s, 12H), 5.00–4.91 (m, 18H), 4.42–4.25 (m, 36H), 1.98–1.86 (m, 18H), 1.77–1.63 (m, 18H), 1.53–0.97 (m, 396H), 0.95–0.76 (m, 270H).

^{13}C NMR (150 MHz, $CDCl_3$) δ 166.07, 160.12, 156.93, 152.58, 147.95, 144.56, 143.88, 139.73,

139.65, 137.85, 137.61, 133.27, 133.05, 132.22, 131.34, 131.29, 129.75, 127.34, 123.79, 123.13, 113.69, 107.38, 106.86, 101.60, 100.98, 70.15, 70.03, 66.71, 63.52, 63.02, 48.85, 48.66, 39.49, 37.65, 37.61, 37.58, 37.54, 37.52, 37.45, 37.41, 37.19, 37.16, 32.91, 30.65, 30.60, 28.09, 24.92, 24.61, 24.43, 24.38, 22.86, 22.76, 19.88, 19.86, 19.81, 19.40, 19.34.

MS (MALDI$^+$): calcd. for $C_{510}H_{853}NaIO_{29}N_{54}$ ([M+Na]$^+$): 8349.59, found: 8350.3. (Mode: linear mode, Matrix: DCTB)

3. Preparation of Monomer

3-1. Synthesis of Thiophene Monomer

8-(5-bromothiophen-2-yl)-4-methyldihydro-4λ^4,8λ^4-[1,3,2]oxazaborolo[2,3-*b*][1,3,2]oxazaborole-2,6(3*H*,5*H*)-dione (6)

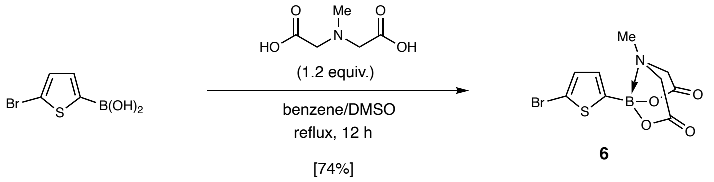

To a 200-mL round-bottom flask were added *N*-methyliminodiacetic acid (735 mg, 5.0 mmol), 5-bromo-2-thiopheneboronic acid (830 mg, 4.0 mmol), benzene (100 mL) and DMSO (10 mL). The reaction mixture was stirred with Dean–Stark apparatus for 12 h at 80 °C. Reaction mixture was concentrated under reduced pressure until all solvent removed. Crude product was purified by silica gel column chromatography (eluent: Et_2O/MeCN = 100:0 → 3:1) to afford **6** (940 mg, 74%) as a reddish solid. ^{1}H NMR spectrum of **6** agrees with reported data[7].

3-2. Synthesis of Benzene Monomer

8-(4-bromophenyl)-4-methyldihydro-4λ^4,8λ^4-[1,3,2]oxazaborolo[2,3-*b*][1,3,2]oxazaborole-2,6(3*H*,5*H*)-dione (9)

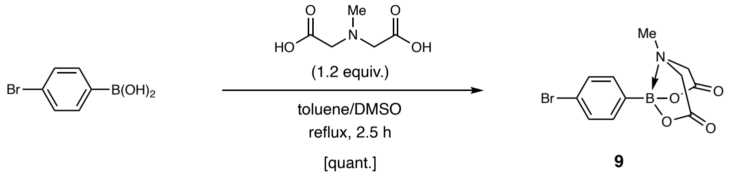

To a 1-L round-bottom flask were added *N*-methyliminodiacetic acid (1.76 g, 12.0 mmol), 4-bromophenylboronic acid (2.02 g, 10.1 mmol), toluene (300 mL) and DMSO 30 mL). The reaction mixture was stirred with Dean–Stark apparatus for 2.5 h at 120 °C. Reaction mixture was concentrated under reduced pressure until all solvent removed. Crude product was purified by silica gel column chromatography (eluent: Et_2O/MeCN = 3:1 → 1:1) to afford **9** (3.2 g, quant.) as a white solid. ^{1}H NMR spectrum of **9** agrees with reported data[7].

3-3. Synthesis of Fluorene Monomer

Note: 2,7-dibromo-9,9-dimethyl-9H-fluorene was synthesized according to the procedure reported in the literature[8].

2-(7-bromo-9,9-dimethyl-9*H*-fluoren-2-yl)-4,4,5,5-tetramethyl-1,3,2-dioxaborolane (12)

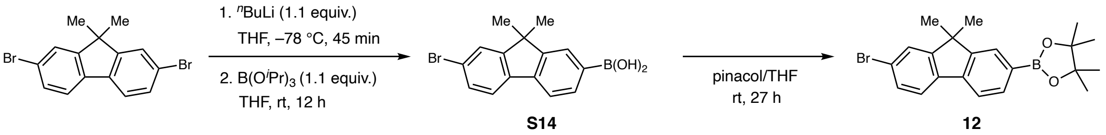

To a flame dried 100-mL two-necked flask was added 2,7-dibromo-9,9-dimethyl-9H-fluorene (4.0 g, 7.3 mmol). The flask was degassed followed by addition of THF (50 mL). After cooling to –78 °C, *n*-BuLi (1.6 M solution in hexane, 4.8 mL, 8.0 mmol) was slowly added to the flask. The reaction mixture was stirred for 45 min at –78 °C. $B(O^iPr)_3$ (1.4 mL, 8.0 mmol) was added in one portion at –78 °C and the reaction mixture was stirred at room temperature for 12 h. The reaction was quenched by addition of water. Aqueous layer was extracted with AcOEt three times, dried over Na_2SO_4 and then concentrated *in vacuo* to afford white solid. The crude product of **S14** was used for the next reaction without further purification.

To a 200-mL round-bottom flask were added crude boronic acid, THF (20 mL) and pinacol (8.0 mL). The reaction mixture was stirred at room temperature for 27 h. Water was added to the reaction mixture and extracted with diethyl ether three times. Combined organic layer was washed with brine, dried over $MgSO_4$ and then concentrated *in vacuo*. Crude product was purified by silica gel column chromatography (eluent: hexane/AcOEt = 100:0 → 95:5) to afford **12** (1.87 g, 64%; 2 steps) as a white solid.

^{1}H NMR (500 MHz, $CDCl_3$) δ 7.86 (s, 1H), 7.81 (dd, *J* = 7.6, 0.9 Hz, 1H), 7.69 (d, *J* = 8.4 Hz, 1H), 7.60 (d, *J* = 8.1 Hz, 1H), 7.56 (d, *J* = 1.8 Hz, 1H), 7.46 (dd, *J* = 8.2, 1.7 Hz, 1H), 1.49 (s, 6H), 1.38 (s, 12H).

^{13}C NMR (150 MHz, $CDCl_3$) δ 156.49, 152.54, 141.22, 138.14, 134.21, 130.25, 128.95, 126.37, 121.96, 121.75, 119.54, 83.99, 47.30, 27.08, 25.05.

(Note: One aromatic carbon atom next to boron atom was not observed due to quadrupolar coupling.)

^{11}B NMR (192 MHz, $CDCl_3$) δ 29.9.

HRMS (FAB$^+$): calcd. for $C_{21}H_{24}BBrO_2$ ($[M]^+$): 398.1053, found: 398.1061 (error: 2.01 ppm).

3-4. Synthesis of Benzotriazole Monomer 15

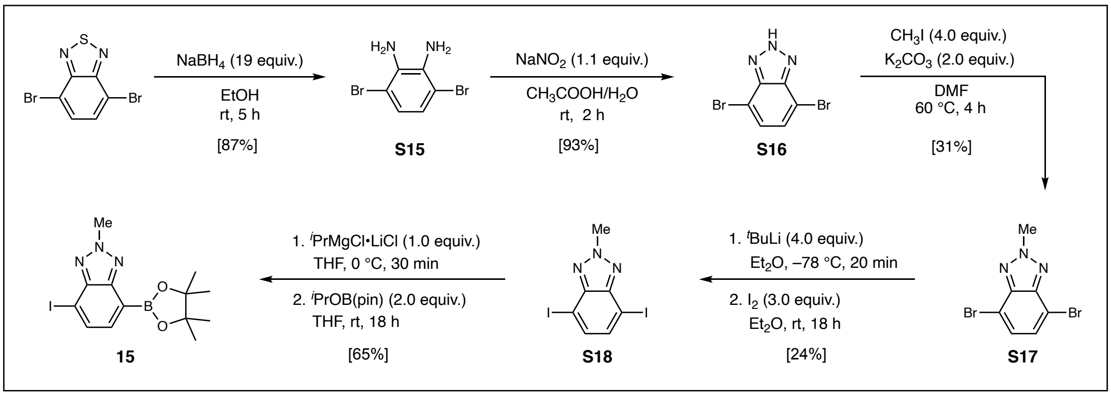

Note: **S15** and **S16** were synthesized based on the procedures reported in the literature[9, 10].

3,6-dibromobenzene-1,2-diamine (S15)

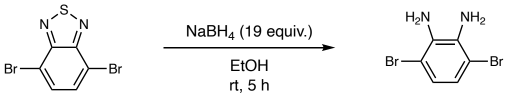

S15

To a 500-mL round-bottom flask were added dibromobenzothiadiazole (5.89 g, 20 mmol) and EtOH (200 mL). $NaBH_4$ (14.4 g, 380 g) was added portionwise at 0 °C. The reaction mixture was stirred at 0 °C for 10 min and then stirred at room temperature for additional 5 h. The reaction was quenched by addition of H_2O at 0 °C. EtOH was removed by evaporation and residual aqueous solution was extracted by AcOEt for three times. Combined organic layer was washed with brine, dried over Na_2SO_4 and concentrated under reduced pressure to afford **S15** (4.65 g, 87%) as a off-white solid. ^{1}H NMR spectrum of **S15** agrees with reported data[9].

4,7-dibromo-2H-benzo[d][1,2,3]triazole (S16)

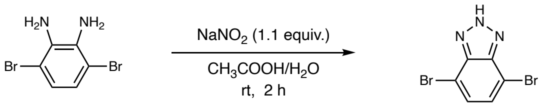

S15 **S16**

A 200-mL round-bottom flask containing AcOH (35 mL) was cooled to 0 °C. **S15** (4.65 g, 17.5 mmol) was added to the flask at 0 °C followed by dropwise addition of a solution of $NaNO_2$ (1.33 g, 19.3 mmol) in 35 mL H_2O at room temperature. The reaction mixture was stirred for 2 h at room temperature. The reaction mixture was cooled to 0 °C and precipitated was collected by filtration. Brown precipitate was washed with H_2O on the filter paper and dried *in vacuo* to afford **S16** (4.49 g, 93%) as a brown solid. ^{1}H NMR spectrum of **S16** agrees with reported data[10].

4,7-dibromo-2-methyl-2*H*-benzo[*d*][1,2,3]triazole (S17)

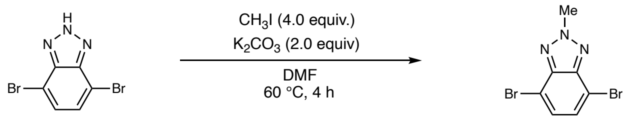

S16 **S17**

To a 100-mL two-necked flask were added **S16** (1.39 g, 5.0 mmol) and K_2CO_3 (1.38 g, 10 mmol). The flask was degassed followed by addition of DMF (50 mL). The reaction mixture was stirred at 60 °C for 30 min. Iodomethane (2.84 g, 20 mmol) was then added to a reaction mixture in one portion. The reaction mixture was stirred at 60 °C for 4 h. After cooling to the room temperature, water was added and extracted with mixed solvent of hexane/AcOEt = 4:1. Combined organic layer was washed with brine, dried over Na_2SO_4 and then concentrated in *vacuo.* Crude product was purified by silica gel column chromatography (eluent: hexane/AcOEt = 91:9) to afford **S17** (451.1 mg, 31%) as a white solid.

^{1}H NMR (600 MHz, $CDCl_3$) δ 7.46 (s, 2H), 4.59 (s, 3H).

^{13}C NMR (150 MHz, $CDCl_3$) δ 144.11, 129.85, 109.98, 44.08.

HRMS (EI^+): calcd. for $C_7H_5Br_2N_3$ ($[M]^+$): 288.8850, found: 288.8856 (error: 2.08 ppm).

4,7-diiodo-2-methyl-2*H*-benzo[*d*][1,2,3]triazole (S18)

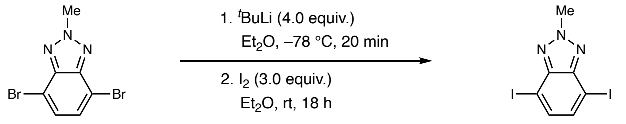

S17 **S18**

To a flame dried 200-mL two-necked flask was added **S17** (1.63 g, 5.6 mmol). The flask was degassed followed by addition of Et_2O (80 mL). The solution was cooled to –78 °C and then *t*-BuLi (1.52 M in pentane, 14.8 mL, 22.4 mmol) was added dropwise to the solution. The reaction mixture was stirred at –78 °C for 20 min. Then warmed up to the room temperature and I_2 (solid, 4.25 g, 16.8 mmol) was carefully added to the reaction mixture under N_2 flow. The reaction mixture was stirred at room temperature for 18 h. The reaction was quenched by addition of ^{i}PrOH at 0 °C and it was extracted with AcOEt three times. Combined organic layer was washed with brine, dried over and concentrated under reduced pressure. Crude product was purified by silica gel column chromatography (eluent: hexane/AcOEt = 91:9 → 87:13) to afford **S18** (511 mg, 24%) as a white solid.

^{1}H NMR (600 MHz, $CDCl_3$) δ 7.55 (s, 2H), 4.57 (s, 3H).

^{13}C NMR (150 MHz, $CDCl_3$) δ 145.38, 136.99, 82.19, 44.07.

HRMS (ESI$^+$): calcd. for $C_7H_6I_2N_3$ ([M+H]$^+$): 385.8646, found: 385.8651 (error: 1.30 ppm).

4-iodo-2-methyl-7-(4,4,5,5-tetramethyl-1,3,2-dioxaborolan-2-yl)-2*H*-benzo[*d*][1,2,3]triazole (15)

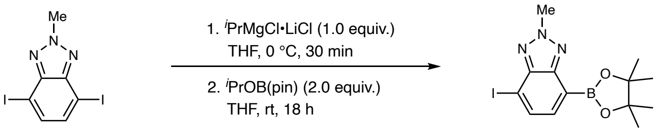

To a flame dried 50-mL two-necked flask was added **S18** (385 mg, 1.0 mmol). The flask was degassed followed by addition of THF (20 mL). The solution was cooled to 0 °C and then ^{i}PrMgCl•LiCl (1.35 M in THF, 0.75 mL, 1.0 mmol) was added dropwise to the solution. The reaction mixture was stirred at 0 °C for 30 min. ^{i}PrOB(pin) (0.41 mL, 2.0 mmol) was added in one portion and then warmed up to room temperature. The reaction mixture was stirred at room temperature for 18 h. 1M HCl aq. was added to the solution to quench the reaction and it was extracted with AcOEt three times. Combined organic layer was washed with brine, dried over Na_2SO_4 and concentrated under reduced pressure. Crude product was purified by GPC to afford **15** (251 mg, 65%) as a white solid.

^{1}H NMR (600 MHz, $CDCl_3$) δ 7.83 (d, J = 7.2 Hz, 1H), 7.62 (d, J = 7.2 Hz, 1H), 4.58 (s, 3H), 1.42 (s, 12H).

^{13}C NMR (150 MHz, $CDCl_3$) δ 146.46, 145.73, 136.26, 135.71, 86.36, 84.46, 44.01, 25.03. (Note: One aromatic carbon atom next to boron atom was not observed due to quadrupolar coupling.)

^{11}B NMR (192 MHz, $CDCl_3$) δ 29.6.

HRMS (ESI$^+$): calcd. for $C_{13}H_{17}BIN_3NaO_2$ ([M+Na]$^+$): 408.0351, found: 408.0351.

4. Polymerization Reaction on Dendrimer Support

4-1. Synthesis of Dendrimer-Ligated Polythiophene

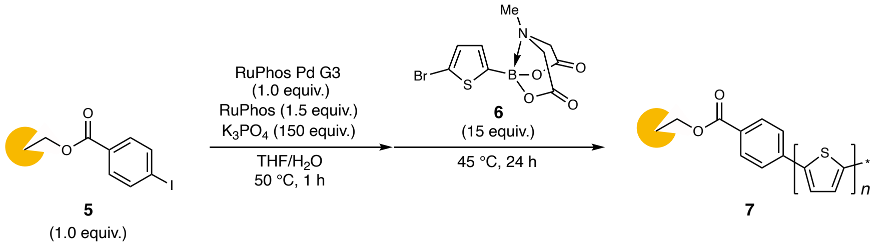

Oxidative addition complex formation: To a screw capped tube was added K_3PO_4 (257.9 mg, 1.2 mmol) and dried-up under vacuum. Dendrimer support **5** (66.7 mg, 8.0 μmol), RuPhos Pd G3 (6.7 mg, 8.0 μmol) and RuPhos (5.6 mg, 12.0 μmol) were added to the screw capped tube. The tube was filled with nitrogen followed by addition of THF (2.0 mL) and H_2O (0.72 mL). The reaction mixture was stirred at 50 °C for 1 h. The color of thus-obtained solution was clear yellow.

Polymerization reaction: To a 50-mL two-necked flask was added thiophene monomer **6** (38.1 mg, 120 μmol). The flask was filled with nitrogen and dry THF (18.0 mL) was added. Pre-prepared catalyst solution was added in one portion to the flask by syringe. 2.0 mL of THF was used for washing the vessel. The reaction mixture was stirred at 45 °C for 24 h. The reaction was quenched by addition of 1M HCl aq. at room temperature. The mixture was extracted with $CHCl_3$ (ca. 50 mL × 3) and combined organic layers were dried over Na_2SO_4. It was filtrated by membrane filter (pore size: 0.2 μm) and filtrate was concentrated under reduced pressure. Resulting crude product was reprecipitated from acetone to afford **7** (74.2 mg) as a red solid.

4-2. Synthesis of Dendrimer-Ligated Poly(*para*-phenylene)

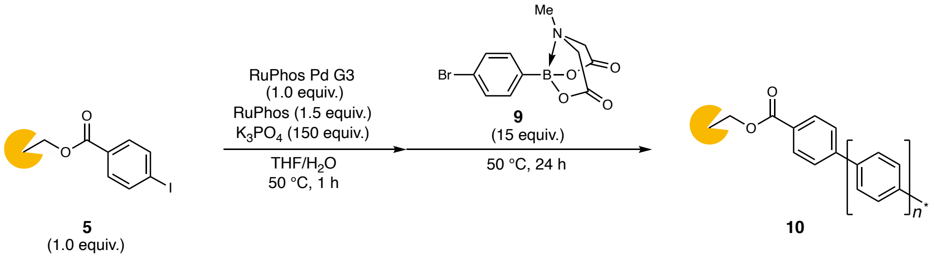

Oxidative addition complex was synthesized in the same procedure as for polythiophene.

Polymerization reaction: To a 50-mL two-necked flask was added benzene monomer **9** (38.2 mg, 120 μmol). The flask was filled with nitrogen and dry THF (18.0 mL) was added. Pre-prepared catalyst solution was added to the flask in one portion by syringe. 2.0 mL of THF was used for washing the vessel. The reaction mixture was stirred at 50 °C for 24 h. The reaction was quenched by addition of 1M HCl aq. at room temperature. The mixture was extracted with $CHCl_3$ (ca. 50

mL × 3) and the combined organic layers were dried over Na_2SO_4. It was filtrated by membrane filter (pore size: 0.2 μm) and filtrate was concentrated under reduced pressure. Resulting crude product was reprecipitated from acetone to afford **10** (57.4 mg) as an off-white solid.

4-3. Synthesis of Dendrimer-Ligated Polyfluorene

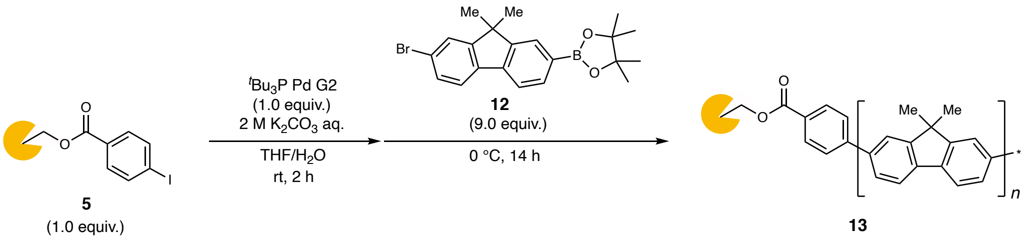

Oxidative addition complex formation: To a screw capped tube were added dendrimer support **5** (66.8 mg, 8 μmol) and ^{t}Bu$_3$P Pd G2 (4.1 mg, 8.0 μmol). The tube was filled with nitrogen followed by addition of THF (1.0 mL) and 2 M K_2CO_3 aq. (0.35 mL). The reaction mixture was stirred at room temperature for 2 h. The color of thus-obtained solution was clear yellow.

Polymerization reaction: To a 50-mL two-necked flask was added fluorene monomer **12** (28.7 mg, 72 μmol). The flask was filled with nitrogen and dry THF (13.0 mL) was added. Pre-prepared catalyst solution was added to the flask in one portion by syringe. 2.0 mL of THF was used for washing the vessel. The reaction mixture was stirred at 0 °C for 14 h. The reaction was quenched by addition of 1M HCl aq. at room temperature. The mixture was extracted with $CHCl_3$ (ca. 60 mL × 3) and the combined organic layers were dried over Na_2SO_4. It was filtrated by membrane filter (pore size: 0.2 μm) and filtrate was concentrated under reduced pressure. Resulting crude product was reprecipitated from acetone to afford **13** (50.0 mg) as an off-white solid.

4-4. Synthesis of Dendrimer-Ligated Polybenzotriazole

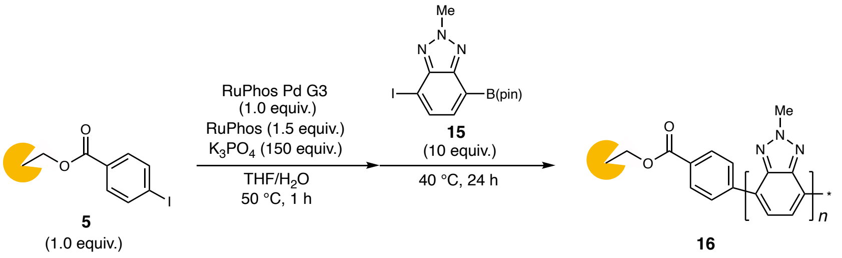

Oxidative addition complex was synthesized in the same procedure as for polythiophene.

Polymerization reaction: To a 50-mL two-necked flask was added benzotriazole monomer **15** (30.9 mg, 80 μmol). The flask was filled with nitrogen and dry THF (18.0 mL) was added. Pre-prepared catalyst solution was added to the flask in one portion by syringe. 2.0 mL of THF was

used for washing the vessel. The reaction mixture was stirred at 40 °C for 24 h. The reaction was quenched by addition of 1M HCl aq. at room temperature. The mixture was extracted with $CHCl_3$ (ca. 50 mL × 3) and the combined organic layers were dried over Na_2SO_4. It was filtrated by membrane filter (pore size: 0.2 μm) and filtrate was concentrated under reduced pressure. Resulting crude product was reprecipitated from acetone to afford **16** (63.4 mg) as orange solid.

4-5. Synthesis of Dendrimer-Ligated Poly(*para*-phenylene)-*block*-polythiophene

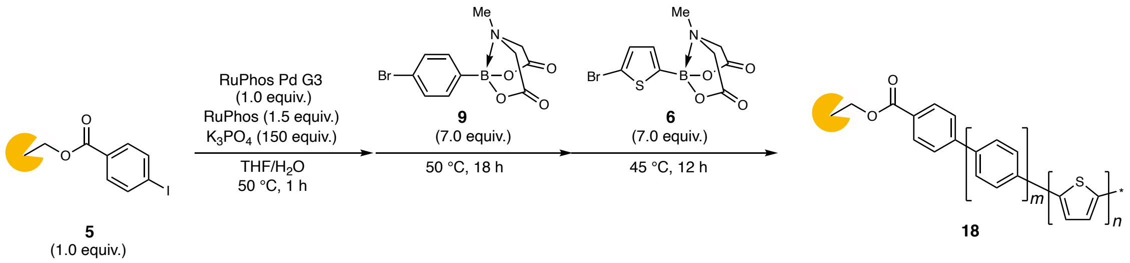

Oxidative addition complex was synthesized in the same procedure as for polythiophene.

Polymerization reaction: To a 50-mL two-necked was added benzene monomer **9** (17.5 mg, 56 μmol). The flask was filled with nitrogen and dry THF (18.0 mL) was added. Polymerization reaction was initiated by addition of pre-prepared catalyst solution in ono portion by syringe. 2.0 mL of THF was used for washing the vessel. The reaction mixture was stirred at 50 °C for 18 h. Resulting solution was cooled to room temperature and thiophene monomer **6** (17.8 mg, 56 μmol) dissolved in 3.0 mL THF was added. The reaction mixture was stirred at 45 °C for additional 12 h. It was quenched by addition of 1 M HCl aq. at room temperature. The mixture was extracted with $CHCl_3$ (ca. 50 mL × 3) and the combined organic layers were dried over Na_2SO_4. It was filtrated by membrane filter (pore size: 0.2 μm) and filtrate was concentrated under reduced pressure. Resulting crude product was reprecipitated from acetone to afford **18** (68.1 mg) as red solid.

5. Cleavage Reaction between Dendrimer Support and Polyarylene

5-1. General Procedure for Cleavage Reaction

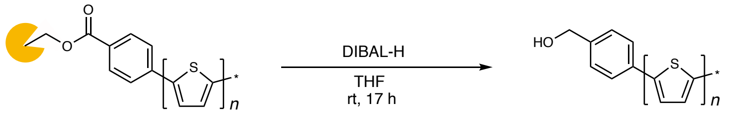

To a flame dried 50-mL two-necked flask was added dendrimer-ligated polyarylene **7** (87.5 mg). The flask was filled with nitrogen followed by addition of THF (5 mL). The solution was then cooled to 0 °C and diisobutylaluminium hydride (DIBAL-H) (1 M in hexane, 1.5 mL, 1.5 mmol) was added dropwise at 0 °C. The reaction mixture was warmed up to room temperature and stirred for 17 h. The reaction was quenched by slow addition of saturated Rochelle salt aq. at 0 °C. The solution was stirred at room temperature for several hours to achieve complete quenching. Aqueous layer was removed by pipette and organic layer (containing insoluble polyarylenes) was transferred to centrifuge tube. Centrifugation (5000 rpm, 30 min) and removing the supernatant by decantation afforded red precipitate in the tube. The solid was washed by THF, 1 M HCl aq. and acetone by centrifugation and decantation. Finally, red precipitate was filtrated by membrane filter (pore size: 0.2 μm) and carefully washed with $CHCl_3$, THF, H_2O and acetone to afford insoluble polythiophene **8** (7.5 mg, 70%, 2 steps) as a red insoluble solid.

5-2. Synthesis of Unsubstituted Poly(*para*-phenylene)

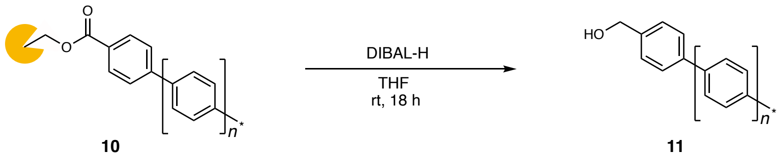

Unsubstituted poly(*para*-phenylene) **11** was synthesized according to general procedure for cleavage reaction. 5.5 mg, 56% (2 steps), white insoluble solid.

5-3. Synthesis of Unsubstituted Polyfluorene

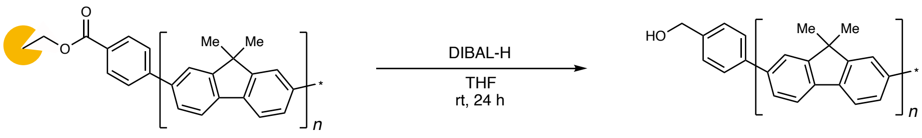

Unsubstituted polybenzotriazole **14** was synthesized according to general procedure for cleavage reaction. 2.8 mg, 19% (2 steps), off-white solid.

5-4. Synthesis of Unsubstituted Polybenzotriazole

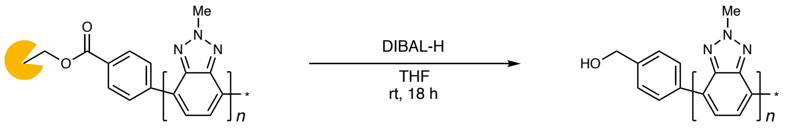

16 **17**

Unsubstituted polybenzotriazole **17** was synthesized according to general procedure for cleavage reaction. 8.9 mg, 78% (2 steps), orange insoluble solid.

5-5. Synthesis of Unsubstituted Poly(*para*-phenylene)-*block*-polythiophene

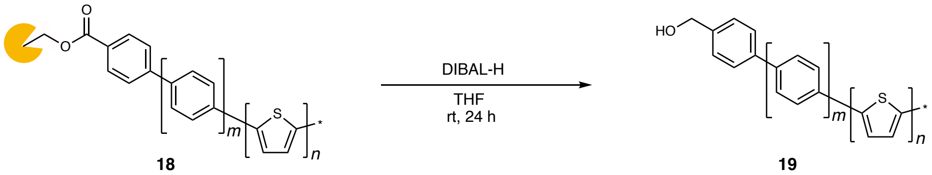

18 **19**

Unsubstituted poly(*para*-phenylene)-*block*-polythiophene **19** was synthesized according to general procedure for cleavage reaction. 6.8 mg, 70% (2 steps), red solid.

6. Control Experiment of Polymerization

6-1. ^{31}P NMR Study of Initiator Formation

In order to confirm the successful formation of initiator complex, oxidative addition step of dendrimer with RuPhos Pd G3 was monitored by ^{31}P NMR spectroscopy. All measurement was conducted in THF and locked by D_2O. RuPhos and RuPhos Pd G3 show their ^{31}P NMR signals at –8.0 ppm and 43.6 ppm, respectively. (Supplementary Fig. 1a, b). It is known that active palladium species was released from RuPhos Pd G3 under basic condition[11]. When RuPhos Pd G3 was stirred with 150 equiv. K_3PO_4 for 1 h at 50 °C, a signal appeared at 49.2 ppm (Supplementary Fig. 1c). It presumably corresponds to active palladium species. Next, the same process was conducted in the presence of 1.0 equiv. dendrimer support and 1.5 equiv. RuPhos. ^{31}P NMR spectrum shows a new signal at 34.3 ppm. (Supplementary Fig. 1d)

When methyl 4-bromobenzoate was used instead of dendrimer, the signal was also observed at 34.1 ppm (Supplementary Fig. 1e). Since isolation attempt of the oxidative addition complex was unsuccessful, oxidative addition complex of RuPhos-Pd to methyl 4-iodobenzoate **S19** was synthesized by method reported by Ito[12]. Oxidative addition complex **S19** was fully characterized by ^{1}H, ^{13}C NMR spectra and ESI MS, and ^{31}P NMR spectrum of **S19** shows signals at 23.3 ppm in THF. Based on these results, we concluded that the signal around 34 ppm (shown in Supplementary Fig. 1e) is not assigned to ones of **S19**. However, mass spectroscopy of obtained unsubstituted aromatic polymer corresponds to reaction monomer/initiator ratio (Figure 3b), which shows that polymerization-active oxidative addition complex (but different species from **S19**) is generated.

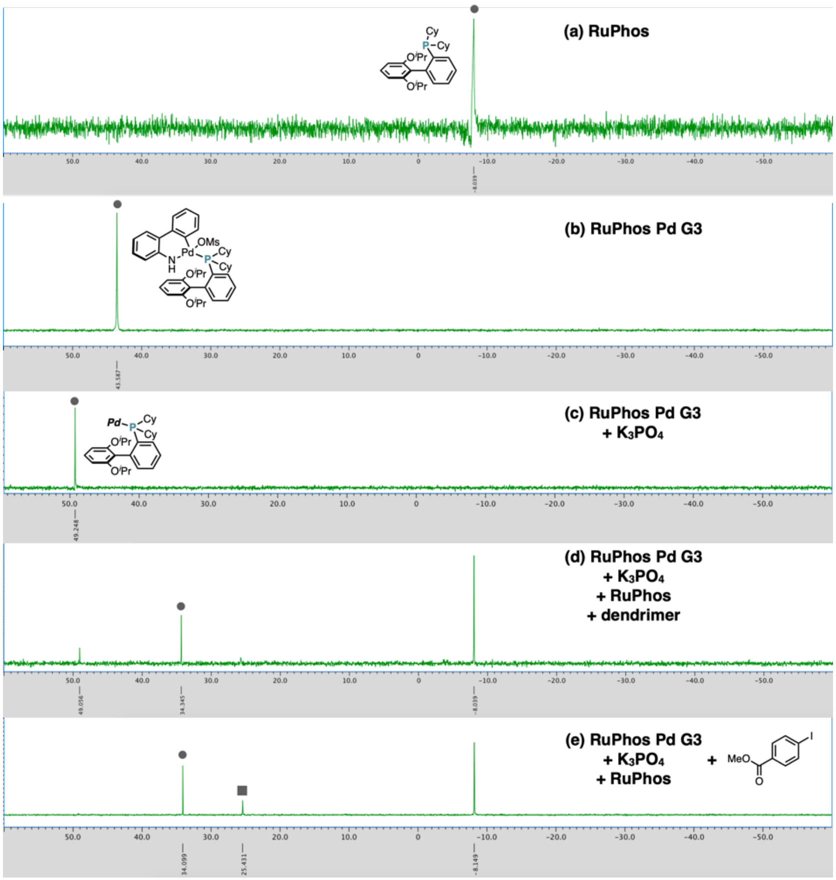

Supplementary Fig. 1. ^{31}P NMR spectra of (a) RuPhos, (b) RuPhos Pd G3, (c) active palladium species derives from RuPhos Pd G3, (d) oxidative addition complex to dendritic aryl iodide and (e) oxidative addition complex to methyl 4-iodobenzoate.

Oxidative addition complex formation of RuPhos Pd G3 with dendrimer for ^{31}P NMR study

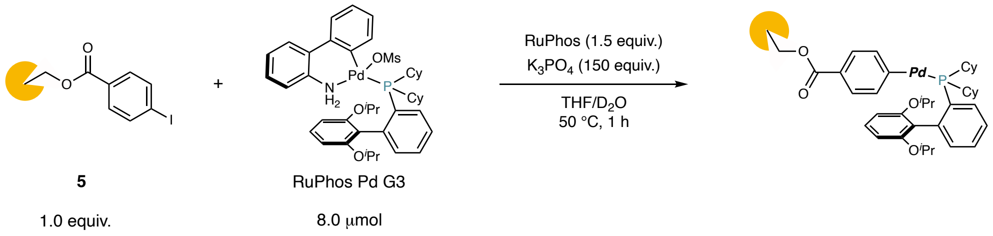

To a screw capped tube was added K_3PO_4 (257.8 mg, 1.2 mmol) and dried-up under vacuum. Dendrimer support **5** (66.7 mg, 8.0 μmol), RuPhos Pd G3 (6.7 mg, 8.0 μmol) and RuPhos (5.6 mg, 12 μmol) were added to the screw capped tube. The tube was filled with nitrogen followed by addition of THF (2.0 mL) and D_2O (0.72 mL). The reaction mixture was stirred at 50 °C for 1 h. The color of the solution turned to clear yellow. Organic layer of the reaction mixture was

transferred to the NMR tube under inert atmosphere and ^{31}P NMR was measured immediately (locked by residual D_2O signal).

Oxidative addition complex formation of RuPhos Pd G3 with methyl 4-iosobenzoate

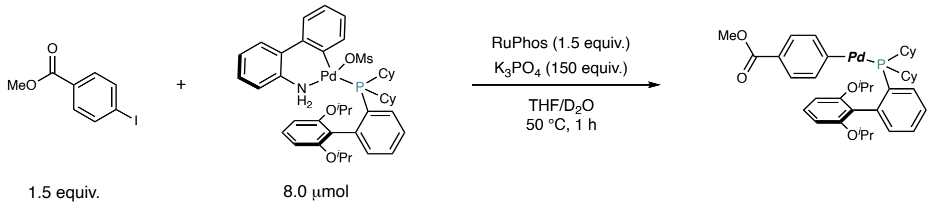

To a screw capped tube was added K_3PO_4 (256.9 mg, 1.2 mmol) and dried-up under vacuum. Methyl 4-iodobenzoate (3.5 mg, 12 μmol), RuPhos Pd G3 (6.7 mg, 8.0 μmol) and RuPhos (5.6 mg, 12 μmol) were added to the screw capped tube. The tube was filled with nitrogen followed by addition of THF (2.0 mL) and D_2O (0.72 mL). The reaction mixture was stirred at 50 °C for 1 h. The color of the solution turned to clear yellow. Organic layer of the reaction mixture was transferred to the NMR tube under inert atmosphere and ^{31}P NMR was measured immediately (locked by residual D_2O signal).

Oxidative addition complex formation under ball milling condition

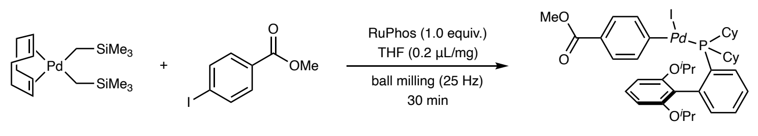

S19

To a ball milling vessel (1.5 ml, stainless) were added (COD)Pd(CH_2SiMe_3)$_2$ (47.0 mg, 0.12 mmol), methyl 4-iodobenzoate (31.5 mg, 0.12 mmol), RuPhos (60.0 mg, 0.12 mmol), THF (26 μL) and one grinding ball (stainless, diameter: 5 mm). The vessel was closed and then ball milled (25 Hz) for 30 min at room temperature. After ball milling, the vessel was opened under air and reaction mixture was passed through the short silica pad with AcOEt as eluent. Then the crude mixture was concentrated and washed with pentane three times under air. After drying under vacuum, desired complex **S19** was obtained (55.3 mg, 54%) as yellowish solid.

^{1}H NMR (500 MHz, $CDCl_3$) δ 7.64 (t, *J* = 8.0 Hz, 1H), 7.58 (t, *J* = 6.5 Hz, 1H), 7.54 (d, *J* = 7.5 Hz, 2H), 7.42 (t, *J* = 7.5 Hz, 1H), 7.36 (t, *J* = 7.5 Hz, 1H), 7.24 (dd, *J* = 8.3, 2.0 Hz, 2H), 6.84 (ddd, *J* = 7.8, 2.8, 1.0 Hz, 1H), 6.66 (d, *J* = 8.0 Hz, 2H), 4.59 (sep, 6.0 Hz, 2H), 3.84 (s, 3H), 2.16–2.05 (br, 2H), 1.83–1.71 (br, 6H), 1.69–1.58 (br, 6H), 1.39 (d, *J* = 6.0 Hz, 6H), 1.24–1.04 (m, 6H), 1.02 (d, *J* = 6.0 Hz, 6H), 0.85–0.69 (br, 2H).

^{13}C NMR (150 MHz, $CDCl_3$) δ 168.21, 159.49, 144.70, 144.58, 142.31, 138.84, 138.83, 135.13, 133.21, 132.98, 132.79, 132.72, 130.99, 130.79, 126.69, 126.55, 126.51, 125.08, 111.14, 107.96, 71.21, 51.83, 34.08, 33.90, 28.25, 27.69, 27.37, 27.28, 27.00, 26.93, 26.11, 22.32, 21.80.

^{31}P NMR (241 MHz, THF) δ 23.3. (locked by D_2O, calibrated by 80% H_3PO_4 in D_2O)

HRMS (ESI^+): calcd. for $C_{38}H_{50}IO_4PPdNa$ ($[M+Na]^+$): 857.1424, found: 857.1432 (error: 0.9 ppm).

6-2. Polymerization Reaction without Dendrimer Support

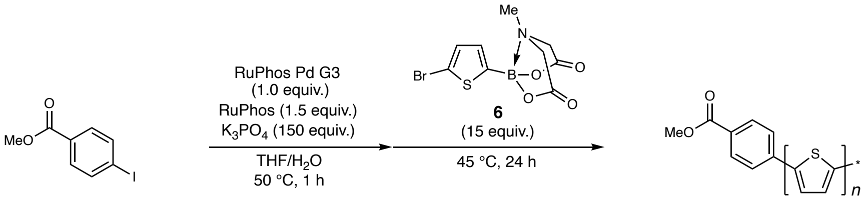

Oxidative complex formation and polymerization reaction were performed by same manner with on-dendrimer synthesis. Reaction mixture shows insoluble red precipitate (Supplementary Fig. 2, left). This insoluble component was collected by filtration and washed by chloroform, H_2O and THF and LDI-TOF mass spectroscopy was conducted (Supplementary Fig. 3). LDI-TOF mass spectrum shows signals corresponding to unsubstituted polythiophene with 8 to 10 thiophene rings, although 15 equivalent of monomer was used for polymerization reaction. This result indicates that unsubstituted oligothiophene becomes inactive to reaction when it contains at least 8 thiophene tings.

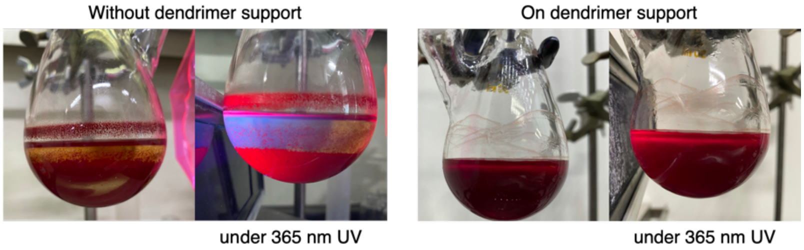

Supplementary Fig. 2. Reaction mixture of off-dendrimer polymerization (left) and on-dendrimer polymerization (right).

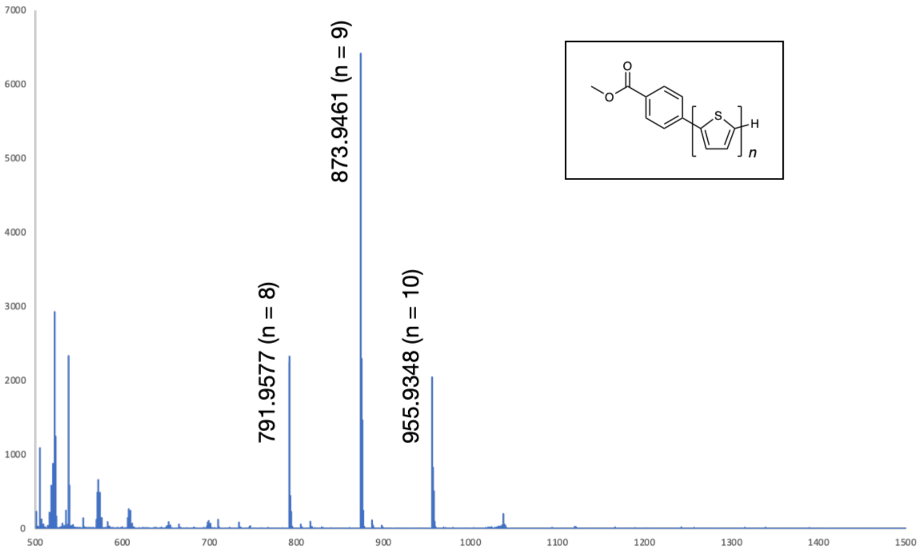

Supplementary Fig. 3. LDI-TOF mass spectrum of off-dendrimer polymerization product.

6-3. Polymerization Reaction with 50 and 100 Equiv. of Monomer

Because of the severe limitation in analytical methods (especially LDI-TOF MS), reliable identification of aromatic polymer with high molecular weight was difficult. In order to obtain some information about the limit of dendrimer-supported synthesis in terms of the polymer length, polymerization reaction was conducted with 50 and 100 equiv. of monomer. Since hydrolysis of thiophene monomer **6** is known to be very slow, we employed the thiophene monomer **S20** reported by Choi in this attempt[13]. New monomer **S20** was synthesized as shown in below.

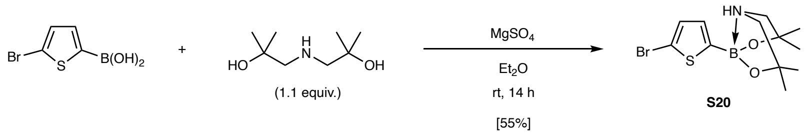

To a 100-mL round-bottom flask were added 5-bromo-2-thiopheneboronic acid (827.1 mg, 4.0 mmol), diol protecting group (709.3 mg, 4.4 mmol), $MgSO_4$ and Et_2O. The reaction mixture was stirred at ambient temperature for 14 hours. $MgSO_4$ was removed by filtration and filter cake was washed by acetone and AcOEt. Filtrate was concentrated to afford white crystalline solid. Pure product **S20** was obtained after reprecipitation from acetone/Et_2O as a white solid (734 mg, 55%). ^{1}H NMR (600 MHz, DMSO-d_6) δ 7.52 (bs, 1H), 7.00 (d, J = 3.4 Hz, 1H), 6.80 (d, J = 3.4 Hz, 1H), 2.99 (dd, J = 12.0, 7.6 Hz, 2H), 2.81 (dd, J = 12.0, 4.1 Hz, 2H), 1.30 (s, 6H), 1.19 (s, 6 H). ^{13}C NMR (150 MHz, DMSO-d_6) δ 130.31, 130.23, 110.79, 75.43, 60.55, 30.63, 29.39.

HRMS (ESI$^+$): calcd. for $C_{12}H_{19}SBrBO_2NNa$ ($[M+Na]^+$): 354.0305, found: 354.0303 (error: 0.6 ppm).

Polymerization reaction was conducted with 50 equiv. and 100 equiv. of monomer **S20**, respectively. The reaction mixture using 50 equiv. of monomer was clear red solution (Supplementary Fig. 4, left). Furthermore, in ^{1}H NMR spectrum of the reaction mixture, the signals of the monomer were not observed. On the other hand, red precipitate was clearly generated after the polymerization reaction where 100 equiv. of monomer was used (Supplementary Fig. 4, right). The ^{1}H NMR signals assigned to the monomer were also detected. Based on these results, we now concluded that polymerization successfully proceeded at least up to DP = ca. 50.

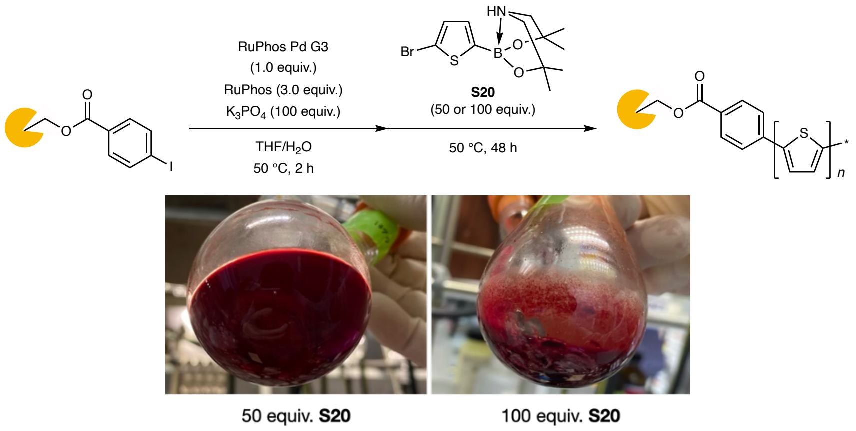

Supplementary Fig. 4. Reaction mixture of polymerization with 50 equiv. (right) and 100 equiv. (left) of monomer **S20**.

6-4. Model Reaction to Confirm Bond-formation between Dendrimer and Thiophene Ring

In order to confirm the connectivity between the dendrimer support and thiophene rings, model reaction and its ^{1}H NMR analysis were conducted. We synthesized the 2,2′-bithiophene boronic acid MIDA ester and Suzuki–Miyaura cross-coupling with dendrimer support was performed as shown below. Pd catalyst, ligand, base, solvent and temperature used to the coupling reaction were the same as those of polymerization reaction mentioned in the paper.

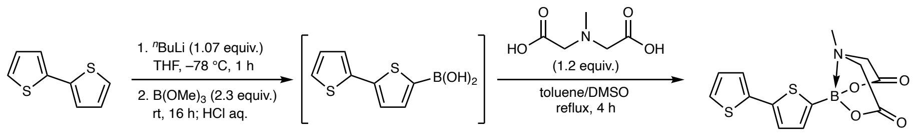

S21

To a flame dried 200-mL two-necked flask was added 2,2'-bithiophene (2.20 g, 13.3 mmol). The flask was filled with nitrogen followed by addition of THF (50 mL). The solution was cooled to −78 °C by dry ice/acetone bath and ^{n}BuLi (1.59 M in hexane, 8.90 mL, 14.2 mmol) was added dropwise to the cooled solution. The mixture was then stirred at −78 °C for 1 h. Then $B(OMe)_3$ (3.42 mL, 30.6 mmol) was added to the mixture in one portion. The reaction mixture was warmed up to room temperature and stirred for 16 h. 1M HCl aq. was added to the solution to quench the reaction and it was extracted with $CHCl_3$ three times. Combined organic layer was washed with brine, dried over Na_2SO_4 and concentrated under reduced pressure. Crude product was roughly purified by reprecipitation from Et_2O/hexane to afford the desired boronic acid (1.61 g). The roughly purified product was used to next reaction without further purification.

To a 100-mL round-bottom flask were added the boronic acid (1.05 g, 5.0 mmol), *N*-methyliminodiacetic acid (883.1 mg, 6.0 mmol), toluene (50 mL) and DMSO (5.0 mL). The solution was refluxed for 4 h with Dean-Stark apparatus to remove generating water. The reaction mixture was then concentrated and thus-obtained crude product was purified by silica gel column chromatography (eluent: hexane/AcOEt = 1:1 → 1:5) to afford **S21** (1.30 g, 47%, 2 steps) as a white solid. ^{1}H NMR spectrum of **S21** agrees with reported data[14].

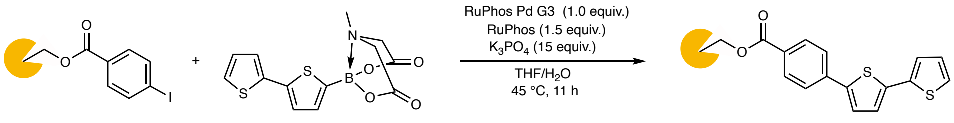

5 **S21** (1.5 equiv.)

To a screw capped tube were added dendrimer support **5** (66.8 mg, 8.0 µmol), **S21** (3.9 mg, 12 µmol), RuPhos Pd G3 (6.7 mg, 8.0 µmol), RuPhos (5.6 mg, 12 µmol) and K_3PO_4 (25.6 mg, 120 µmol). The tube was filled with nitrogen followed by addition of THF (2.0 mL) and H_2O (0.72 mL). The reaction mixture was stirred at 45 °C for 18 h. The reaction mixture was diluted by $CHCl_3$ and then passed through a pad of Celite®. Concentrated filtrate was purified by GPC to afford the desired product (56.1 mg, 84%) as a colorless sticky solid. ^{1}H NMR analysis of the product is shown in Supplementary Fig. 5 (green line).

^{1}H NMR spectrum of coupling product was measured and compared to those of starting materials. Signals of dendrimer support (Supplementary Fig. 5, blue line) around 7.8 ppm are assigned to aryl iodide moiety and peripheral triazoles. Signals of aryl iodide moiety also appeared around 7.8 ppm in case of alkyne-terminated dendrimer **3** (Supplementary Fig. 22). On the other hand, coupling product (Supplementary Fig. 5, green line) shows two doublet signals of aryl iodide moiety at 8.07 ppm and 7.63 ppm[15]. In addition, signals of 2,2′-bithiophene structure were observed at 7.01 ppm (dd) and 7.2 ppm (multiplet). Based on these results, we believe that Suzuki–Miyaura coupling and well-known catalyst-transfer should successfully proceed on the dendrimer support to afford dendrimer-ligated polythiophene.

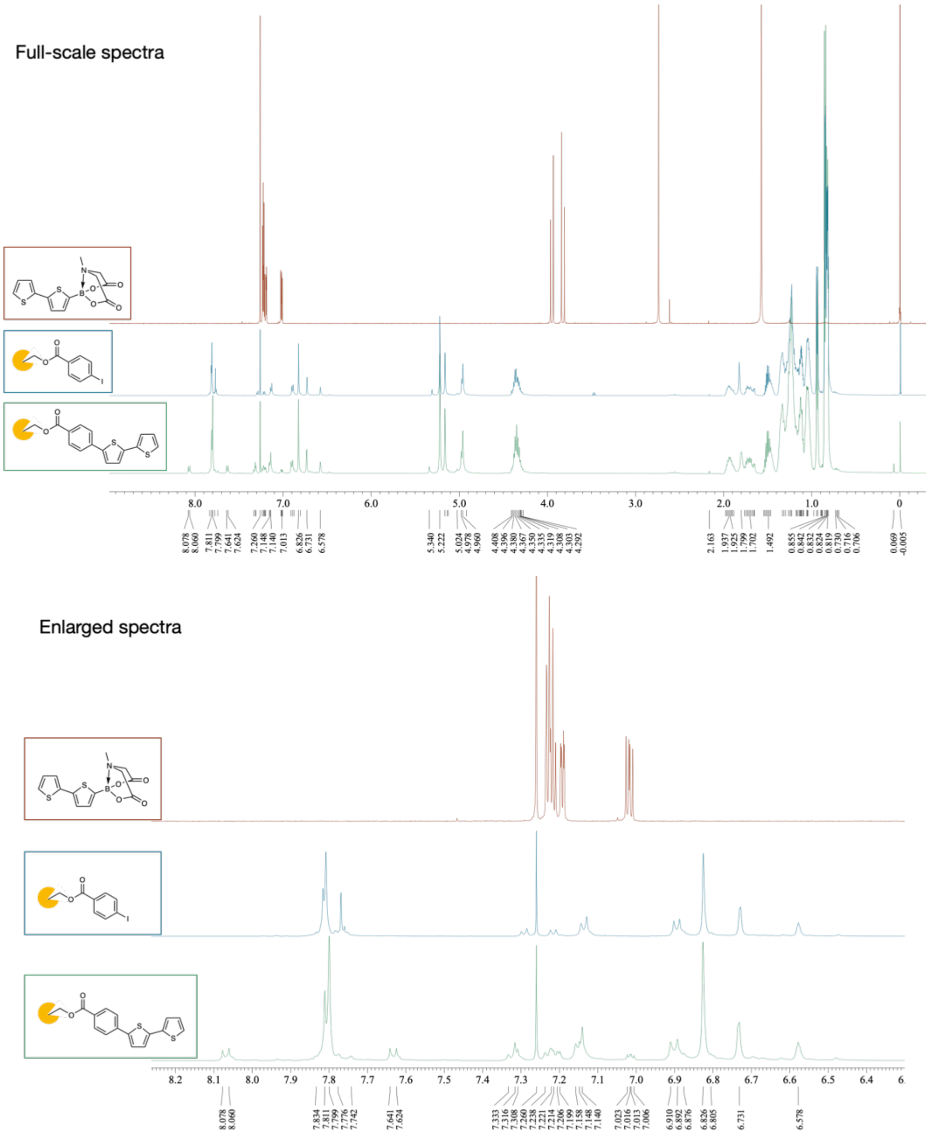

Supplementary Fig. 5. ^{1}H NMR spectra of starting materials and product of model reaction.

7. Spectroscopic Measurements

7-1. UV/vis Absorption and Fluorescence Spectra of 7 in Chloroform

UV/vis absorption spectra were recorded on a Shimadzu UV-3600 spectrometer with a resolution of 0.5 nm. Fluorescence spectra were measured on Shimadzu RF-6000 spectrometer with a resolution of 0.2 nm. Dilute solutions in spectral grade solvents in a 1 cm square quartz cell were used for measurements.

In chloroform solution, an emission band in 568 nm was observed, which was in shorter wavelength than the shoulder absorption band in 575 nm.

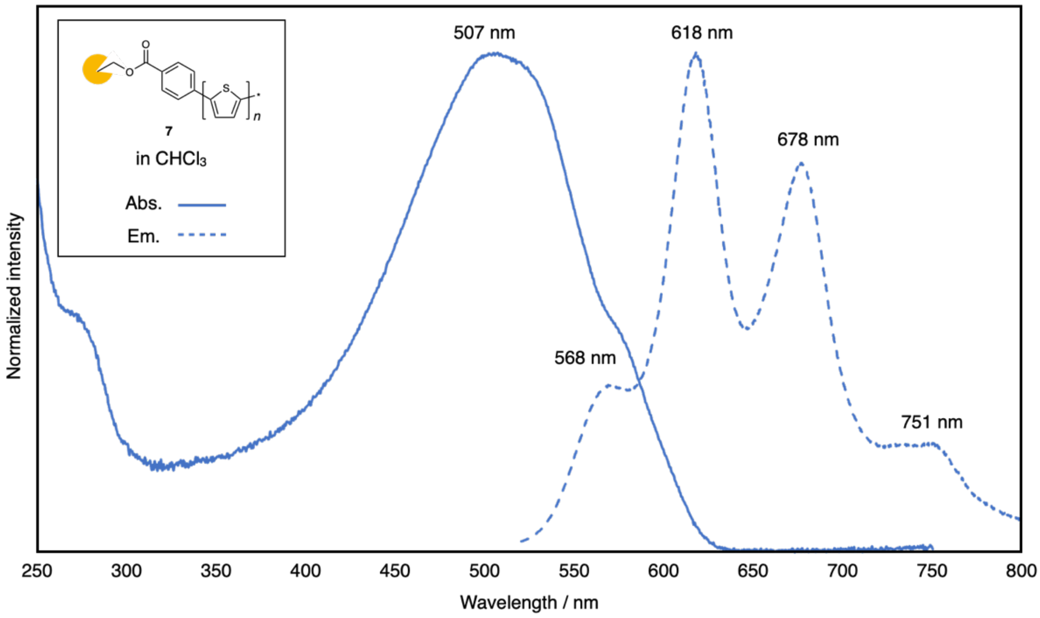

Supplementary Fig. 6. UV/vis absorption (solid line) and fluorescence (broken line) spectra of **7** in chloroform. Fluorescence spectrum was taken upon excitation at 507 nm.

7-2. Solvent Effect for Absorption and Fluorescence Spectra

To gain insight into the photophysical properties of **7** in chloroform depicted in Section 7-1, the solvent effect for absorption and fluorescence spectra was investigated. The emission band in 568 nm were observed in chloroform albeit not observed in THF and hexane. This behavior of **7** is now under investigation in our laboratory.

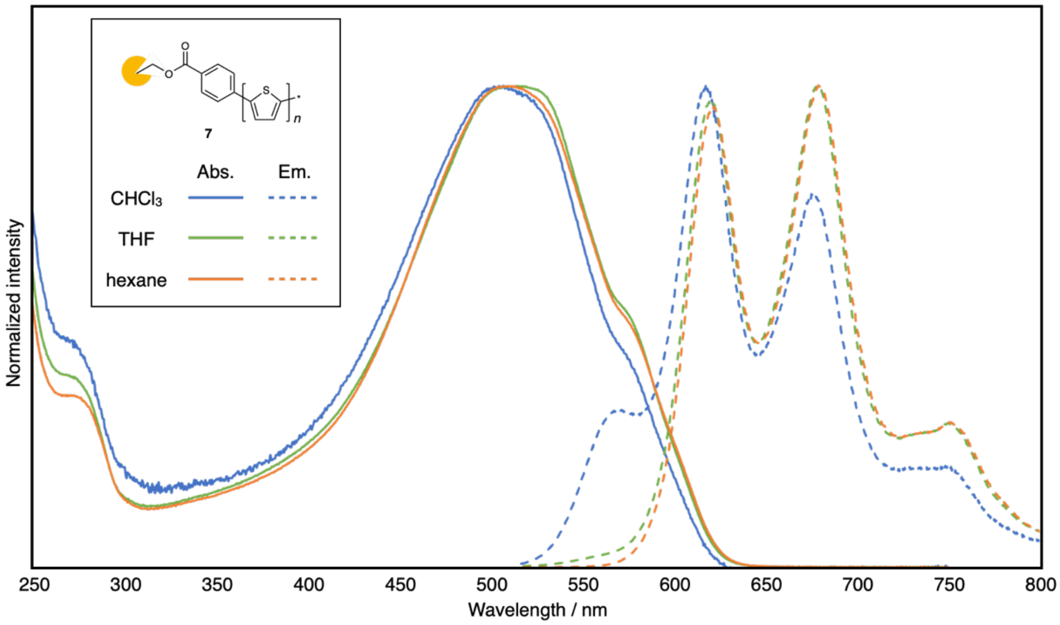

Supplementary Fig. 7. UV/vis (solid line) and fluorescence (broken line) spectra of **7** in chloroform (blue line), THF (green line) and hexane (orange line). Fluorescence spectra were taken upon excitation at 507 nm (chloroform) and 510 nm (THF and hexane).

7-3. Dynamic Light-Scattering (DLS) Measurement of 7

Hydrodynamic radius of **7** was determined by DLS measurement on a Wyatt Technology DynaPro NanoStar. Observed hydrodynamic radius was 3.6 nm (%Number = 100) and other particle size was negligible.

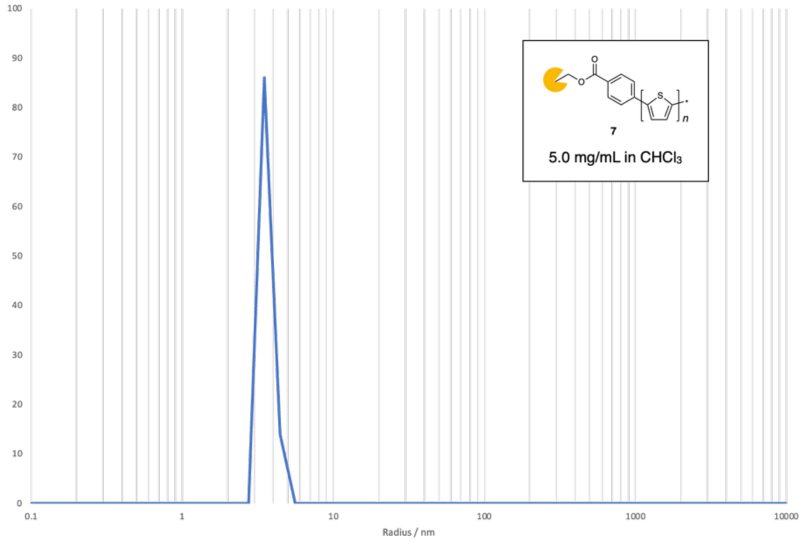

Supplementary Fig. 8. Hydrodynamic radius of **7** determined by dynamic light-scattering.

7-4. LDI-TOF Mass Spectrum of Unsubstituted Aromatic Polymer

Since unsubstituted aromatic polymers are highly insoluble to organic solvents, LDI-TOF mass measurement was applied to its mass spectrometry. End-group analysis shows that chain-ends were successfully capped by hydrogen atom by HCl aq. quenching in all aromatic polymers. Opposite chain-end was mainly benzylic alcohol structure whose signals are marked by ●. In addition, aromatic polymers with *p*-tolyl end-group (marked by ■) which is presumably over-reduction product of cleavage reaction was also observed. In the case of poly(*para*-phenylene) and polybenzotriazole, off-dendrimer polymerization products (*i.e.* self-polymerization product of monomers) were also detected in relatively low molecular weight region (marked by ▼).

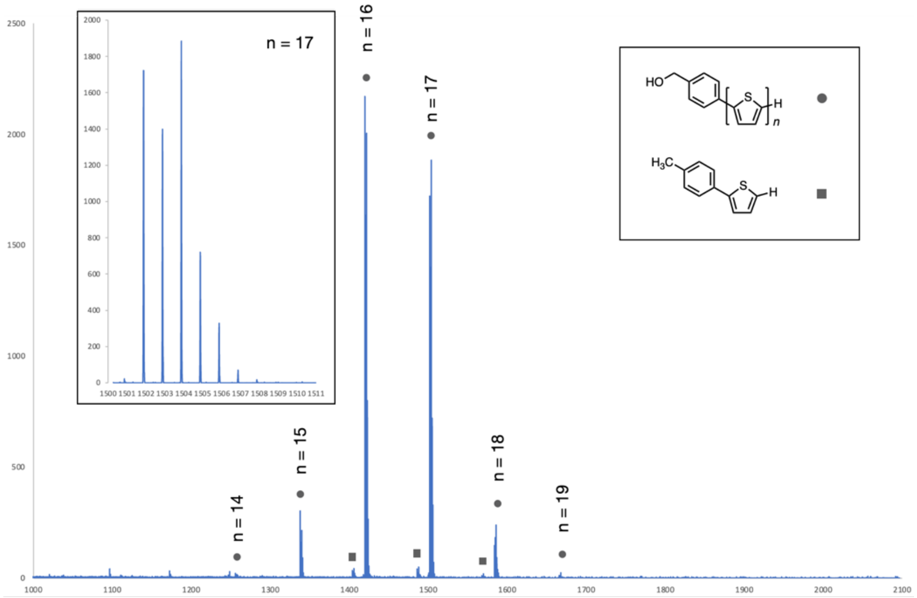

n	15	16	17
observed *m/z*	1337.8861	1419.8783	1501.8673
calculated *m/z*	1337.8733	1419.8610	1501.8488
Δm/z	81.9922	81.9890	

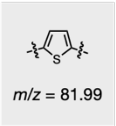

Supplementary Fig. 9. LDI-TOF mass spectrum of unsubstituted polythiophene **8** and its observed and calculated molecular ion peaks (monoisotopic mass value).

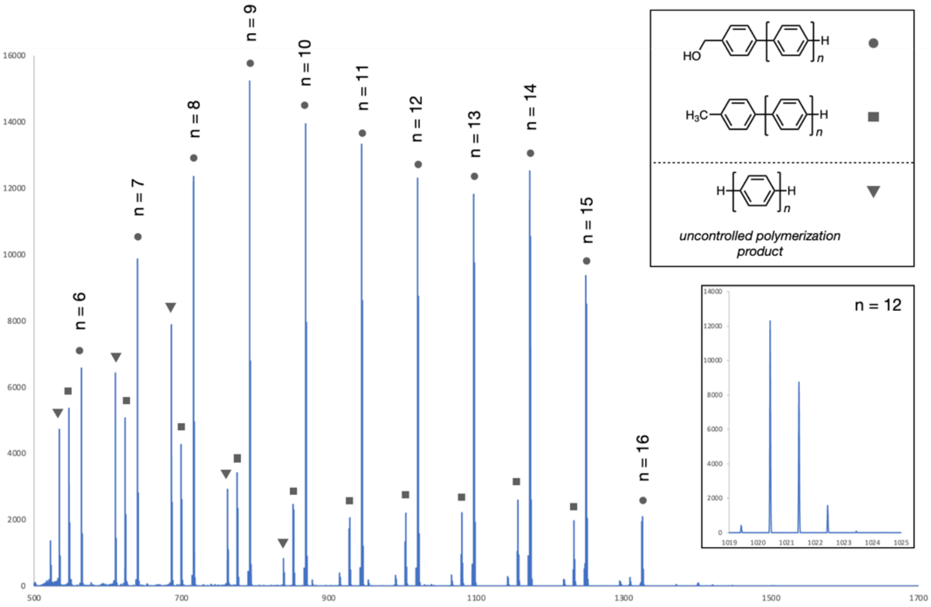

n	7	8	9	10	11	12	13	14	15	16
observed *m/z* ●	640.2765	716.3058	792.3363	868.3668	944.3986	1020.4302	1096.4639	1172.4972	1248.5324	1324.5695
calculated *m/z*	640.2766	716.3079	792.3392	868.3705	944.4018	1020.4331	1096.4644	1172.4957	1248.5270	1324.5583
Δm/z		76.0293	76.0305	76.0305	76.0318	76.0316	76.0337	76.0333	76.0352	76.0371

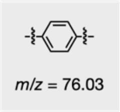

Supplementary Fig. 10. LDI-TOF mass spectrum of unsubstituted poly(*para*-phenylene) **11** and its observed and calculated molecular ion peaks (monoisotopic mass value).

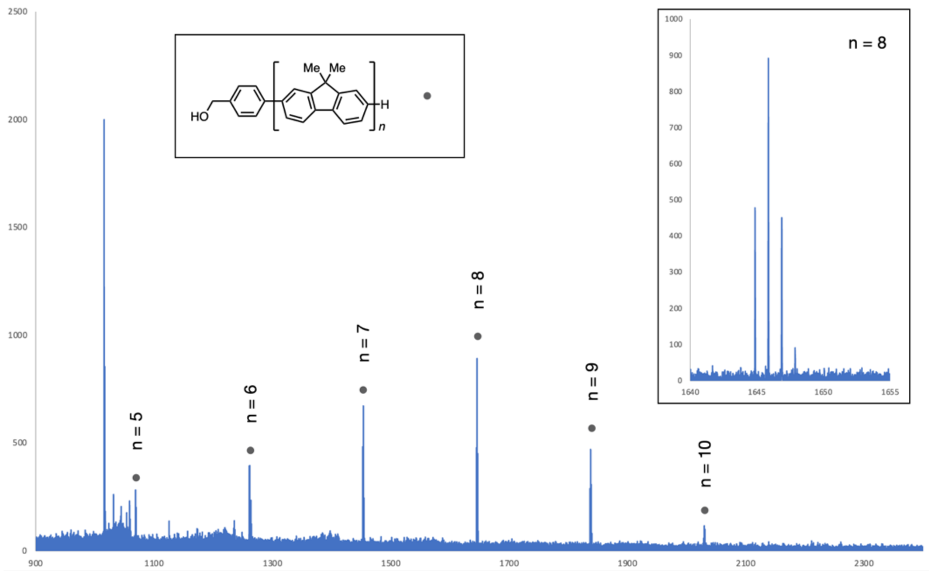

n	5	6	7	8	9
observed *m/z*	1068.5552	1260.6539	1452.7544	1644.8570	1836.9586
calculated *m/z*	1068.5270	1260.6209	1452.7148	1644.8087	1836.9026
Δm/z		192.10	192.10	192.10	192.10

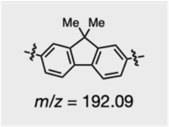

Supplementary Fig. 11. LDI-TOF mass spectrum of unsubstituted polyfluorene **14** and its observed and calculated molecular ion peaks (monoisotopic mass value).

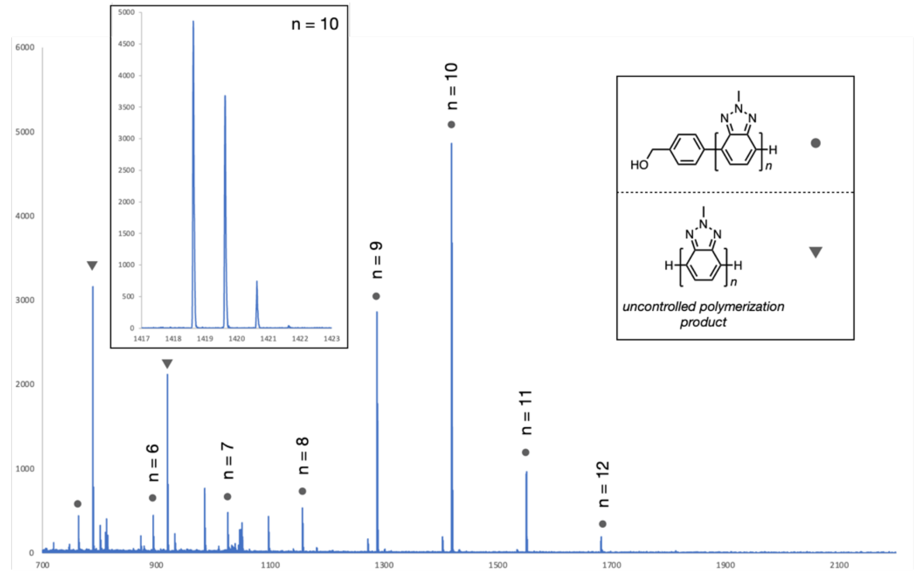

n	6	7	8	9	10	11	12
observed *m/z*	894.4060	1025.4629	1156.5209	1287.5788	1418.6364	1549.6940	1680.7486
calculated *m/z*	894.3476	1025.3959	1156.4443	1287.4926	1418.5410	1549.5893	1680.6377
Δm/z		131.0569	131.0580	131.0579	131.0576	131.0576	131.0546

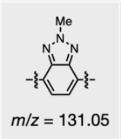

Supplementary Fig. 12. LDI-TOF mass spectrum of unsubstituted polybenzotriazole **17** and its observed and calculated molecular ion peaks (monoisotopic mass value).

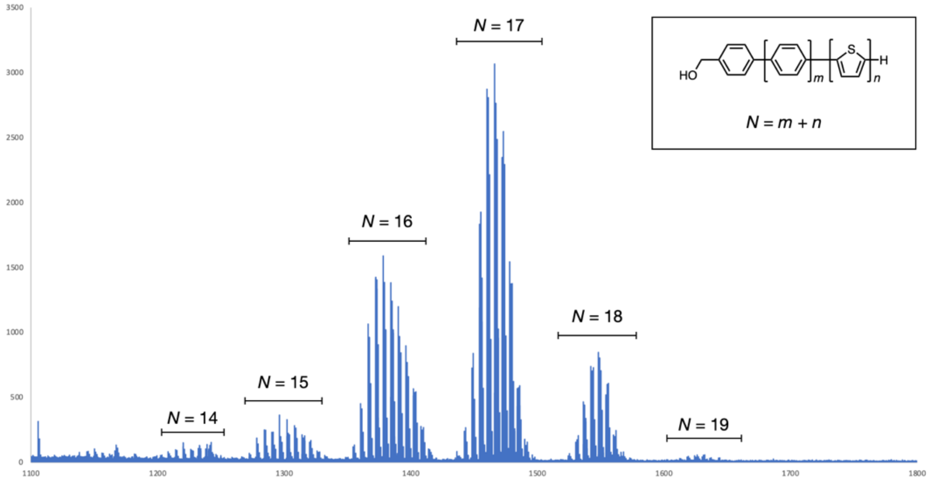

Supplementary Fig. 13. LDI-TOF mass spectrum of unsubstituted poly(*para*-phenylene)-*block*-polythiophene **19**.

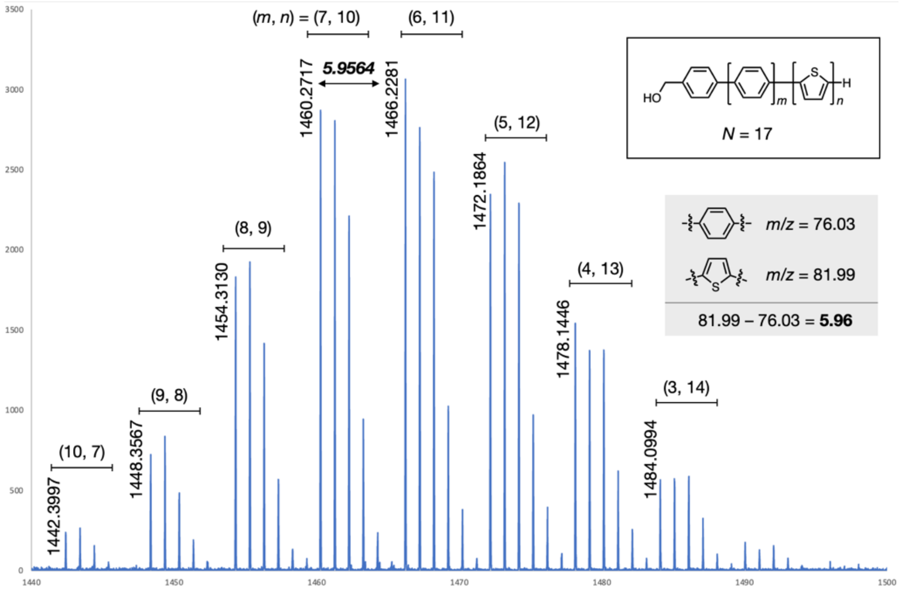

Supplementary Fig. 14. Enlarged view of LDI-TOF mass spectrum of **19**.

7-5. IR Spectroscopy

Fourier-transform infrared (FT-IR) spectra were recorded using a JASCO FT/IR-6600 spectrometer. IR spectra of unsubstituted polythiophene **8** and poly(*para*-phenylene) **11** were measured by KBr pellet method.

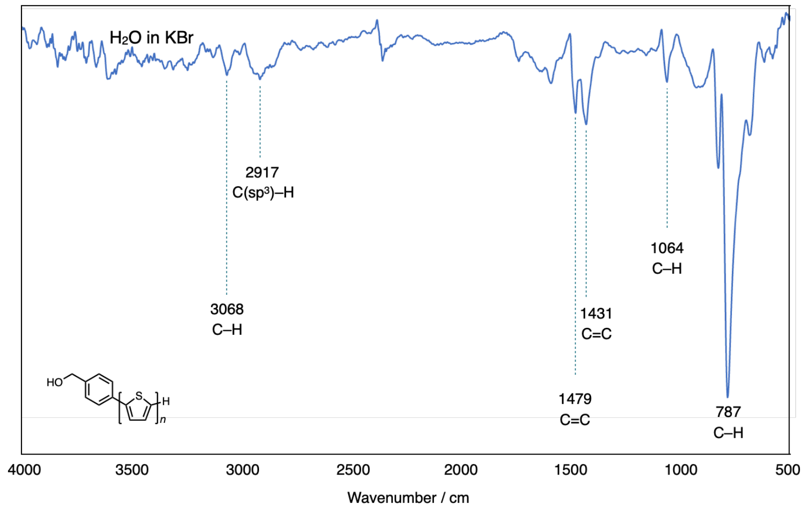

Supplementary Fig. 15. IR spectrum of unsubstituted polythiophene **8**.

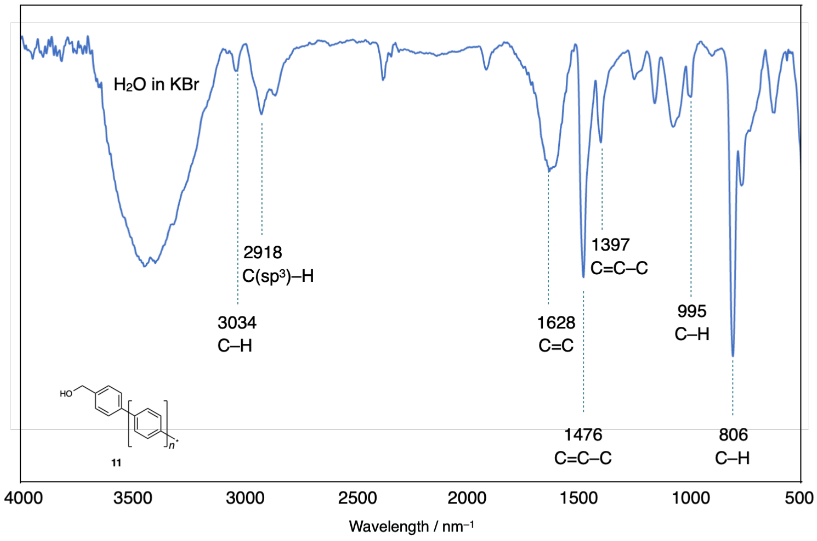

	Müllen's PPP	our PPP
C–H out-of-plane deformation	810 cm^{-1}	806 cm^{-1}
C–H in-plane deformation	1001 cm^{-1}	995 cm^{-1}
C=C–C bending	1399 cm^{-1}	1397 cm^{-1}
C=C–C bending	1480 cm^{-1}	1476 cm^{-1}
C=C stretching	1601 cm^{-1}	1628 cm^{-1}
C–H stretching	3030 cm^{-1}	3034 cm^{-1}

Supplementary Fig. 16. IR spectrum of unsubstituted poly(*para*-phenylene) and comparison to with reported IR data[16].

8. Material Hybridization

8-1. Hybridization with Silica Gel

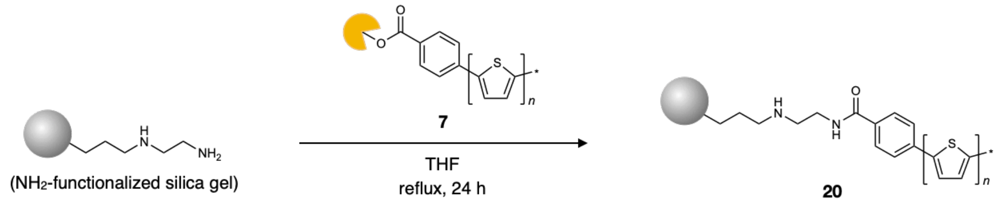

To a flame-dried 25-mL Schlenk tube were added dendrimer-ligated polythiophene **7** (20 mg), NH_2-functionalized silica gel (1.08 g) and dry THF (5 mL). The reaction mixture was gently stirred at room temperature for 24 h. The solvent was removed by decantation. The thus-obtained silica gel was carefully washed by $CHCl_3$ (5 times) and THF (2 times) by centrifugation and decantation to afford red silica gel.

Resulting silica gel shows orange fluorescence indicating successful transfer of polythiophene backbone from dendrimer to silica gel surface (Fig. 6). IR spectrum of dendrimer support (gray line) shows strong C(sp^3)–H stretching band around 2900 cm^{-1} derived from long alkyl chain in its periphery (Supplementary Fig. 17). On the other hand, IR spectrum of **20** shows no distinct absorption band in this C(sp^3)–H stretching region compared to it of pristine silica gel (blue line). This result support that nonspecific adsorption of dendrimer-ligated polythiophene to silica gel is negligible.

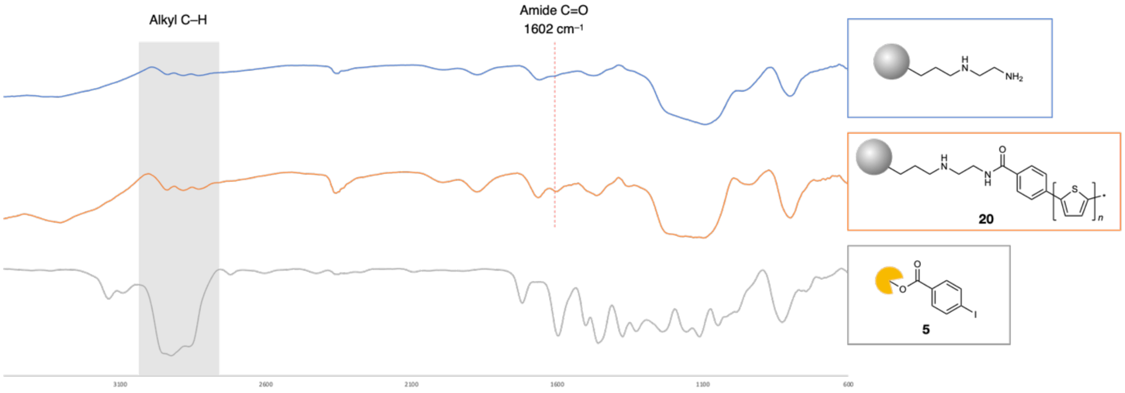

Supplementary Fig. 17. IR spectra of amine-functionalized silica gel (blue line), silica gel-polythiophene conjugate **20** (orange line) and dendrimer support (gray line). IR spectra was measured by diffuse reflection method with KBr dilution.

8-2. Hybridization with Protein

Preparation of polythiophene-conjugated HSA 21

A THF solution of dendrimer-ligated polythiophene **7** (5.0 mg/mL, 0.5 mL) was added to a PBS(–) containing human serum albumin (HSA; purchased from Sigma-Aldrich (A3782), 400 μM, 0.5 mL), followed by incubation at 37 °C for 13 h. Insoluble **7** was removed by centrifugation for 3 min at 500 g to collect the soluble fraction. The fraction was dialyzed three times with PBS(–) with a Slide-A-Lyzer™ Dialysis Cassette (3,500 MWCO) (Thermo Fisher Scientific) to remove THF. To validate covalently binding between bare polythiophene and HSA, SDS-PAGE (BioRad Mini-Protean Tetra cell electrophoresis apparatus) was carried out. The solution of polythiophene-conjugated HSA was mixed with 2x laemmli buffer (BioRad) containing 2-mercaptoethanol and boiled at 95 °C for 5 min. The samples were applied to a 12% SDS–PAGE gel (BioRad) and imaged by Coomassie Brilliant Blue (CBB) stain and in-gel fluorescence with an Amersham ImageQuant 800 system (Cytiva) (Supplementary Fig. 18)

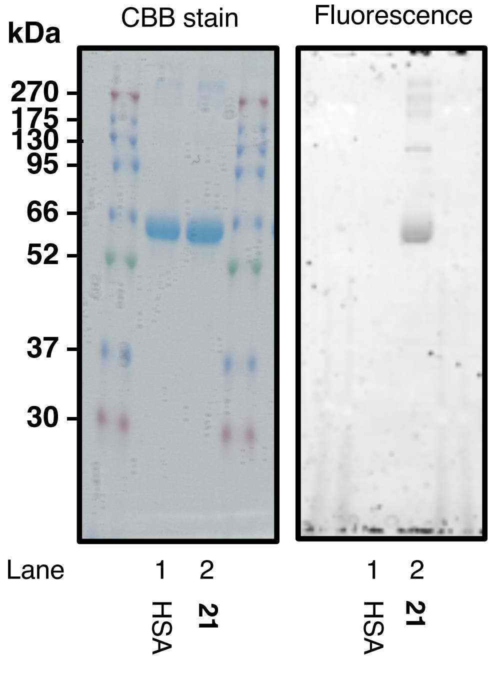

Supplementary Fig. 18. SDS–PAGE of HSA and **21** imaged by Coomassie Brilliant Blue (CBB) stain and in-gel fluorescence.

Visualization of polythiophene-conjugated HSA in HEK293T cells

HEK293T cells (ATCC, 1x10^5 cells) were seeded on poly-L-lysine coated glass area of 35 mm glass bottomed dishes (Matsunami) and incubated in high glucose Dulbecco's Modified Eagle Medium (DMEM, Sigma-Aldrich) supplemented with 10% fetal bovine serum (FBS, Sigma-Aldrich) and 1% Antibiotic-Antimycotic (Anti-Anti, Sigma-Aldrich) under a humidified atmosphere of 5% CO_2 in air at 37°C for 14 h. The cell was incubated in a PBS solution of polythiophene-conjugated HSA under a humidified atmosphere of 5% CO_2 in air at 37°C for 6 h. After incubation, cells were washed with PBS(–) three times, followed by Cell imaging with a confocal laser scanning microscope equipped with a 63× objectives lens (Carl Zeiss LSM900) (Supplementary Fig. 19).

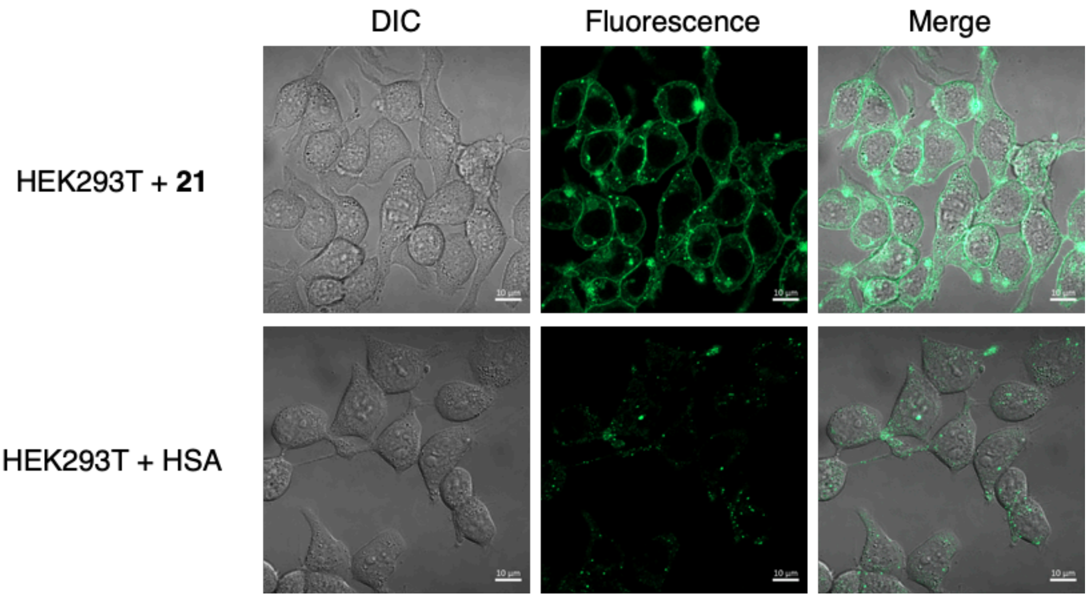

Supplementary Fig. 19. Confocal scope images of polythiophene-conjugated HSA in HEK293T cells.

9. Computational Data

In order to estimate theoretical length of unsubstituted polythiophene with 15 thiophene rings, the structure of polythiophene (n = 15) was optimized. The Gaussian 16 program[17] running on a NEC LX 110Rh system was used for optimization (B3LYP/6-31G(d))[18,19]. Structures were optimized without any symmetry assumptions.

Supplementary Table. 1. Cartesian coordinates of optimized unsubstituted polythiophene (n = 15)

C	0.4563	-1.1508	-0.0003
C	1.8827	-1.0919	-0.0003
C	-0.0313	-2.4446	-0.0003
S	1.2703	-3.5647	-0.0002
C	2.4752	-2.3412	-0.0003
C	3.89	-2.6632	-0.0003
S	5.0958	-1.4405	-0.0003
C	4.4817	-3.9129	-0.0002
C	5.908	-3.855	-0.0002
C	6.3966	-2.5616	-0.0003
H	3.9288	-4.8445	-0.0002
H	6.5347	-4.7388	-0.0002
H	2.4351	-0.16	-0.0003
H	-0.1711	-0.2676	-0.0003
S	-6.5394	-3.8931	-0.0002
S	-2.7172	-1.763	-0.0003
C	-3.3275	-4.2364	-0.0002
C	-7.8428	-2.7751	-0.0003
C	-7.3572	-1.4805	-0.0003
C	-5.9308	-1.4194	-0.0003
C	-5.3364	-2.6677	-0.0003
C	-1.9014	-4.1764	-0.0002
C	-3.9211	-2.9876	-0.0003
C	-1.4147	-2.8822	-0.0003
H	-3.8791	-5.1688	-0.0002
H	-7.9859	-0.5982	-0.0003
H	-1.2734	-5.0592	-0.0002
H	-5.3798	-0.4866	-0.0003
S	9.0808	-3.2478	-0.0002
S	12.9087	-1.1282	-0.0003
C	12.292	-3.6	-0.0002
C	7.7806	-2.126	-0.0003
C	8.2699	-0.8328	-0.0003
C	9.6964	-0.7758	-0.0003
C	10.2873	-2.0259	-0.0003
C	13.7184	-3.5437	-0.0002
C	11.7017	-2.3497	-0.0003
C	14.2083	-2.2507	-0.0003
H	11.738	-4.5309	-0.0002
H	7.6437	0.0513	-0.0004
H	14.3441	-4.428	-0.0002
H	10.2501	0.1554	-0.0004
S	-29.9709	-4.9397	-0.0002
S	-22.1585	-4.5812	-0.0002
S	-14.3489	-4.2329	-0.0002
S	-26.152	-2.7929	-0.0003
S	-18.3412	-2.4422	-0.0003
S	-10.5296	-2.0975	-0.0003
C	-11.1363	-4.5718	-0.0002
C	-26.7549	-5.268	-0.0002

C	-17.5192	-4.8534	-0.0002
C	-18.9453	-4.917	-0.0002
C	-25.3288	-5.2038	-0.0002
C	-31.251	-3.8111	-0.0003
C	-28.7686	-3.7052	-0.0003
C	-30.7932	-2.5153	-0.0003
C	-27.3522	-4.0211	-0.0003
C	-24.8461	-3.9082	-0.0003
C	-19.542	-3.6698	-0.0003
C	-9.7102	-4.5097	-0.0002
C	-29.368	-2.4551	-0.0003
C	-17.0359	-3.558	-0.0003
C	-23.4642	-3.4659	-0.0003
C	-20.9581	-3.3532	-0.0003
C	-22.9813	-2.1703	-0.0003
C	-21.5552	-2.1062	-0.0003
C	-15.1698	-1.8214	-0.0003
C	-15.6537	-3.1166	-0.0003
C	-13.7436	-1.7584	-0.0003
C	-13.1475	-3.006	-0.0003
C	-11.7317	-3.3239	-0.0003
C	-9.2255	-3.2148	-0.0003
H	-11.6866	-5.505	-0.0002
H	-27.3043	-6.2018	-0.0002
H	-19.4946	-5.8508	-0.0002
H	-16.8891	-5.7346	-0.0002
H	-24.6984	-6.0848	-0.0002
H	-9.081	-5.3916	-0.0002
H	-32.2774	-4.1516	-0.0002
H	-31.4452	-1.6504	-0.0003
H	-15.7997	-0.94	-0.0004
H	-23.612	-1.2895	-0.0003
H	-21.0063	-1.1722	-0.0003
H	-13.1938	-0.8249	-0.0003
H	-28.8162	-1.5228	-0.0003
S	16.892	-2.9394	-0.0003
S	24.707	-2.641	-0.0003
S	20.7214	-0.8222	-0.0003
C	20.1034	-3.2937	-0.0002
C	21.5299	-3.238	-0.0002
C	19.5137	-2.0431	-0.0003
C	15.5928	-1.8164	-0.0003
C	18.0995	-1.7184	-0.0003
C	22.0204	-1.9454	-0.0003
C	23.4053	-1.5117	-0.0003
C	17.5097	-0.4679	-0.0003
C	16.0832	-0.5237	-0.0003
C	23.8977	-0.2158	-0.0003
C	25.8878	-1.409	-0.0003
C	25.323	-0.1561	-0.0003
H	19.5492	-4.2245	-0.0002
H	22.1557	-4.1224	-0.0002
H	23.2696	0.6669	-0.0003
H	15.4578	0.3609	-0.0004
H	18.0642	0.4628	-0.0004
H	25.9001	0.7604	-0.0003
H	26.9392	-1.6622	-0.0003

10. Supplementary References

[1] Ignatenko, V. A., Deligonul, N. & Viswanathan, R. Branch-selective synthesis of oxindole and indene scaffolds: transition metal-controlled intramolecular aryl amidation leading to C3 reverse-prenylated oxindoles. *Org. Lett.* **12**, 3594–3597 (2010).

[2] Takahashi, D., Inomata, T. & Fukui, T. AJIPHASE®: a highly efficient synthetic method for one-pot peptide elongation in the solution phase by an Fmoc strategy. *Angew. Chem. Int. Ed.* **56**, 7803–7807 (2017).

[3] Fong, C., Wells, D., Krodkiewska, I., Hartley, P. G. & Drummond, C. New role for urea as a surfactant headgroup promoting self-assembly in water. *Chem. Mater.* **18**, 594–597 (2006).

[4] Qin, T., Li, X., Chen, J., Zeng, Y., Yu, T., Yang, G. & Li, Y. Dendritic ionic liquids based on imidazolium-modified poly(aryl ether) dendrimers. *Chem. Asian J.* **9**, 3641–3649 (2014).

[5] Camponovo, J., Ruiz, J., Cloutet, E. & Astruc, D. New polyalkynyl dendrons and dendrimers: "click" chemistry with azidomethylferrocene and specific anion and cation electrochemical sensing properties of the 1,2,3-triazole-containing dendrimers. *Chem. Eur. J.* **15**, 2990–3002 (2009).

[6] Hatano, T. & Kato, T. Nanostructured columnar and cubic liquid-crystalline assemblies consisting of unconventional rigid mesogens based on triphenylmethanes. *Tetrahedron* **64**, 8368–8380 (2008).

[7] Gillis, E. P. & Burke, M. D. A simple and modular strategy for small molecule synthesis: iterative Suzuki–Miyaura coupling of B-protected haloboronic acid building blocks. *J. Am. Chem. Soc.* **129**, 6716–6717 (2007).

[8] Singh, V., Wang, S. & Kool, E. T. Genetically encoded multispectral labeling of proteins with polyfluorophores on a DNA backbone. *J. Am. Chem. Soc.* **135**, 6184–6191 (2013).

[9] Yang, R., Tian, R., Yan, J., Zhang, Y., Yang, J., Hou, Q., Yang, W., Zhang, C. & Cao, Y. Deep-red electroluminescent polymers: synthesis and characterization of new low-band-gap conjugated copolymers for light-emitting diodes and photovoltaic devices. *Macromolecules* **38**, 244–253 (2005).

[10] Bridges, C. R., McCormick, T. M., Gibson, G. L., Hollinger, J. & Seferos, D. S. Designing and refining Ni(II)diimine catalysts toward the controlled synthesis of electron-deficient conjugated polymers. *J. Am. Chem. Soc.* **135**, 13212–13219 (2013).

[11] Bruneau, A., Roche, M., Alami, M. & Messaoudi, S. 2-Aminobiphenyl palladacycles: the "most powerful" precatalysts in C−C and C−heteroatom cross-couplings. *ACS Catal.* **5**, 1386–1396 (2015).

[12] Kubota, K., Takahashi, R. & Ito, H. Mechanochemistry allows carrying out sensitive organometallic reactions in air: glove-box-and-Schlenk-line-free synthesis of oxidative addition complexes from aryl halides and palladium(0). *Chem. Sci.* **10**, 5837–5842 (2019).

[13] Lee. J., Kim, H., Park, H., Kim, T., Hwang, S.-H., Seo, D., Chung, T. D. & Choi, T. L. Universal Suzuki–Miyaura catalyst-transfer polymerization for precision synthesis of strong donor/acceptor-based conjugated polymers and their sequence engineering. *J. Am. Chem. Soc.* **143**, 11180–11190 (2021).

[14] Xu, L., Ding, S. & Li, P. Site-differentiated polyboron arenes prepared by direct C–H borylation and their highly selective Suzuki–Miyaura cross-coupling reactions. *Angew. Chem., Int. Ed.* **53**, 1822–1826 (2014).

[15] Leowanawat, P., Zhang, N., Resmerita, A.-M., Rosen, B. M. & Percec, V. $Ni(COD)_2/PCy_3$ catalyzed cross-coupling of aryl and heteroaryl neopentylglycolboronates with aryl and heteroaryl mesylates and sulfamates in THF at room temperature. *J. Org. Chem.* **76**, 9946–9955 (2011).

[16] Abdulkarim, A., Hinkel, F., Jänsch, D., Freudenberg, J., Golling, F. E. & Müllen, K. A new solution to an old problem: synthesis of unsubstituted poly(*para*-phenylene). *J. Am. Chem. Soc.* **138**, 16208–16211 (2016).

[17] Frisch, M. J. *et al.* Gaussian 16, Revision B.01, Gaussian, Inc., Wallingford CT, 2016.

[18] Becke, A. D. Density functional thermochemistry. III. The role of exact exchange. *J. Chem. Phys.* **98**, 5648–5652 (1993).

[19] Lee, C., Yang, W. & Parr, R. G. Development of the Colle-Salvetti correlation-energy formula into a functional of the electron density. *Phys. Rev. B* **37**, 785–789 (1988).

10. ^{1}H and ^{13}C NMR Spectra

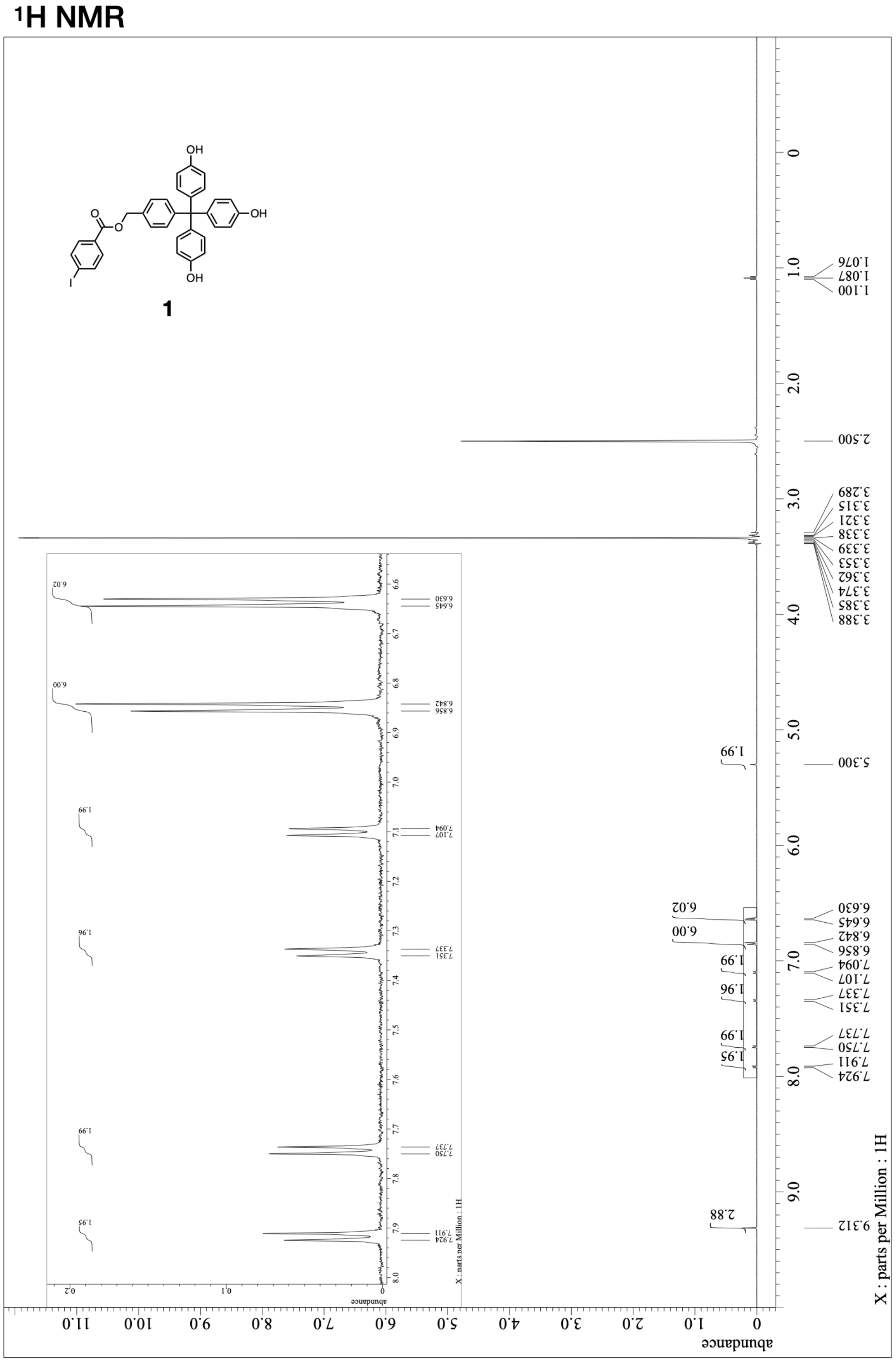

Supplementary Fig. 20. ^{1}H NMR spectrum of **1** (DMSO-d_6, 600 MHz).

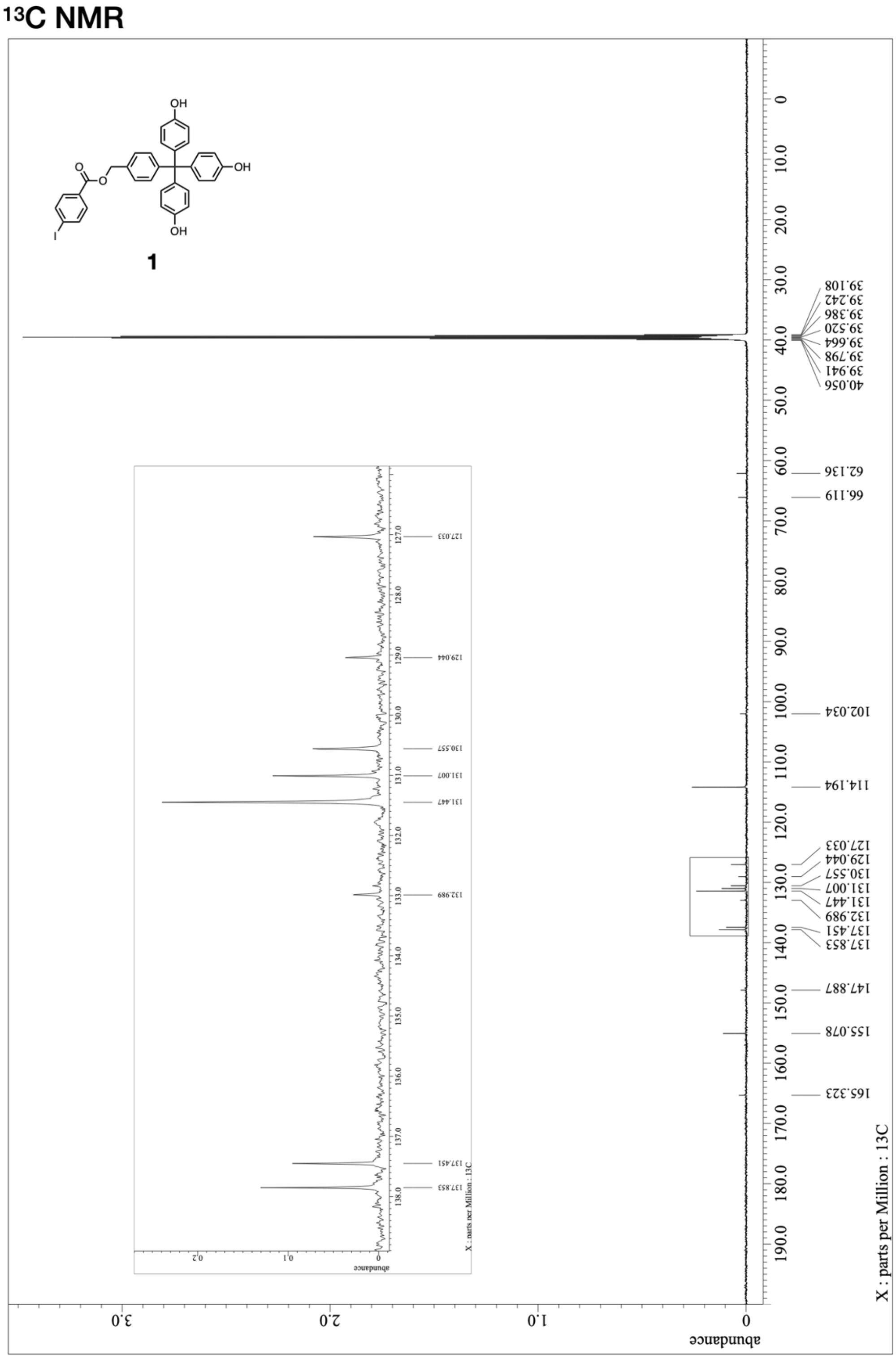

Supplementary Fig. 21. ^{13}C NMR spectrum of **1** (DMSO-d_6, 150 MHz).

^{1}H NMR

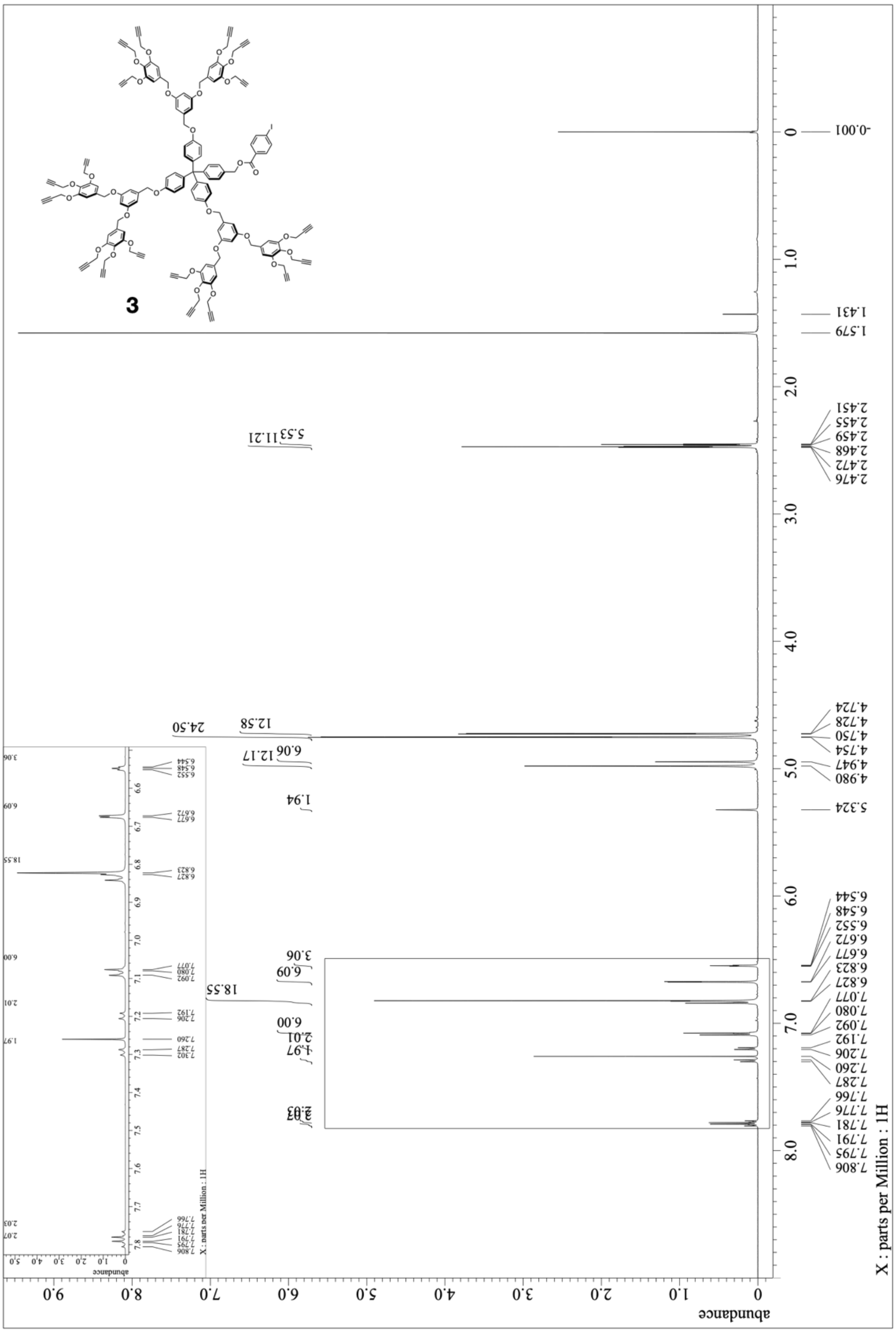

Supplementary Fig. 22. ^{1}H NMR spectrum of **3** ($CDCl_3$, 600 MHz).

^{13}C NMR

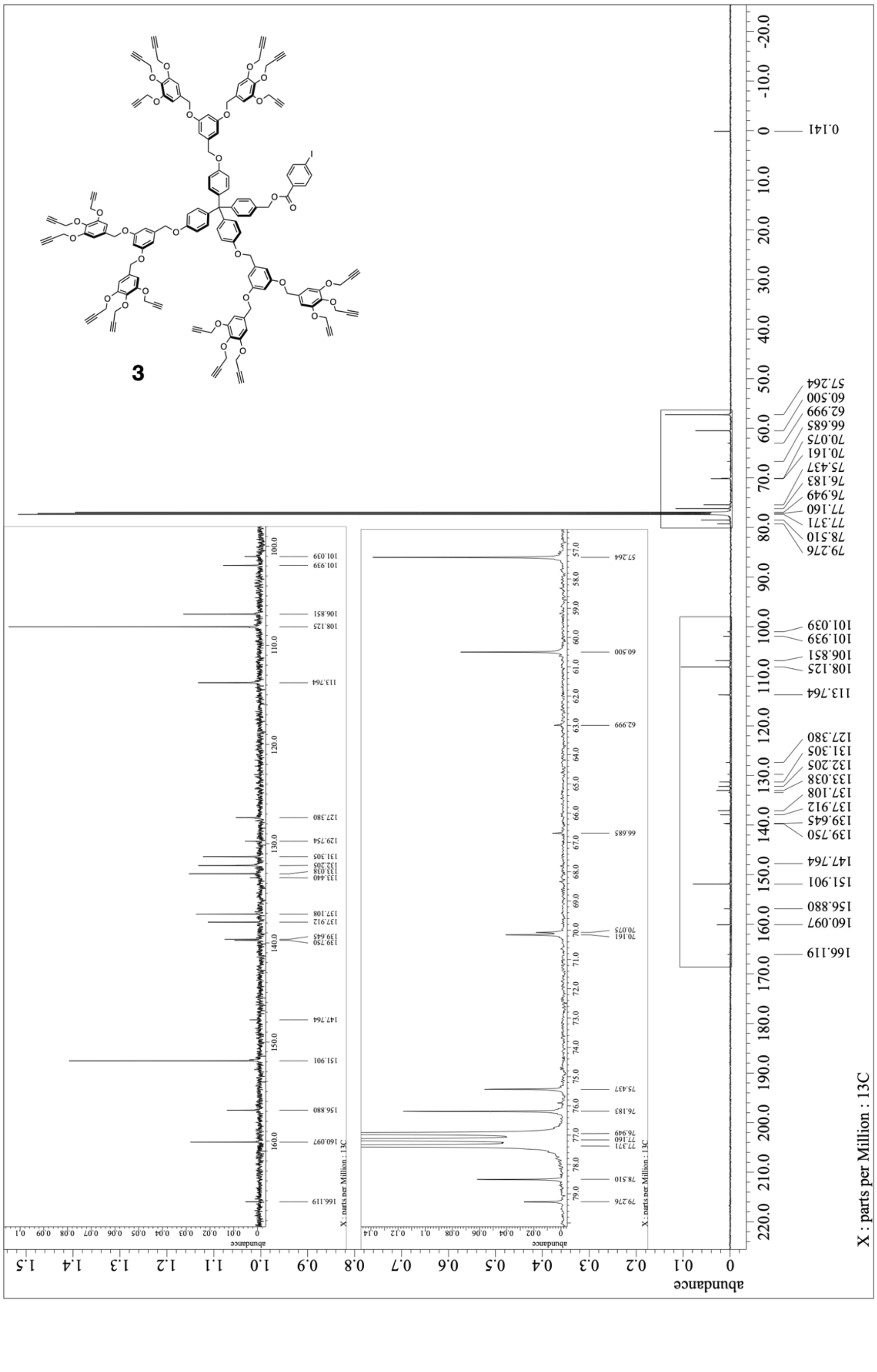

Supplementary Fig. 23. ^{13}C NMR spectrum of **3** ($CDCl_3$, 150 MHz).

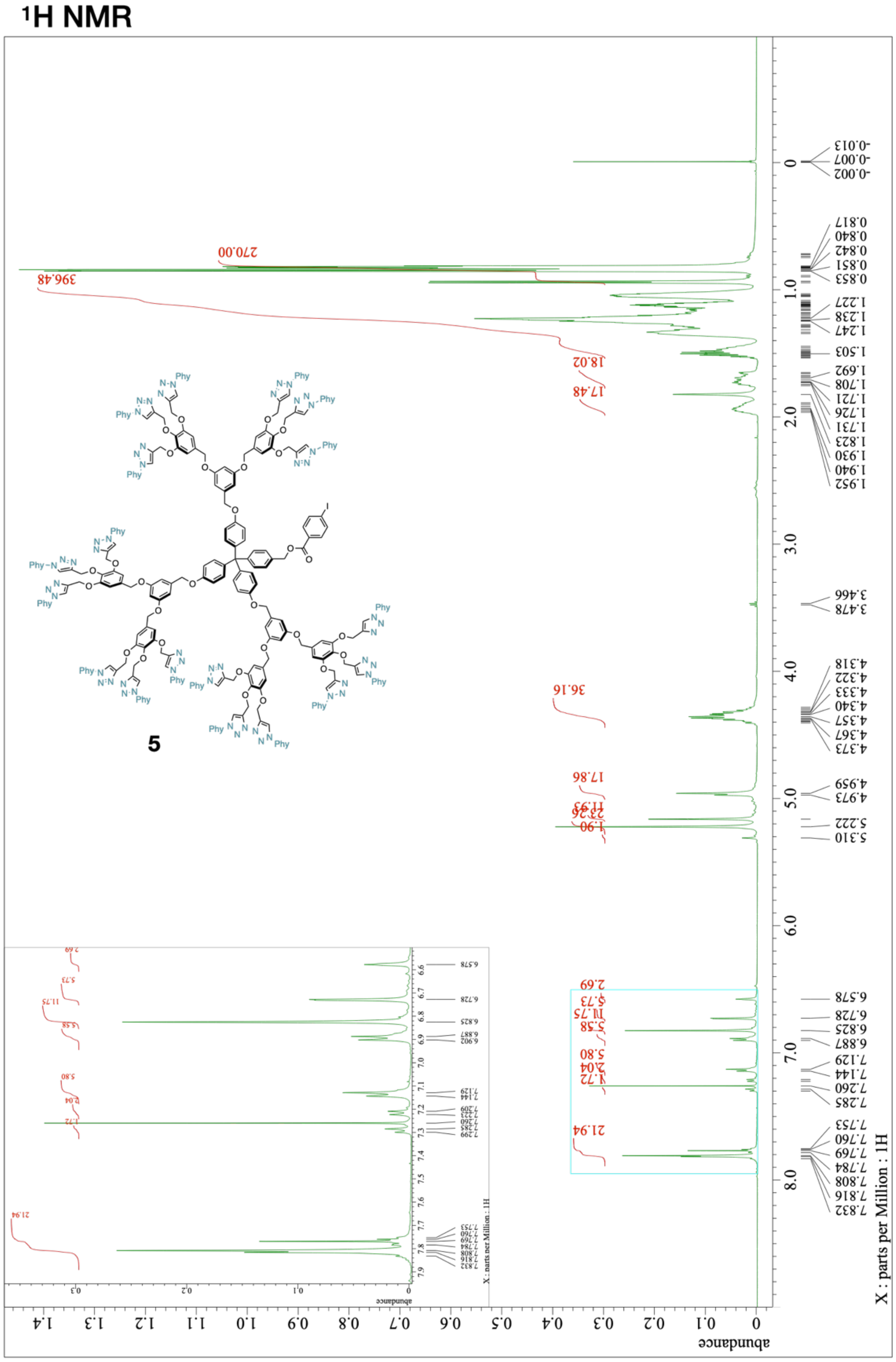

Supplementary Fig. 24. ^{1}H NMR spectrum of **5** ($CDCl_3$, 600 MHz).

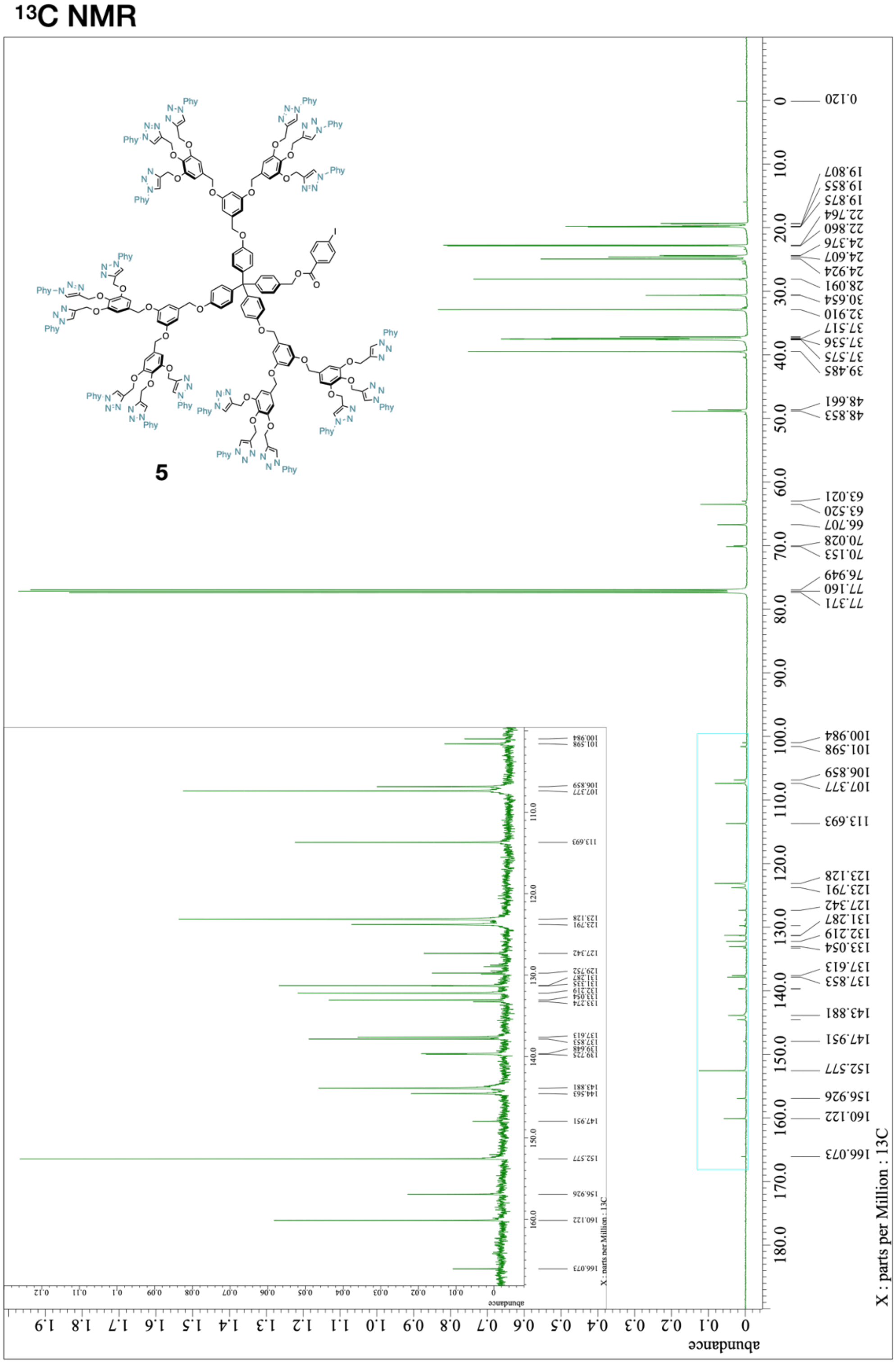

Supplementary Fig. 25. ^{13}C NMR spectrum of **5** ($CDCl_3$, 150 MHz).

^{1}H NMR (full-scale)

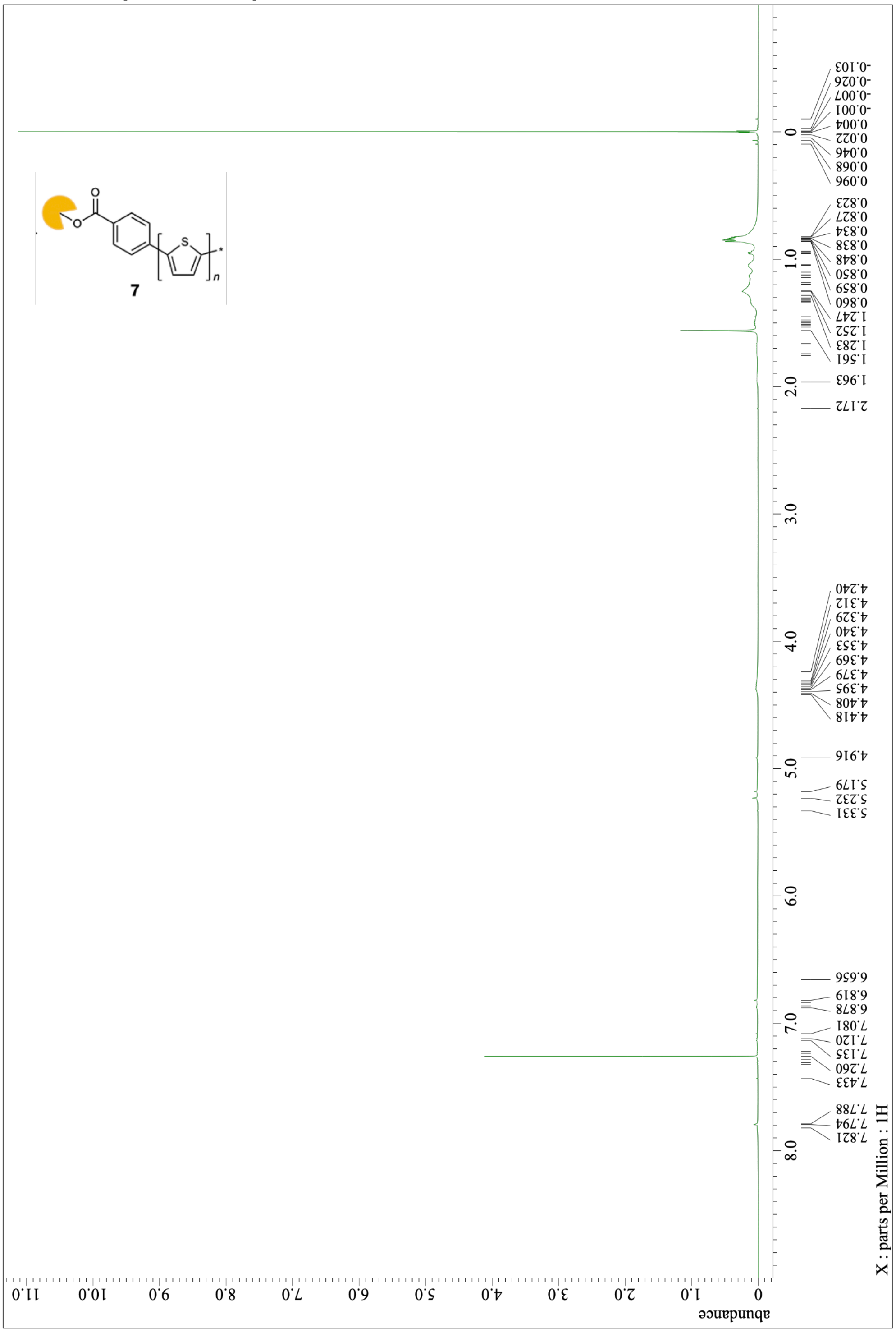

Supplementary Fig. 26. ^{1}H NMR spectrum of **7** ($CDCl_3$, 600 MHz).

^{1}H NMR (enlarged)

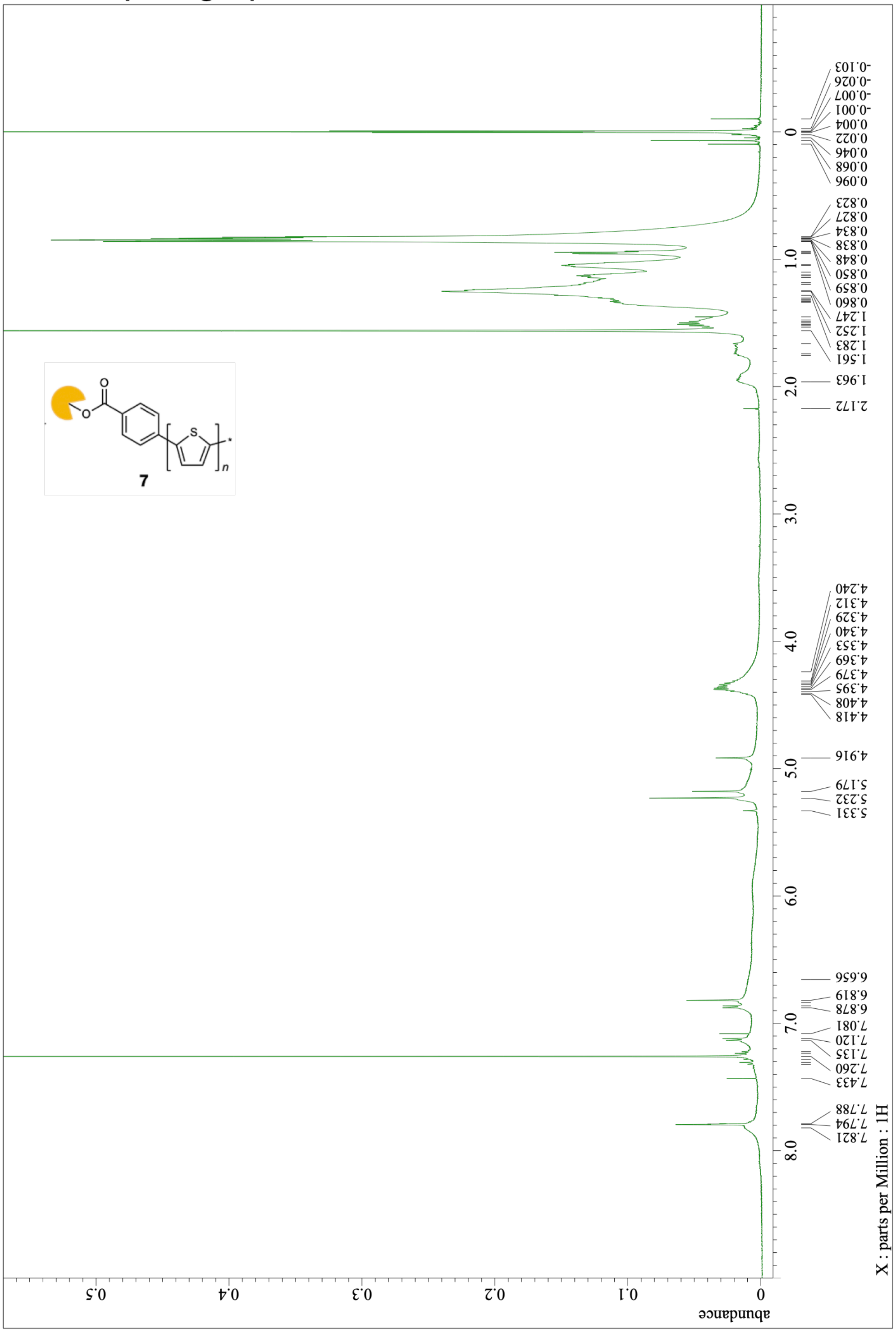

Supplementary Fig. 27. Enlarged ^{1}H NMR spectrum of **7** ($CDCl_3$, 600 MHz).

^{1}H NMR

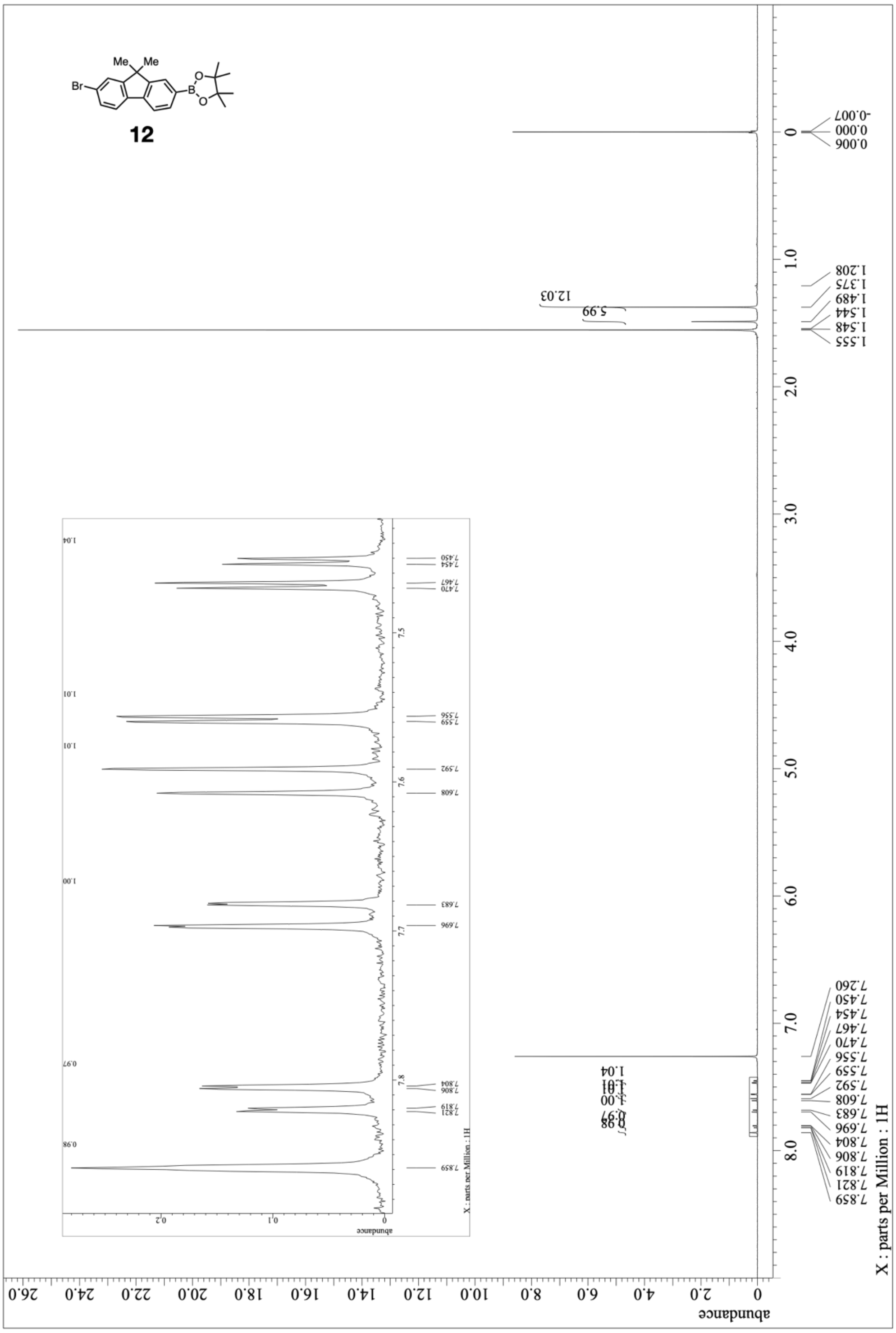

Supplementary Fig. 28. ^{1}H NMR spectrum of **12** ($CDCl_3$, 500 MHz).

^{13}C NMR

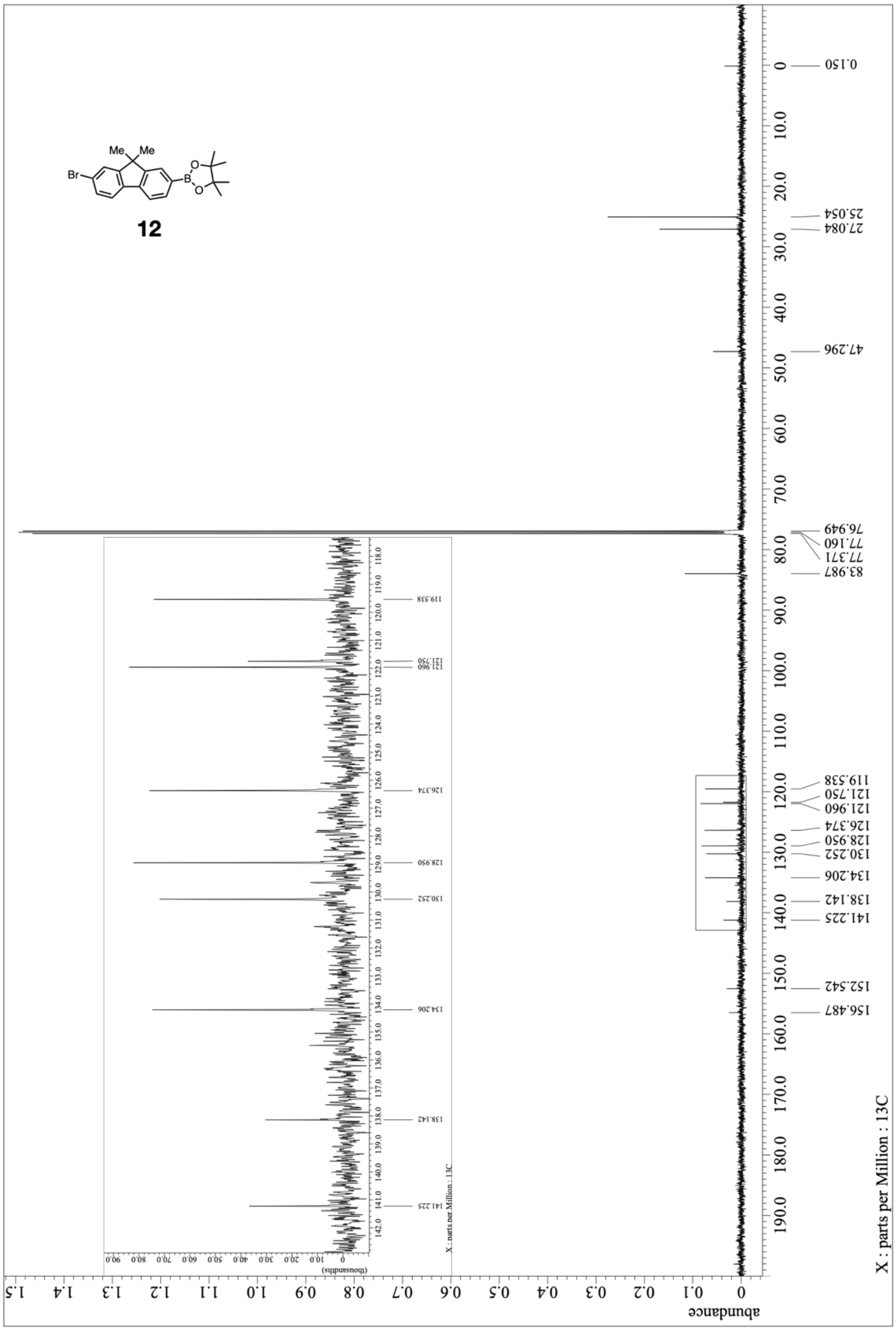

Supplementary Fig. 29. ^{13}C NMR spectrum of **12** ($CDCl_3$, 150 MHz).

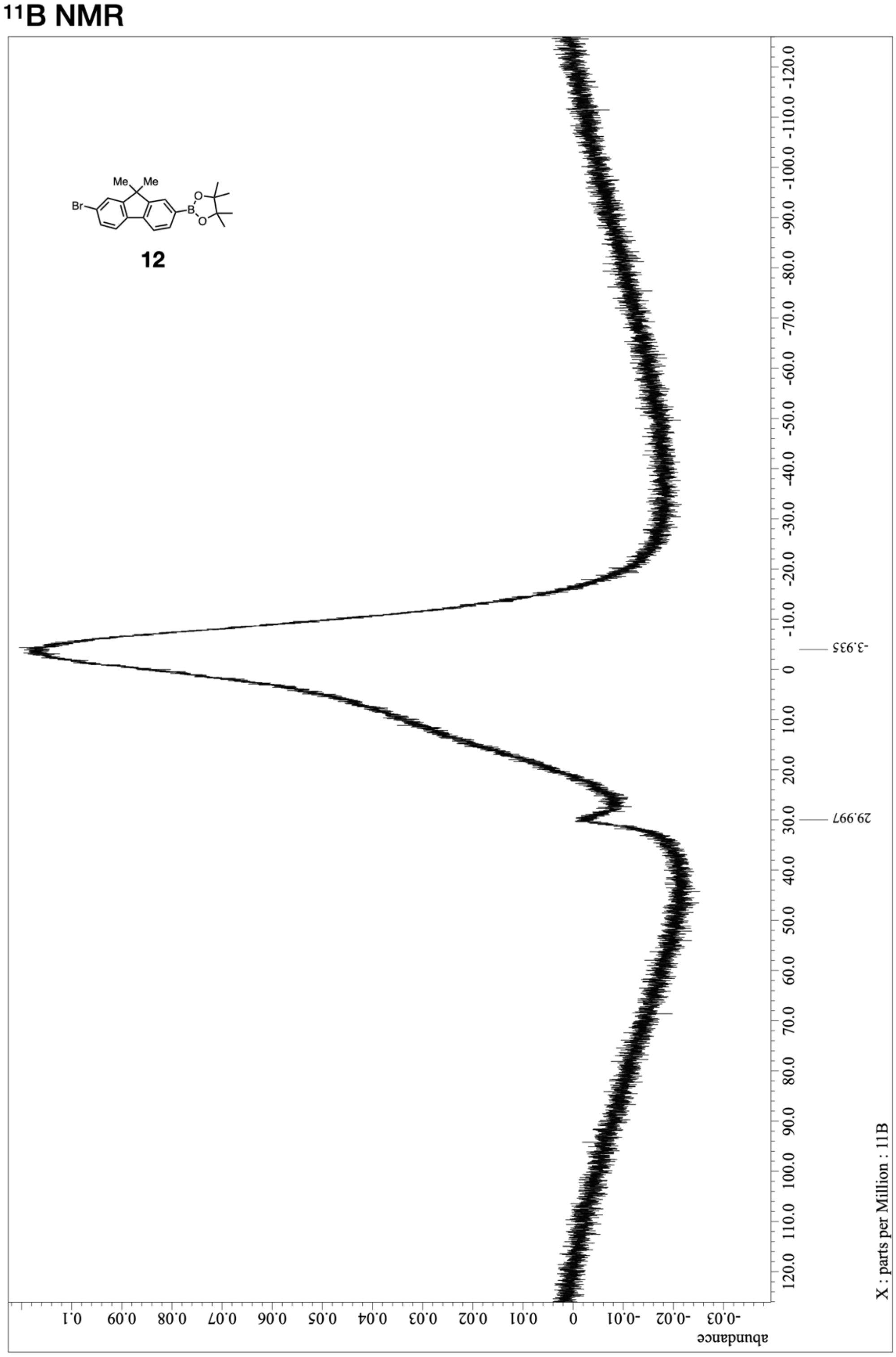

Supplementary Fig. 30. ^{11}B NMR spectrum of **12** ($CDCl_3$, 192 MHz).

¹H NMR

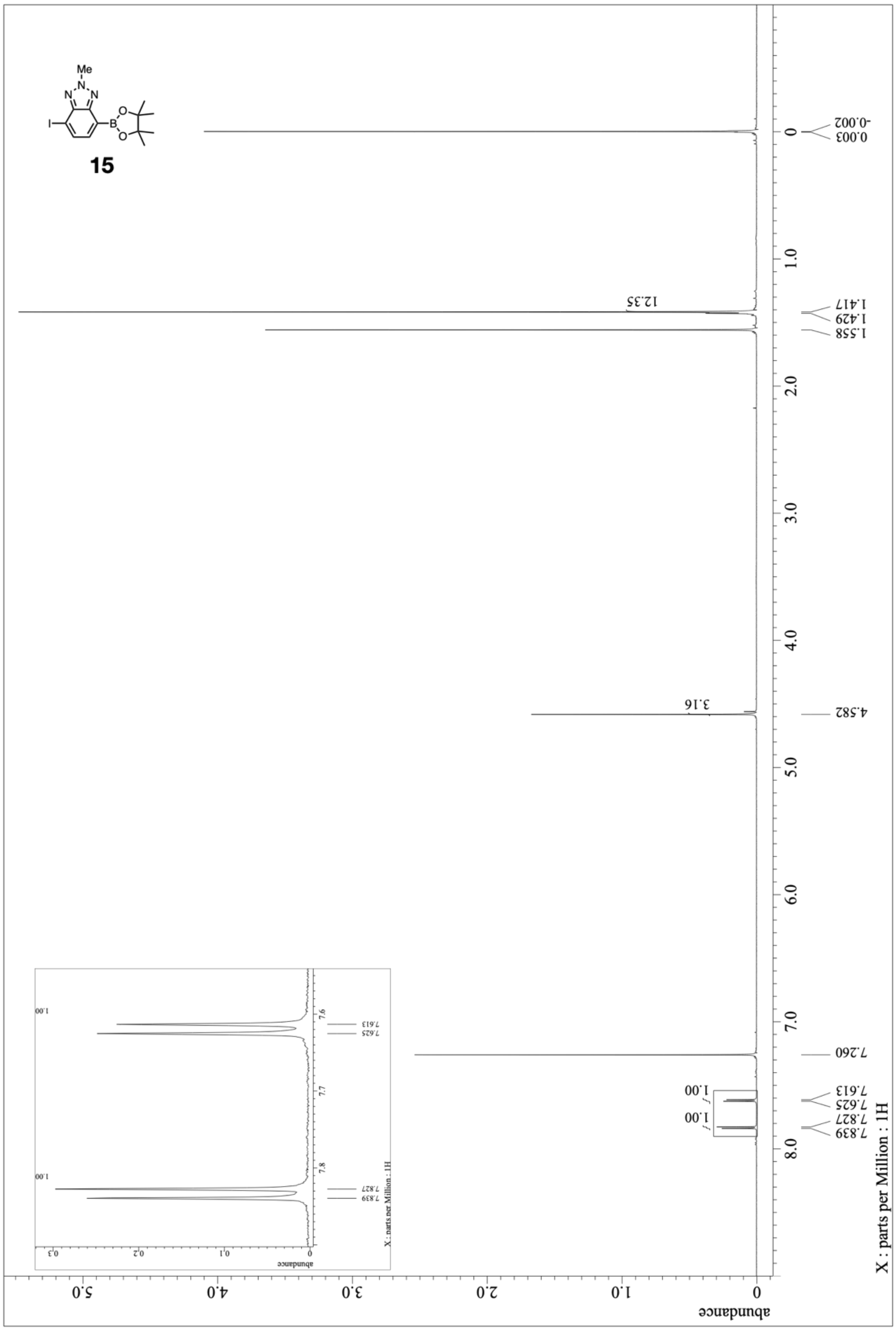

Supplementary Fig. 31. ^{1}H NMR spectrum of **15** ($CDCl_3$, 600 MHz).

^{13}C NMR

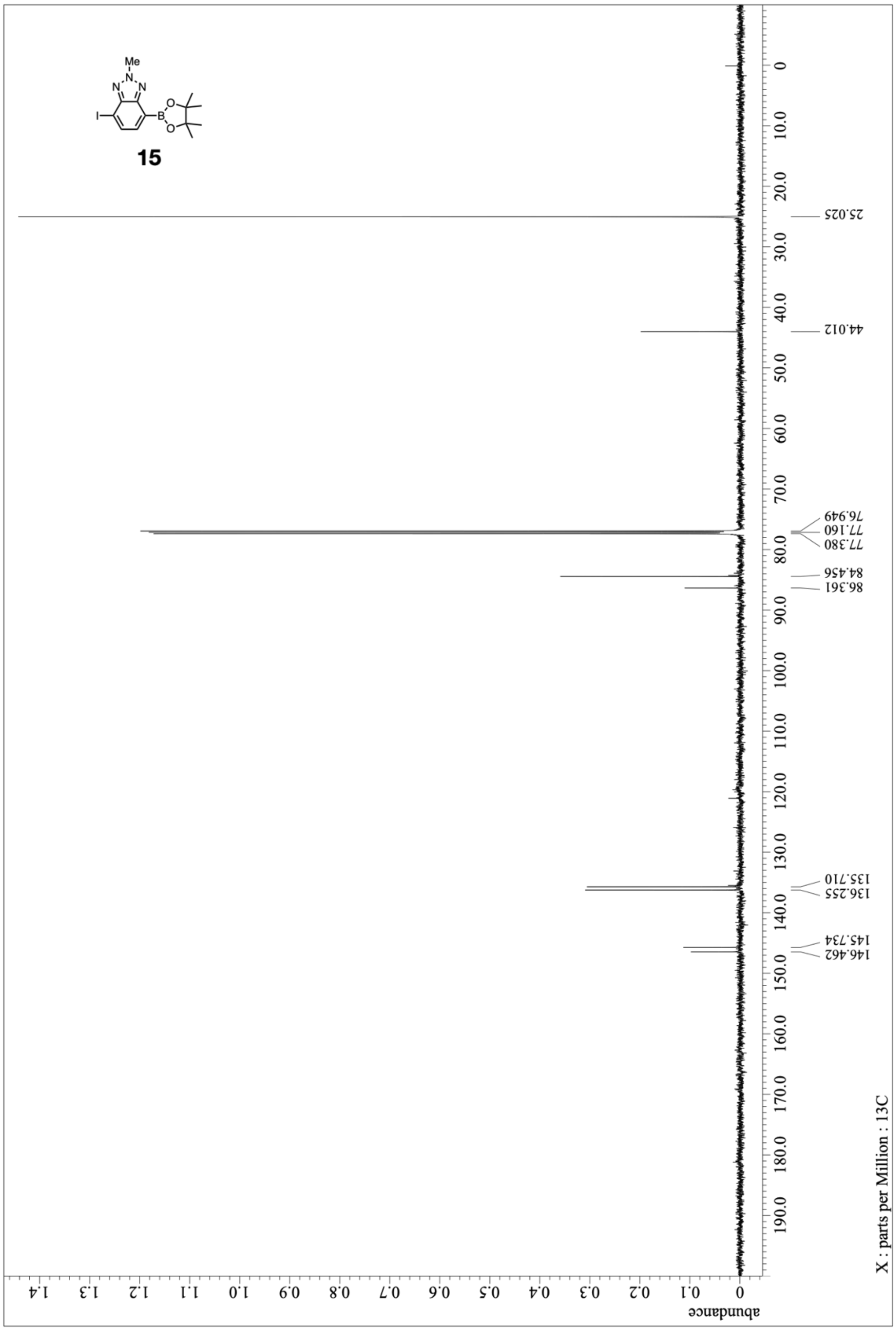

Supplementary Fig. 32. ^{13}C NMR spectrum of **15** ($CDCl_3$, 150 MHz).

^{11}B NMR

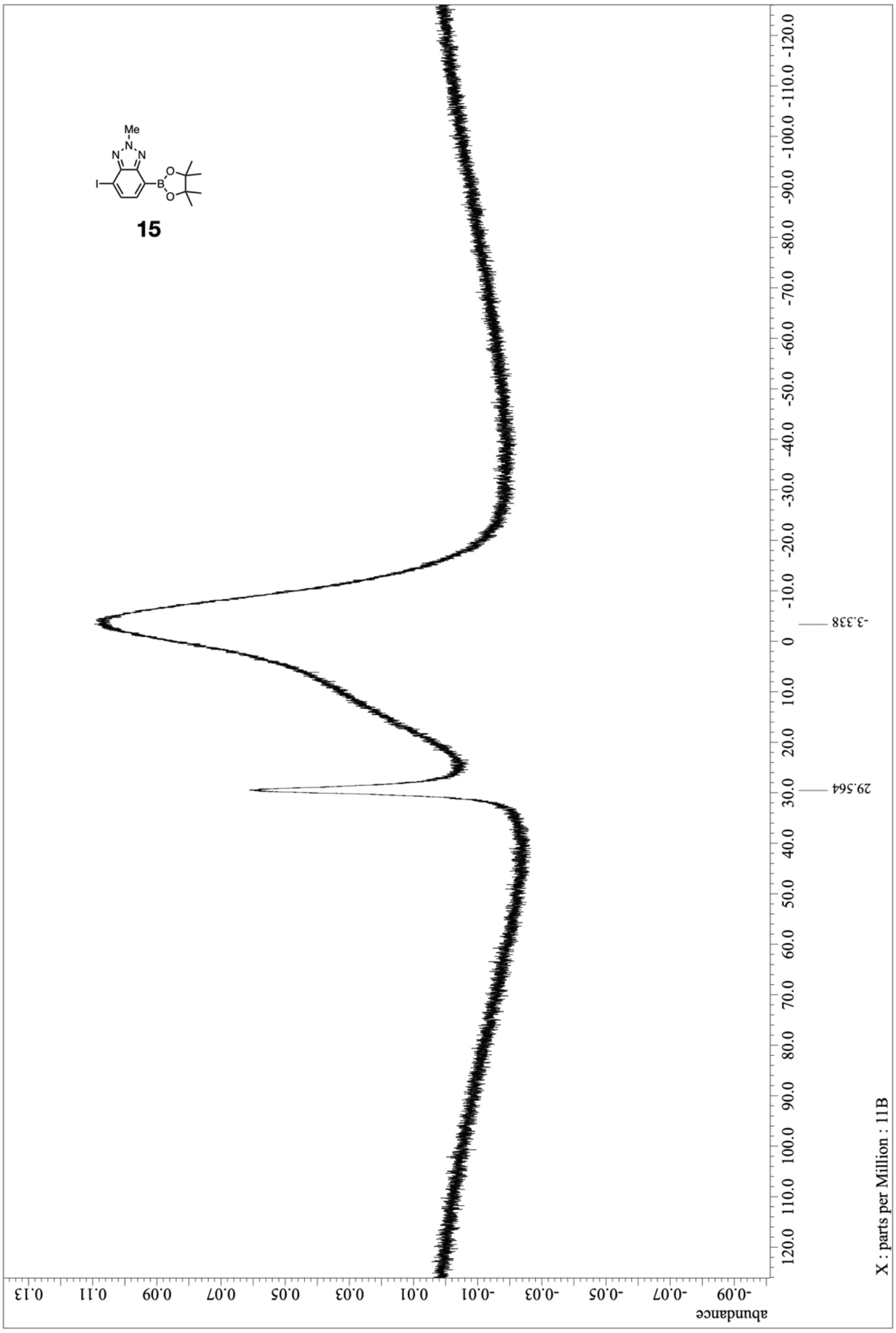

Supplementary Fig. 33. ^{11}B NMR spectrum of **15** ($CDCl_3$, 192 MHz).

¹H NMR

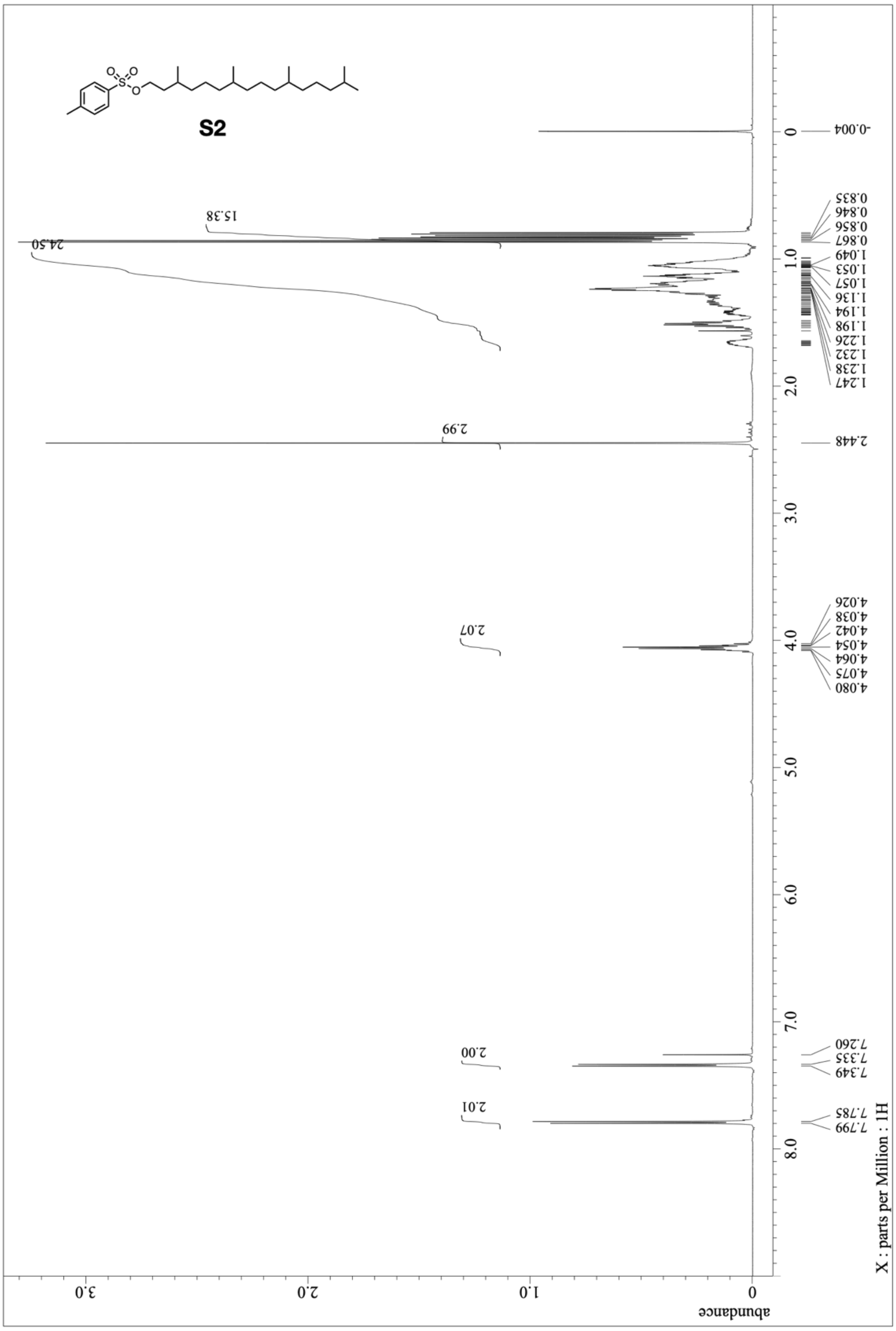

Supplementary Fig. 34. ¹H NMR spectrum of **S2** ($CDCl_3$, 600 MHz).

^{13}C NMR

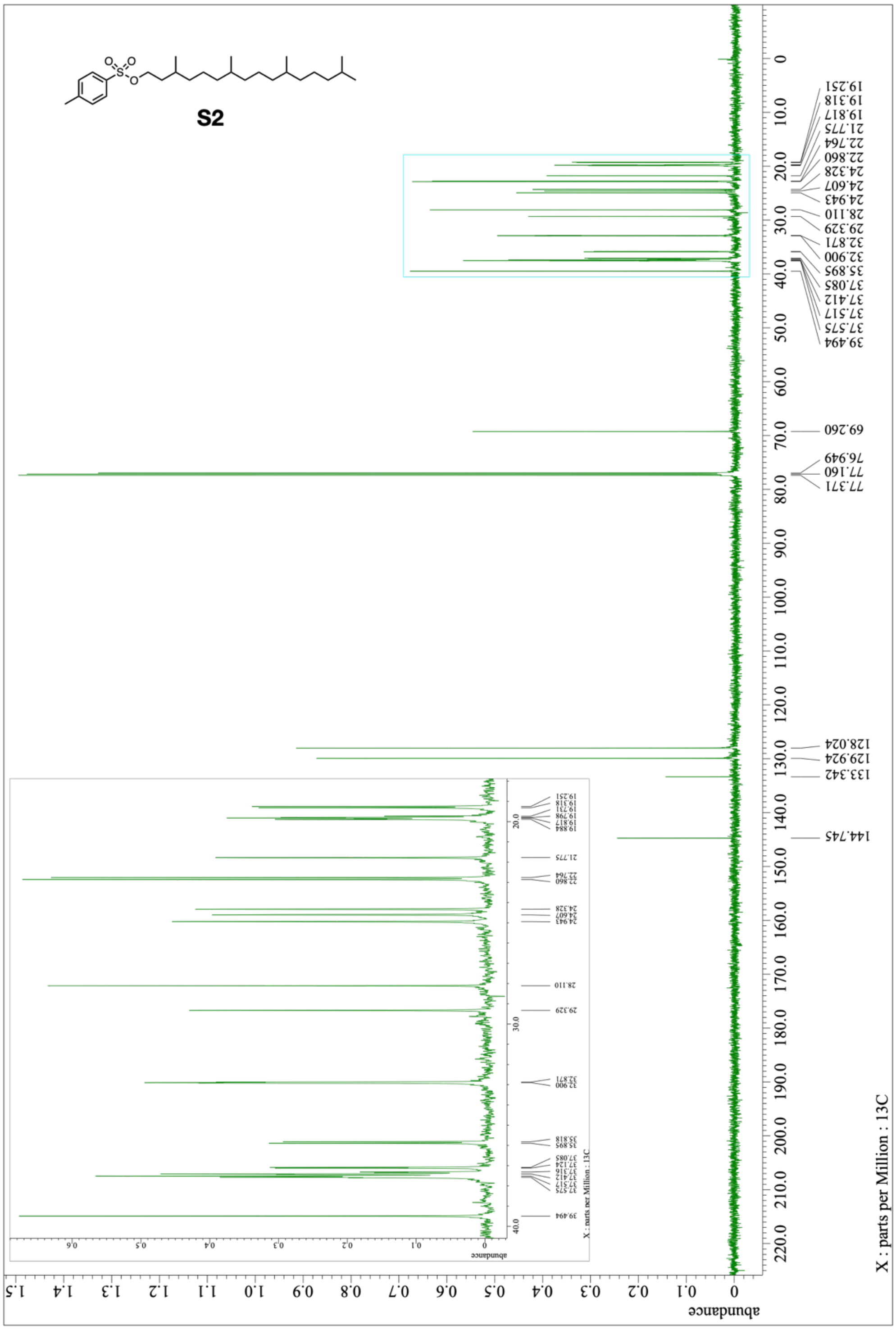

Supplementary Fig. 35. ^{13}C NMR spectrum of **S2** ($CDCl_3$, 150 MHz).

¹H NMR

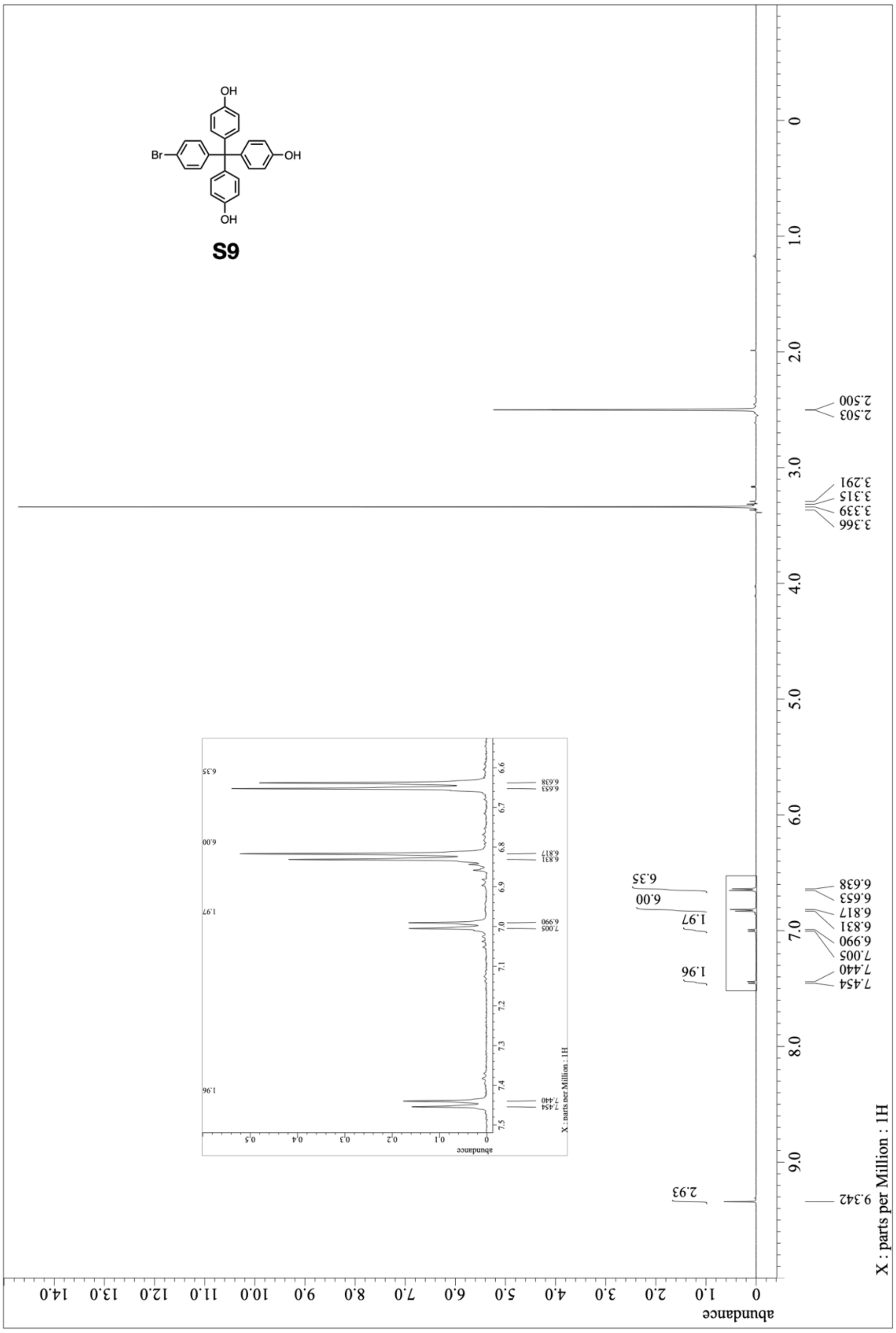

Supplementary Fig. 36. ¹H NMR spectrum of **S9** ($CDCl_3$, 600 MHz).

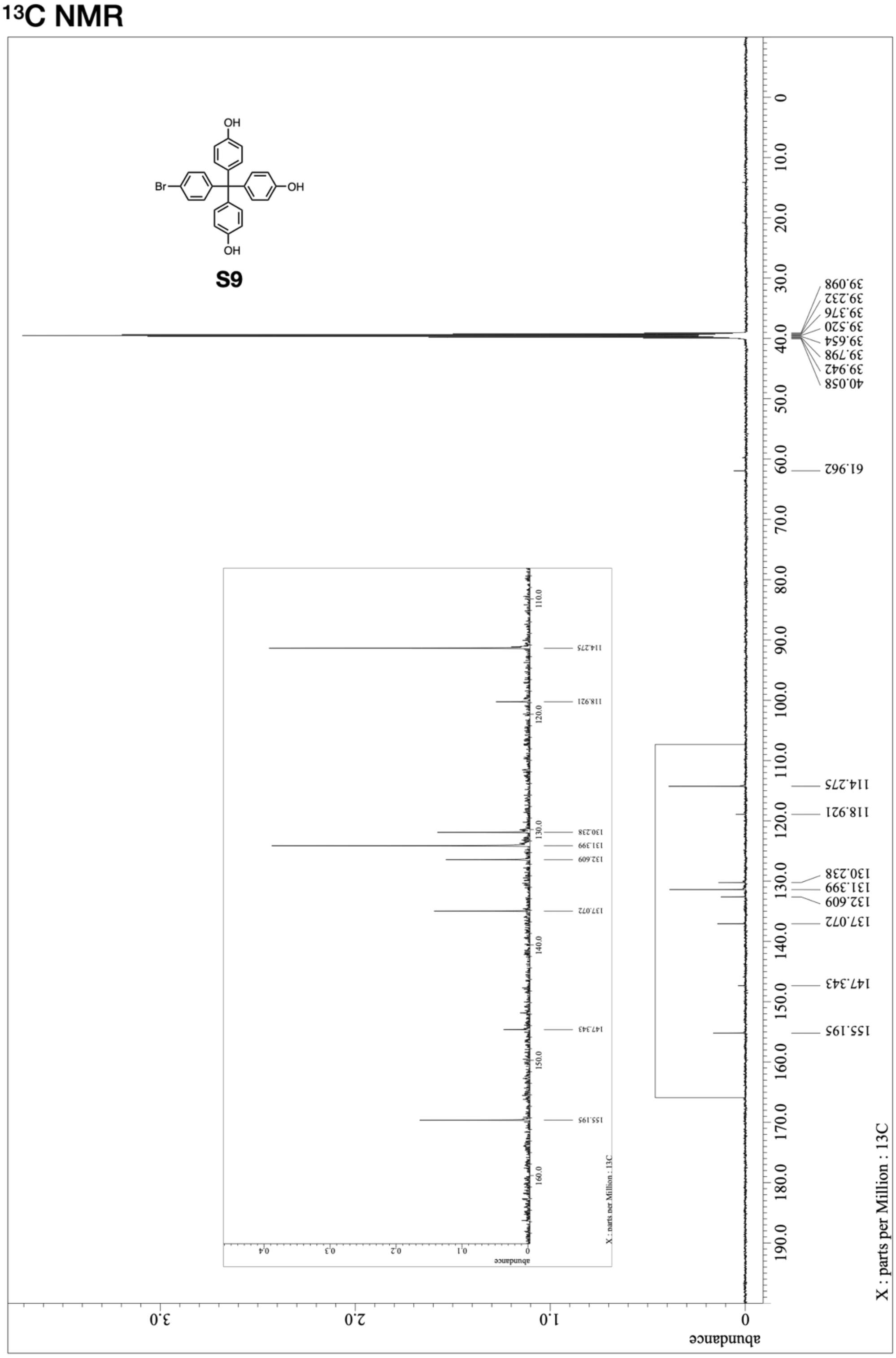

Supplementary Fig. 37. ^{13}C NMR spectrum of **S9** (DMSO-d_6, 150 MHz).

^{1}H NMR

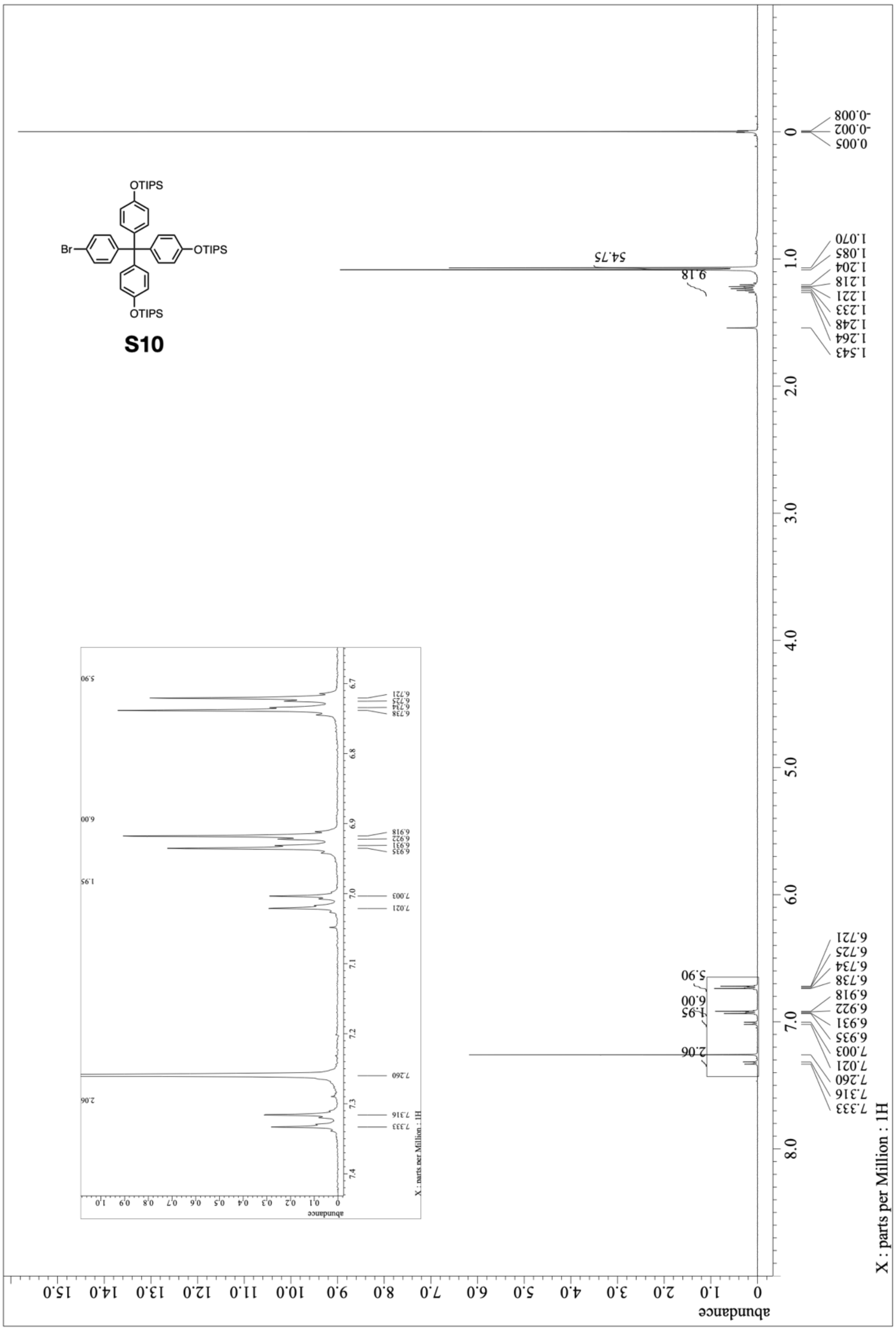

Supplementary Fig. 38. ^{1}H NMR spectrum of **S10** ($CDCl_3$, 500 MHz).

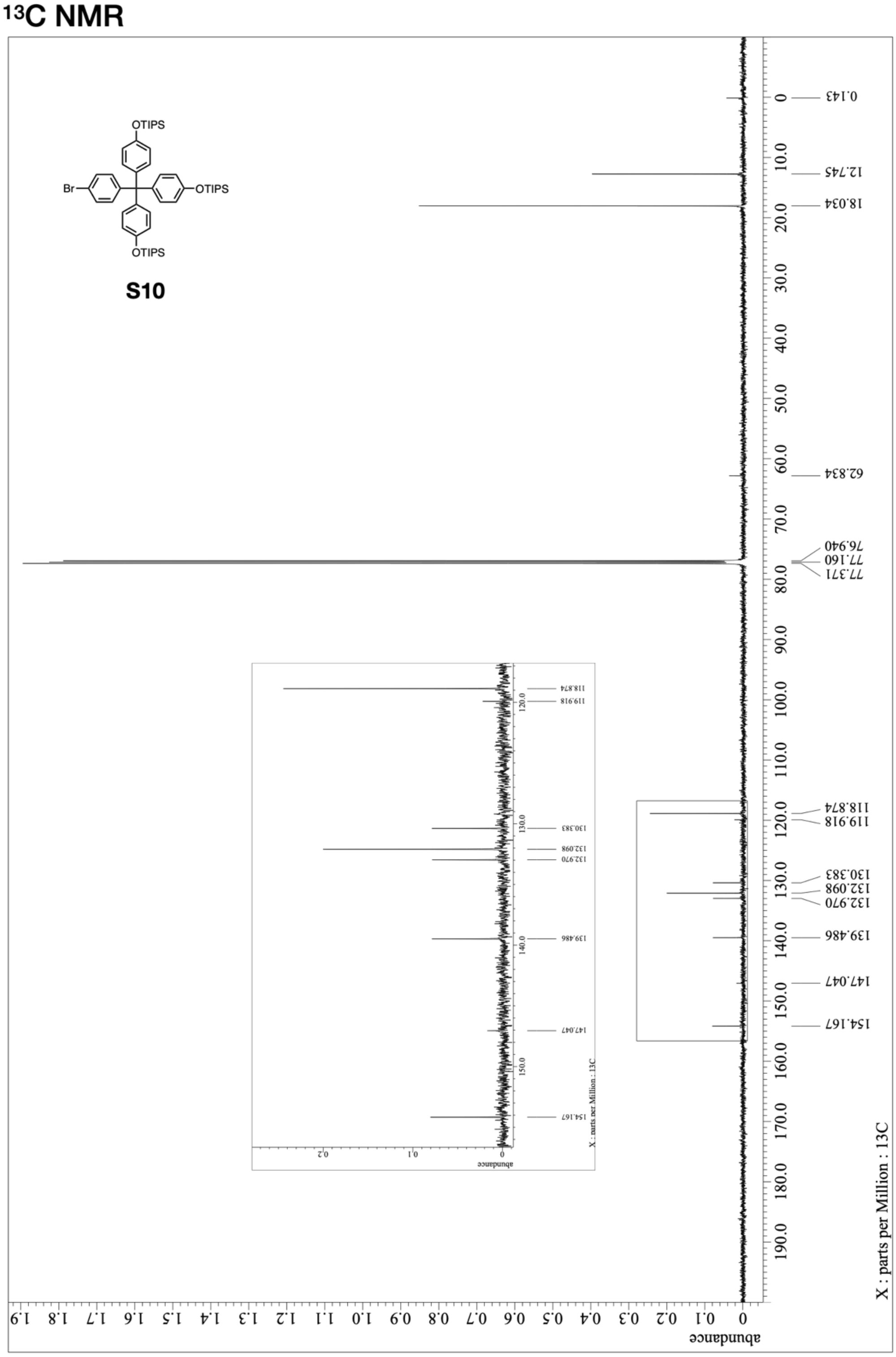

Supplementary Fig. 39. ^{13}C NMR spectrum of **S10** ($CDCl_3$, 150 MHz).

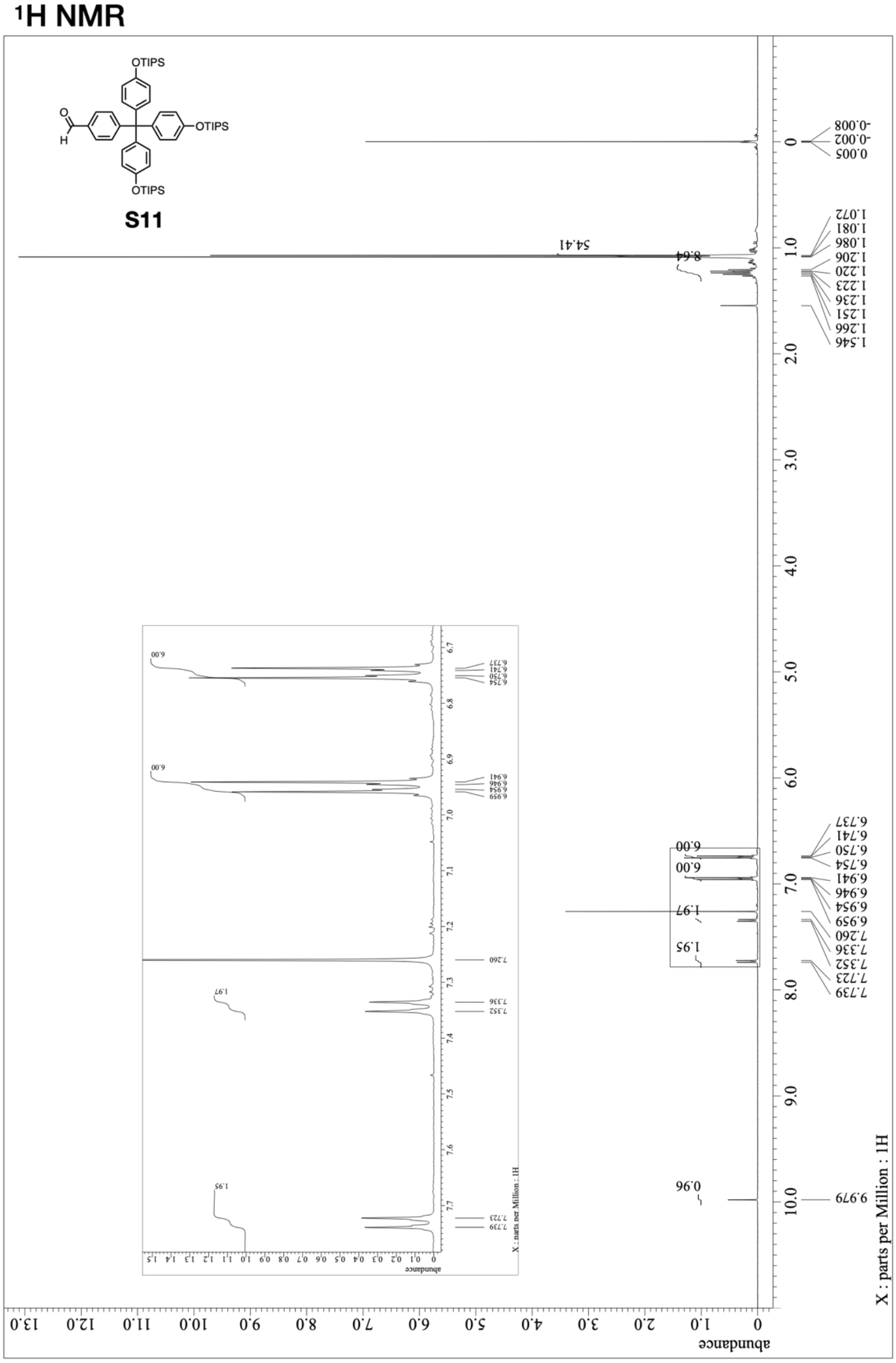

Supplementary Fig. 40. ^{1}H NMR spectrum of **S11** ($CDCl_3$, 500 MHz).

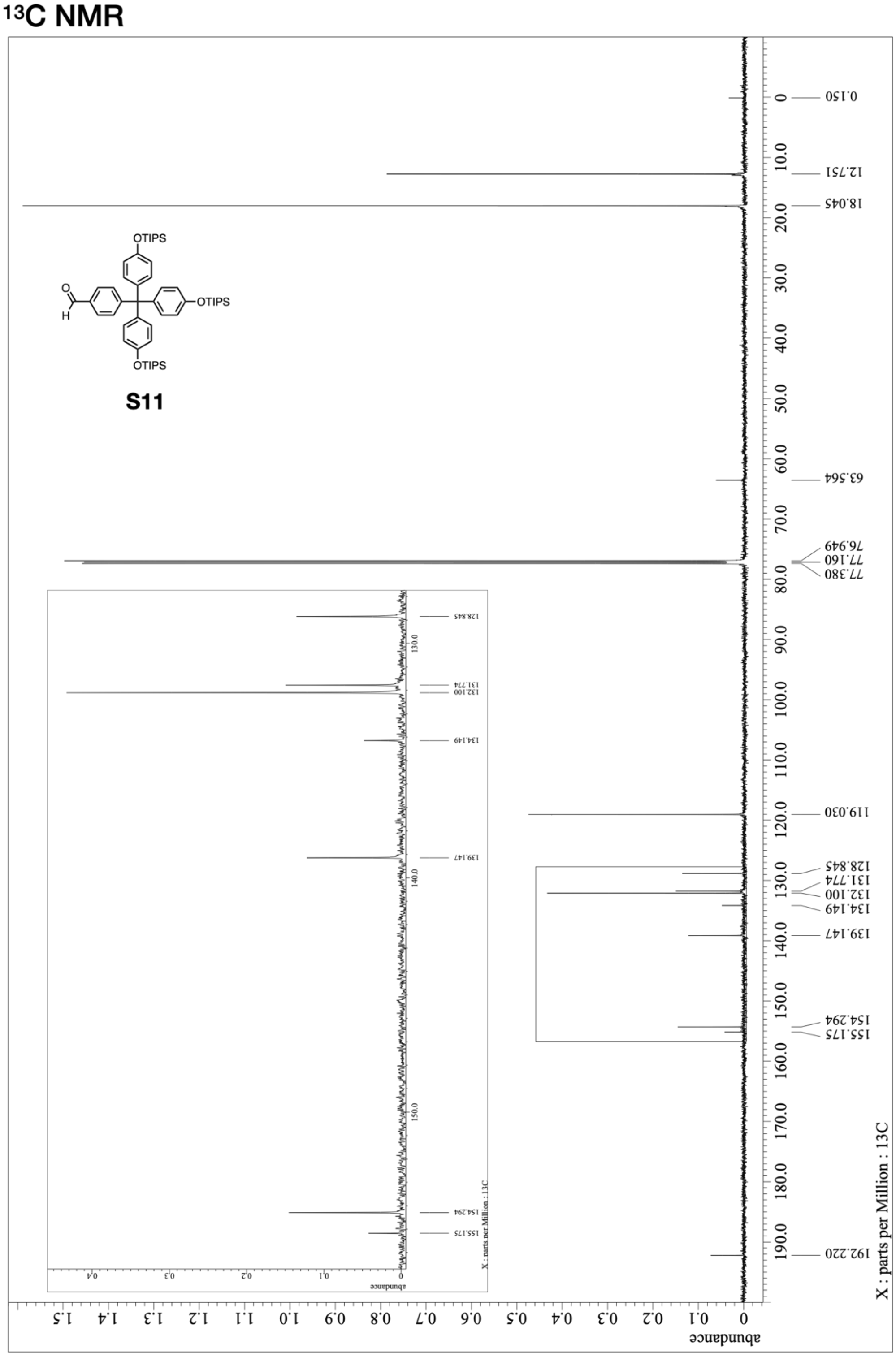

Supplementary Fig. 41. ^{13}C NMR spectrum of **S11** ($CDCl_3$, 150 MHz).

^{1}H NMR

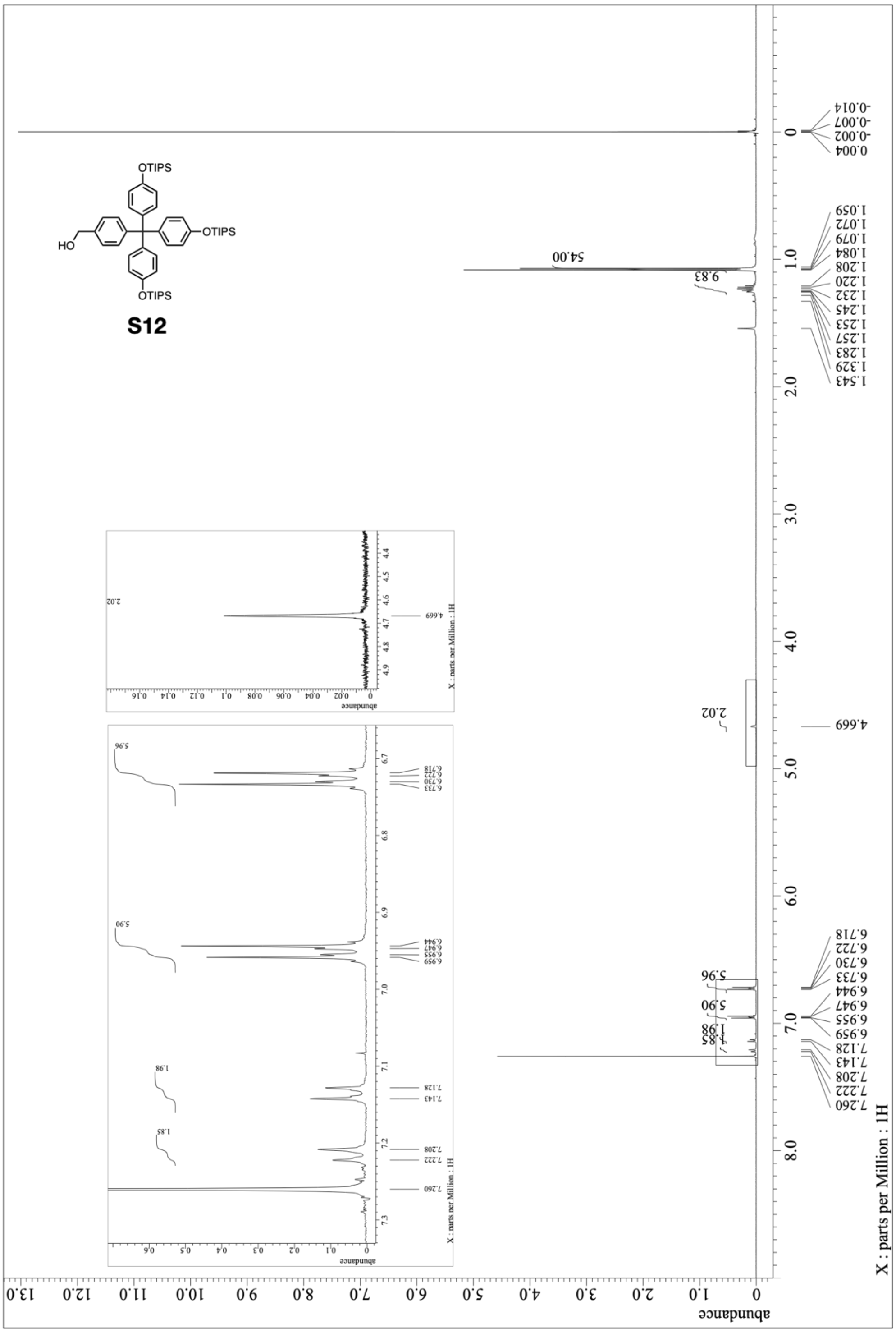

Supplementary Fig. 42. ^{1}H NMR spectrum of **S12** ($CDCl_3$, 600 MHz).

13C NMR

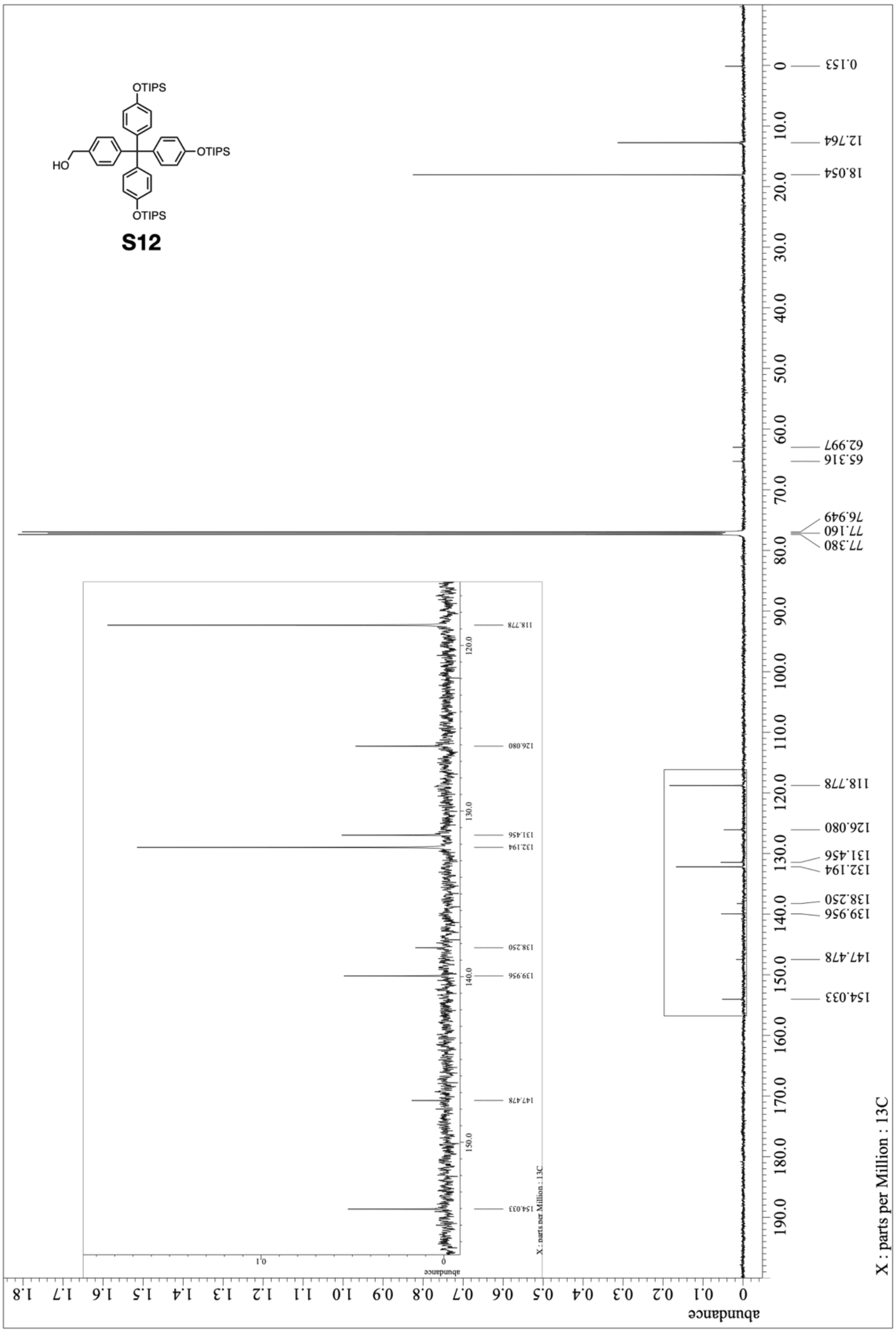

Supplementary Fig. 43. ^{13}C NMR spectrum of **S12** ($CDCl_3$, 150 MHz).

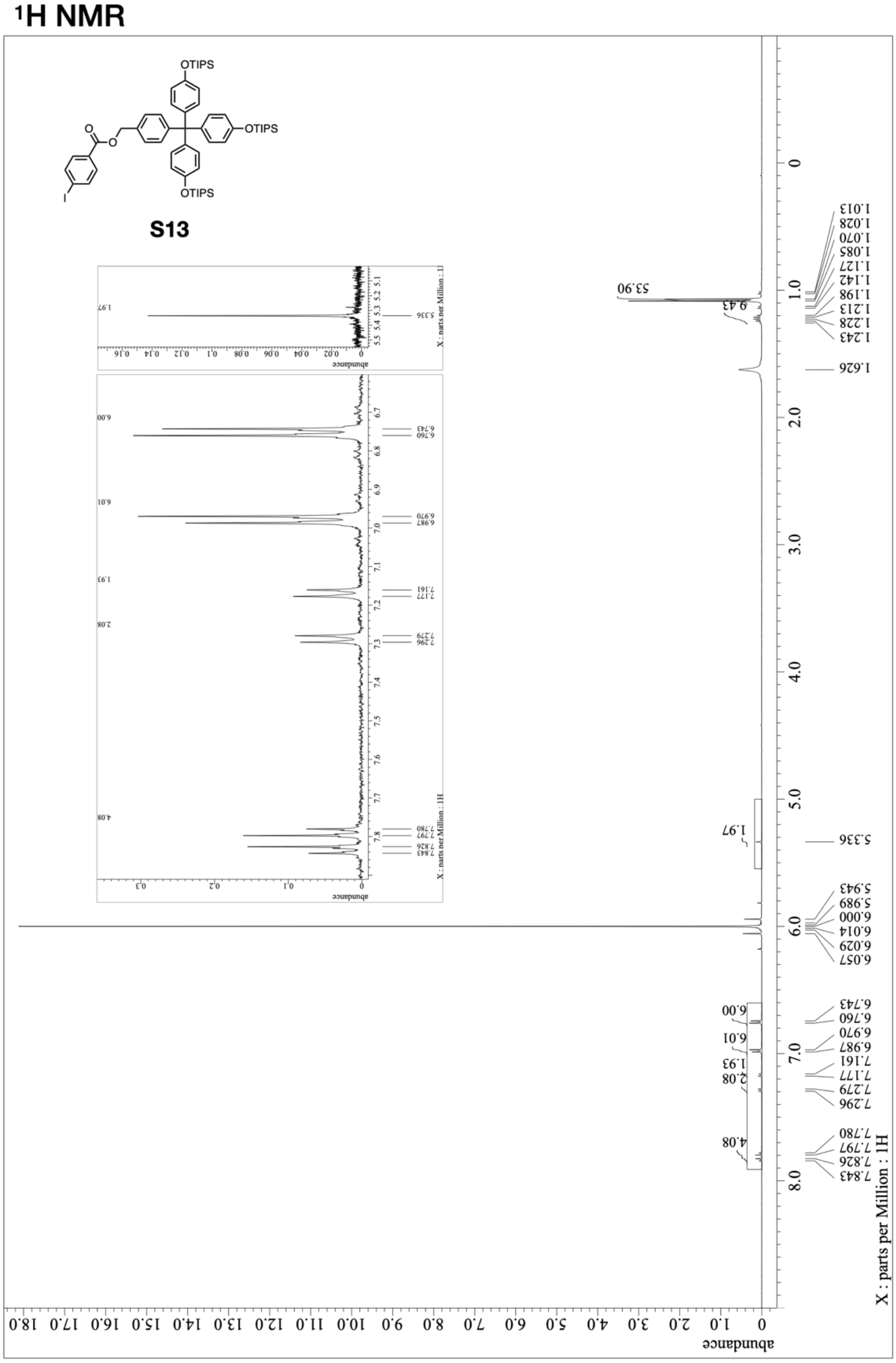

Supplementary Fig. 44. ^{1}H NMR spectrum of **S13** (TCE-d_2, 500 MHz).

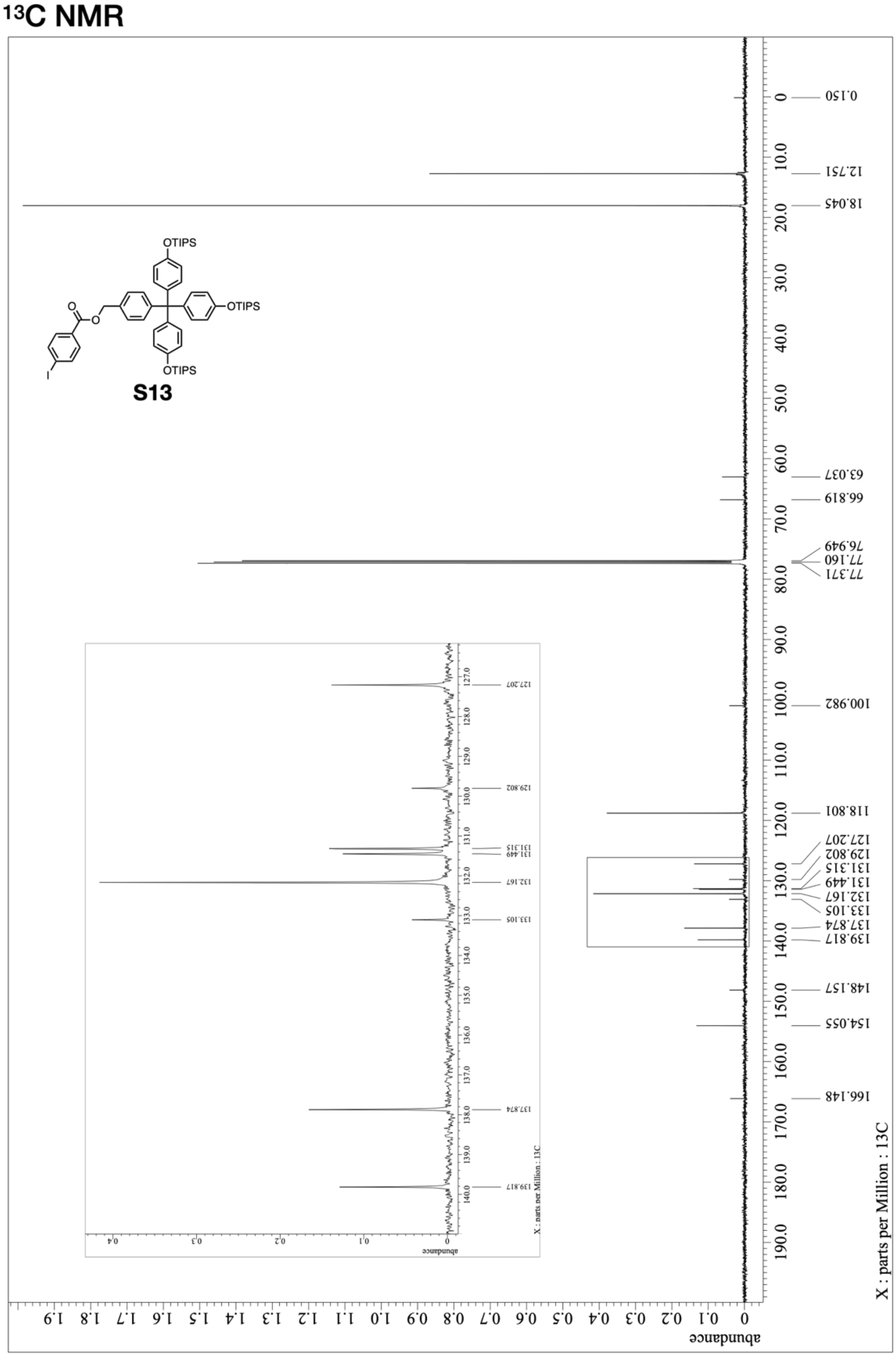

Supplementary Fig. 45. ^{13}C NMR spectrum of **S13** ($CDCl_3$, 150 MHz).

¹H NMR

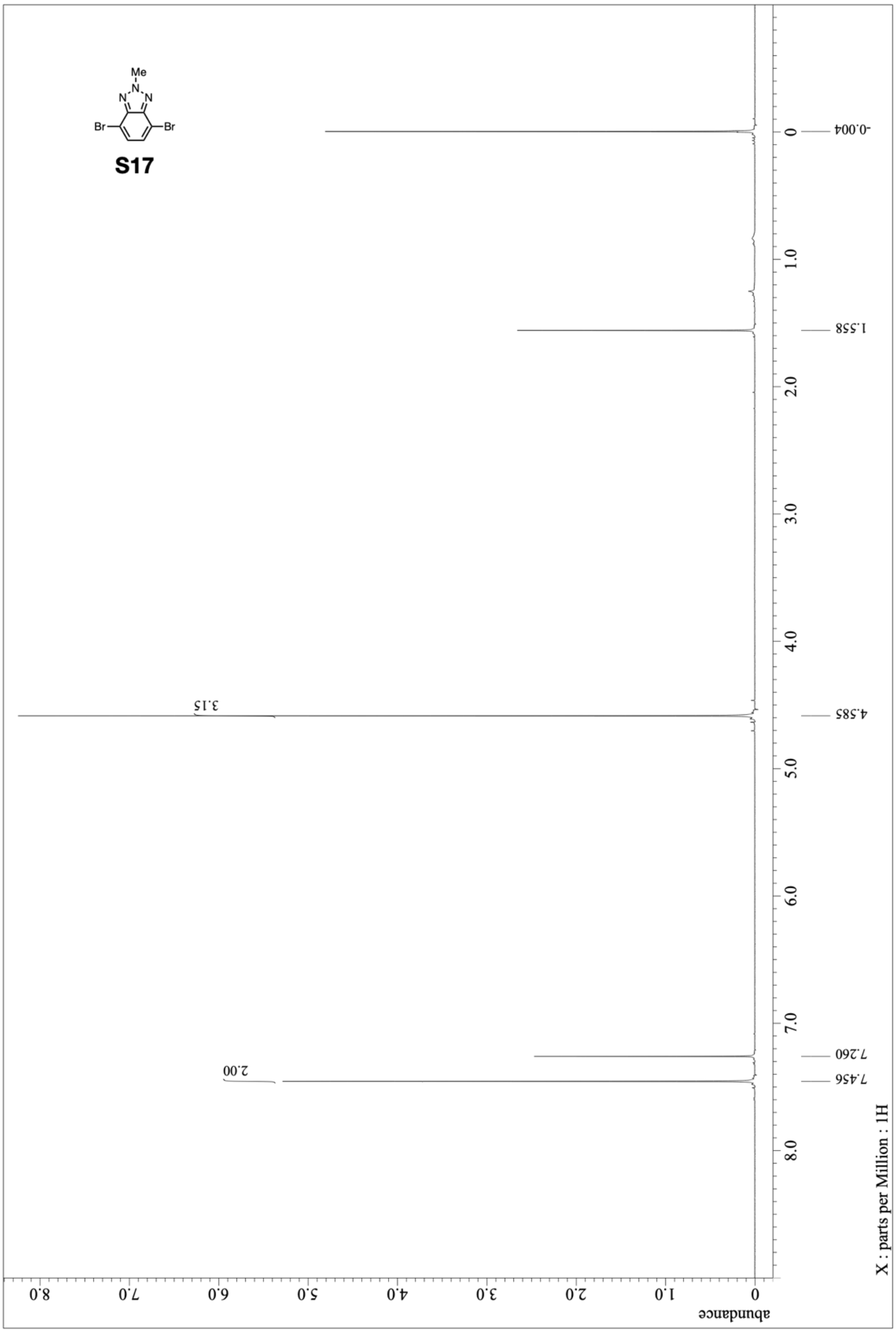

Supplementary Fig. 46. ^{1}H NMR spectrum of **S17** ($CDCl_3$, 600 MHz).

^{13}C NMR

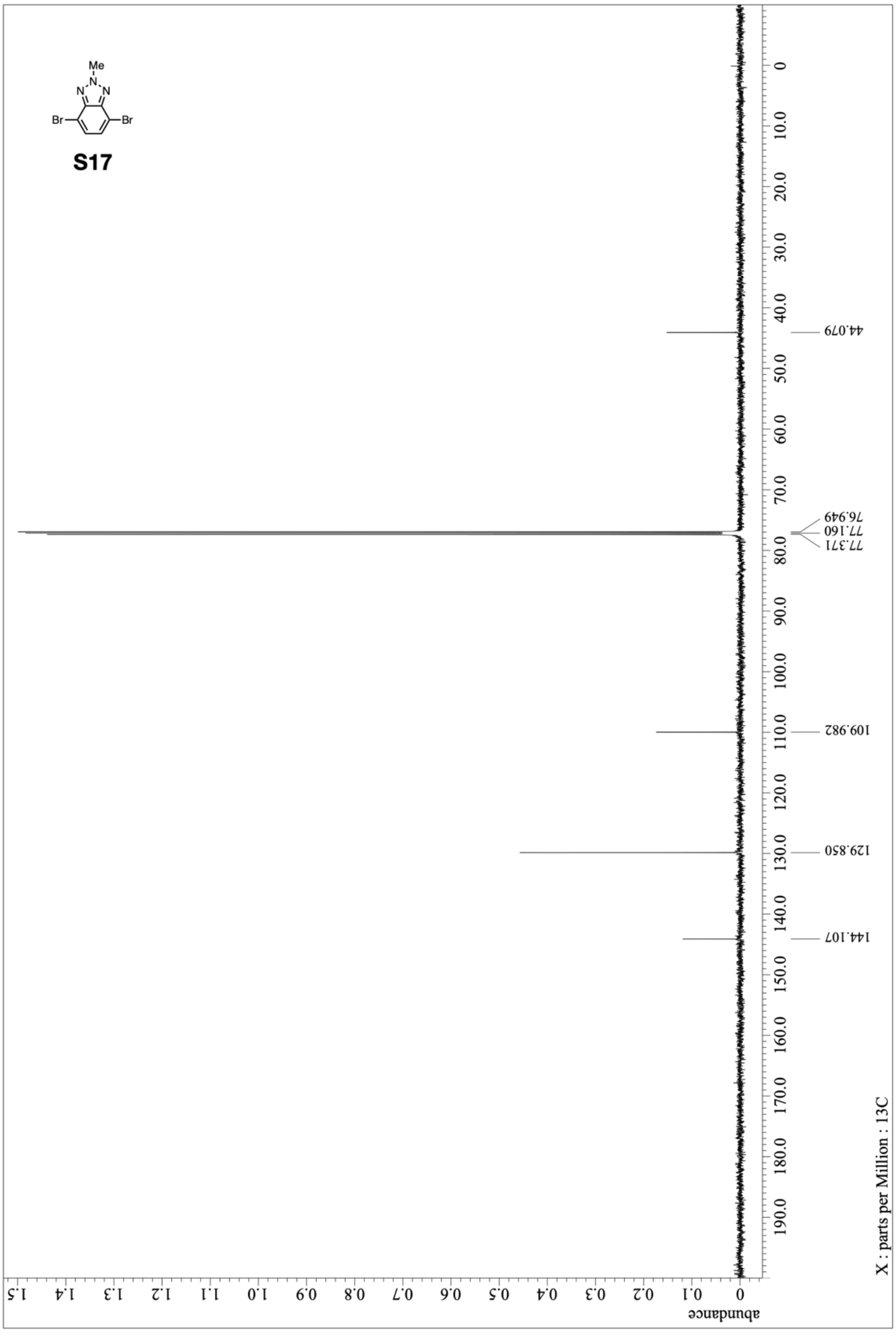

Supplementary Fig. 47. ^{13}C NMR spectrum of **S17** ($CDCl_3$, 150 MHz).

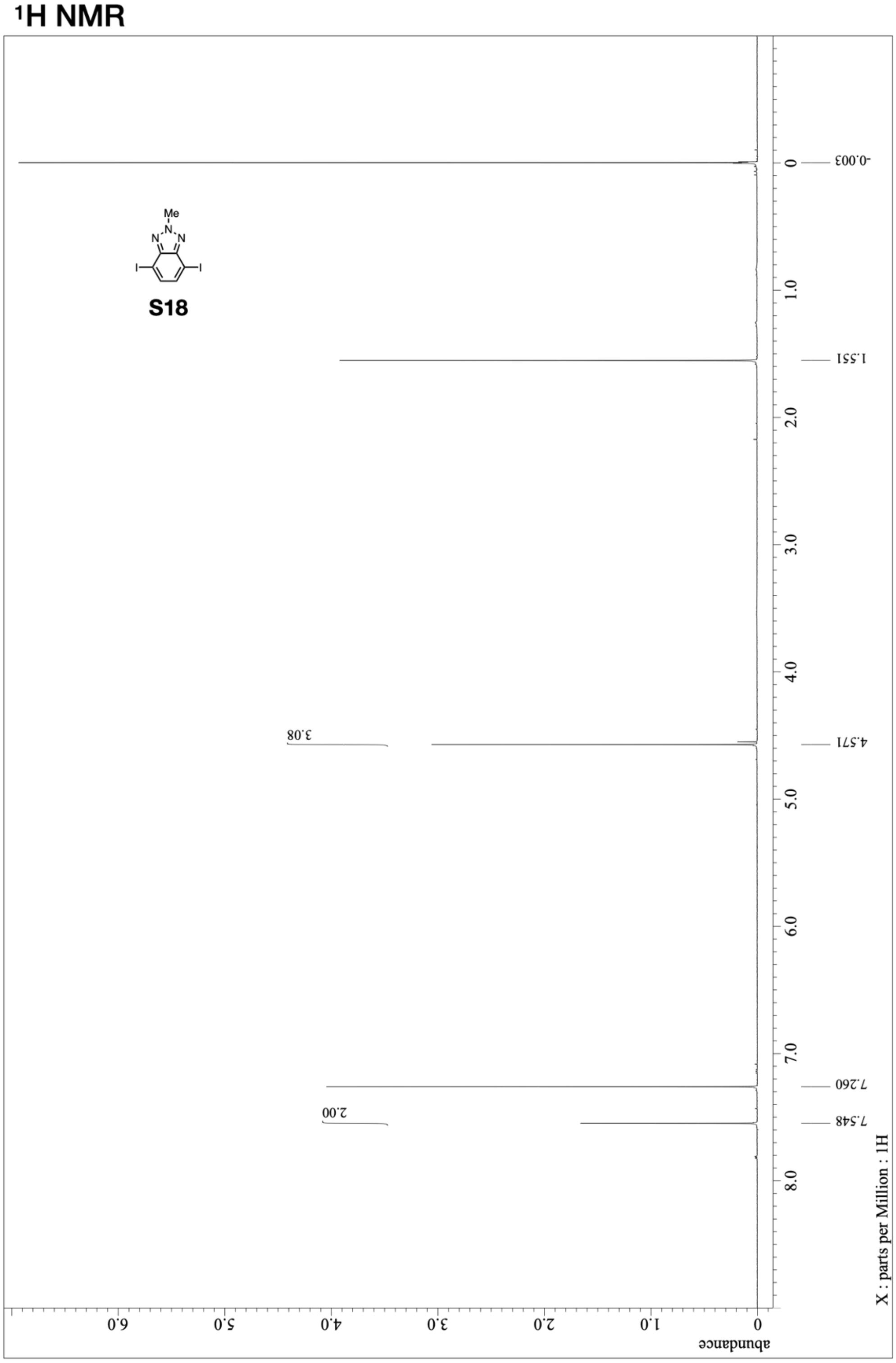

Supplementary Fig. 48. ^{1}H NMR spectrum of **S18** ($CDCl_3$, 600 MHz).

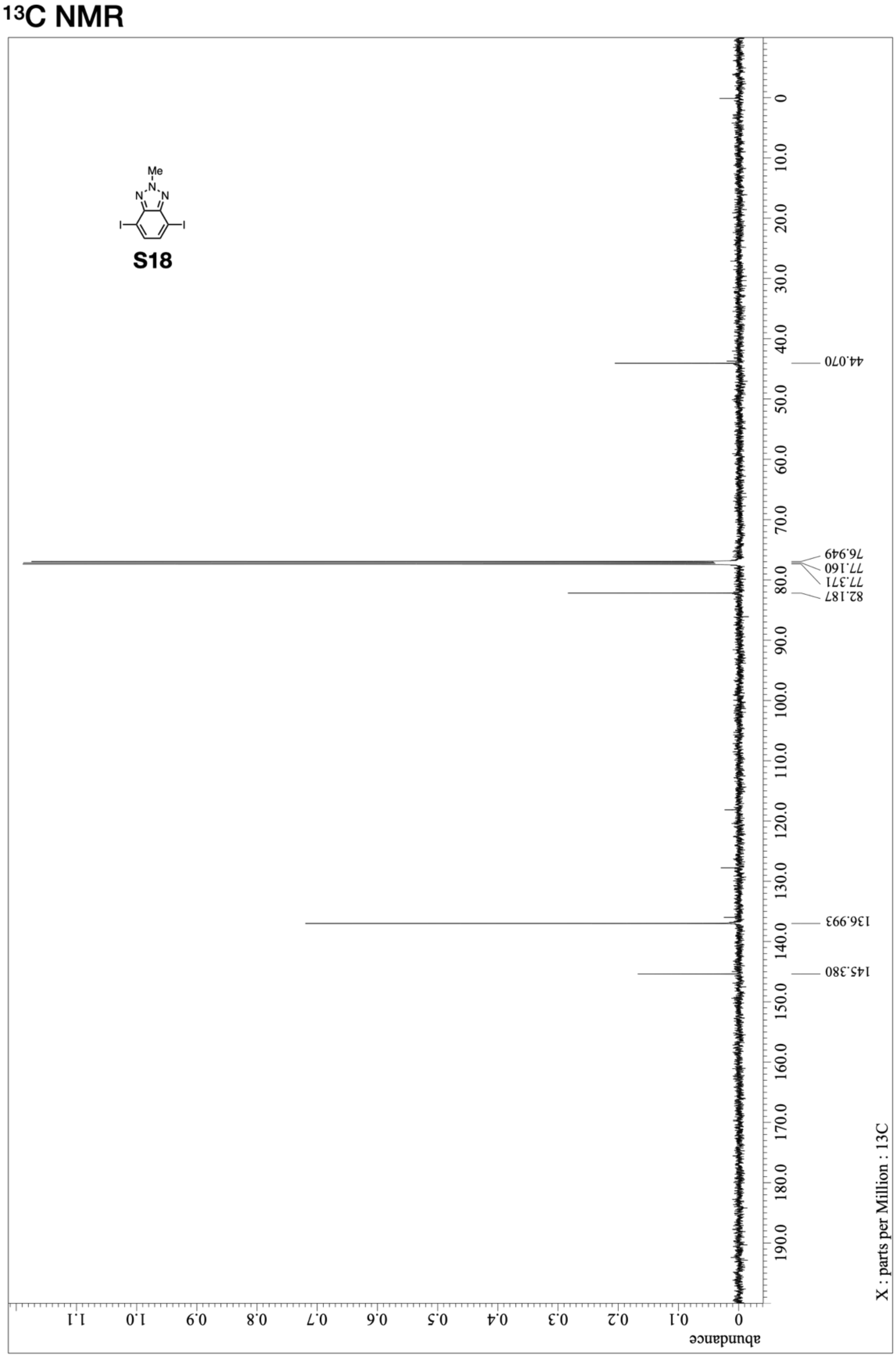

Supplementary Fig. 49. ^{13}C NMR spectrum of **S18** ($CDCl_3$, 150 MHz).

^{1}H NMR

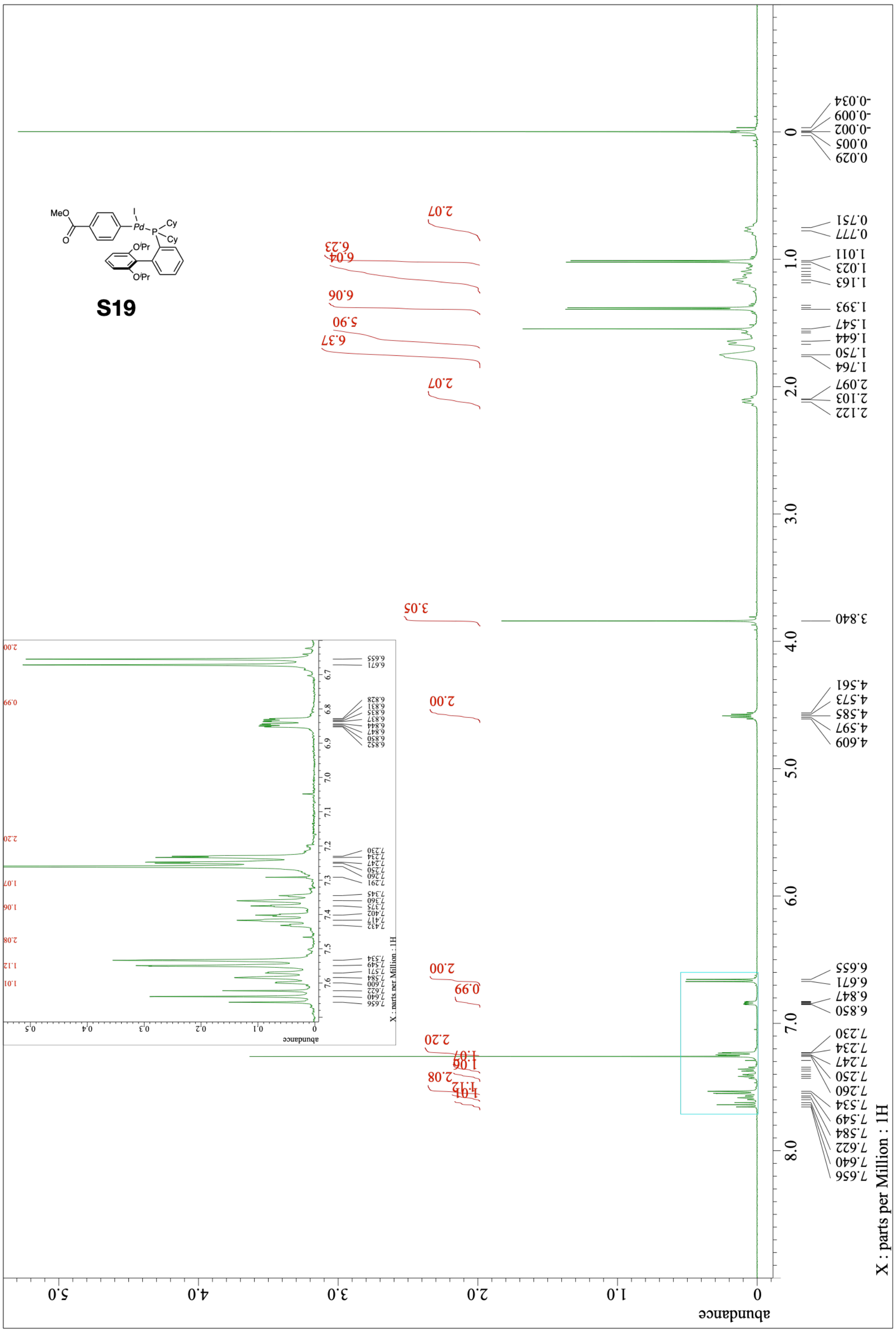

Supplementary Fig. 50. ^{1}H NMR spectrum of **S19** ($CDCl_3$, 500 MHz).

^{13}C NMR

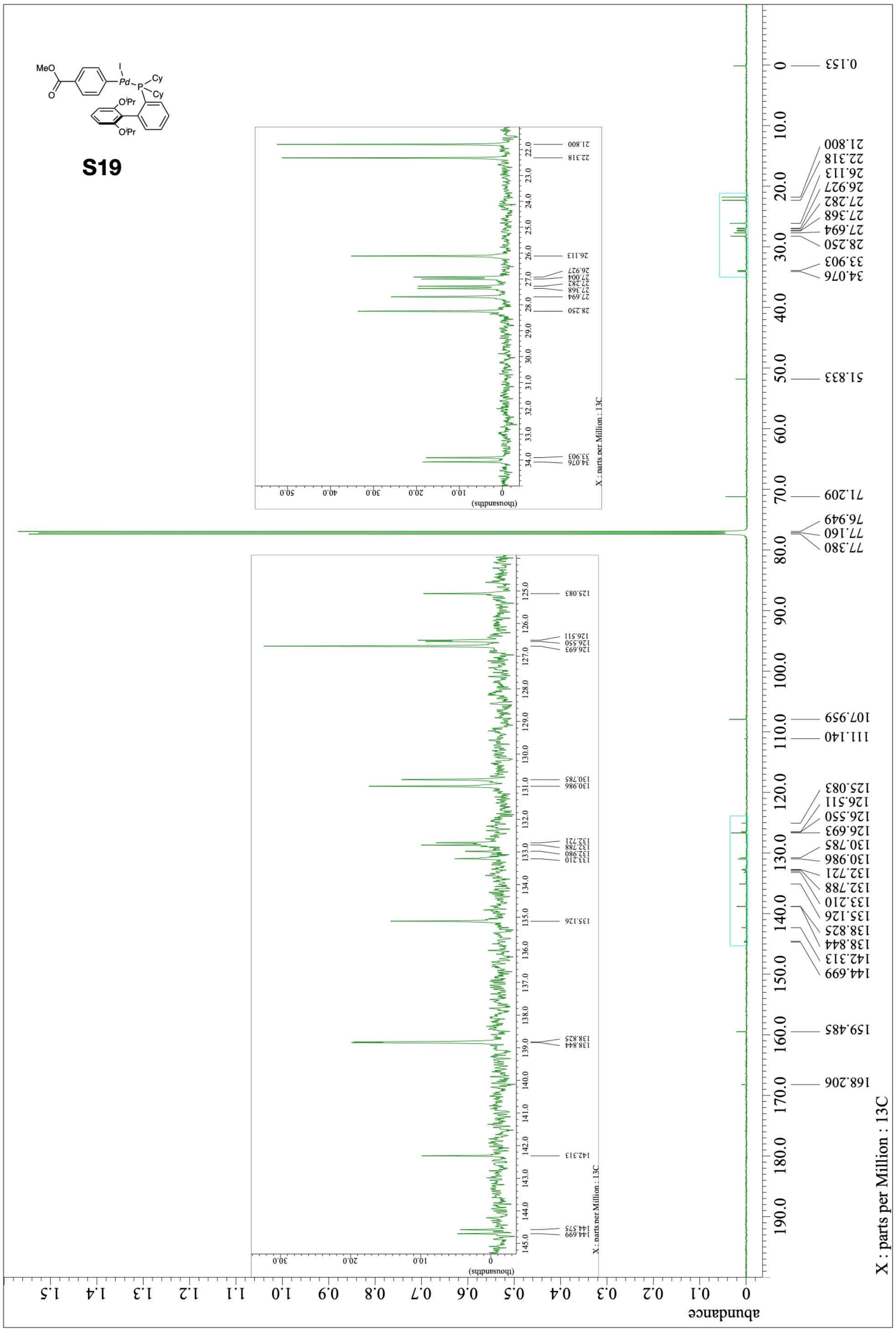

Supplementary Fig. 51. ^{13}C NMR spectrum of **S19** ($CDCl_3$, 150 MHz).

¹H NMR

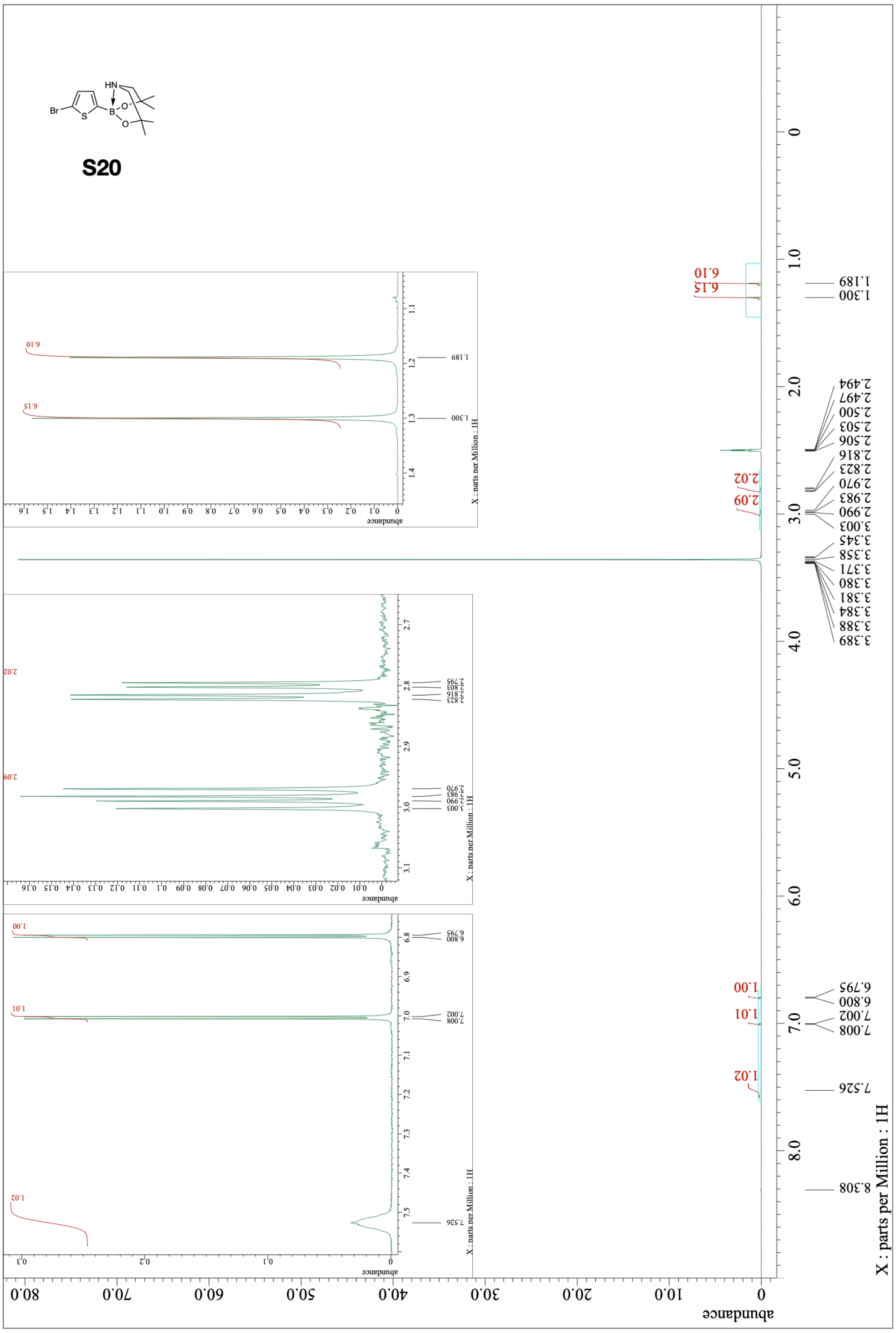

Supplementary Fig. 52. ^{13}C NMR spectrum of **S20** (DMSO-d_6, 600 MHz).

13C NMR

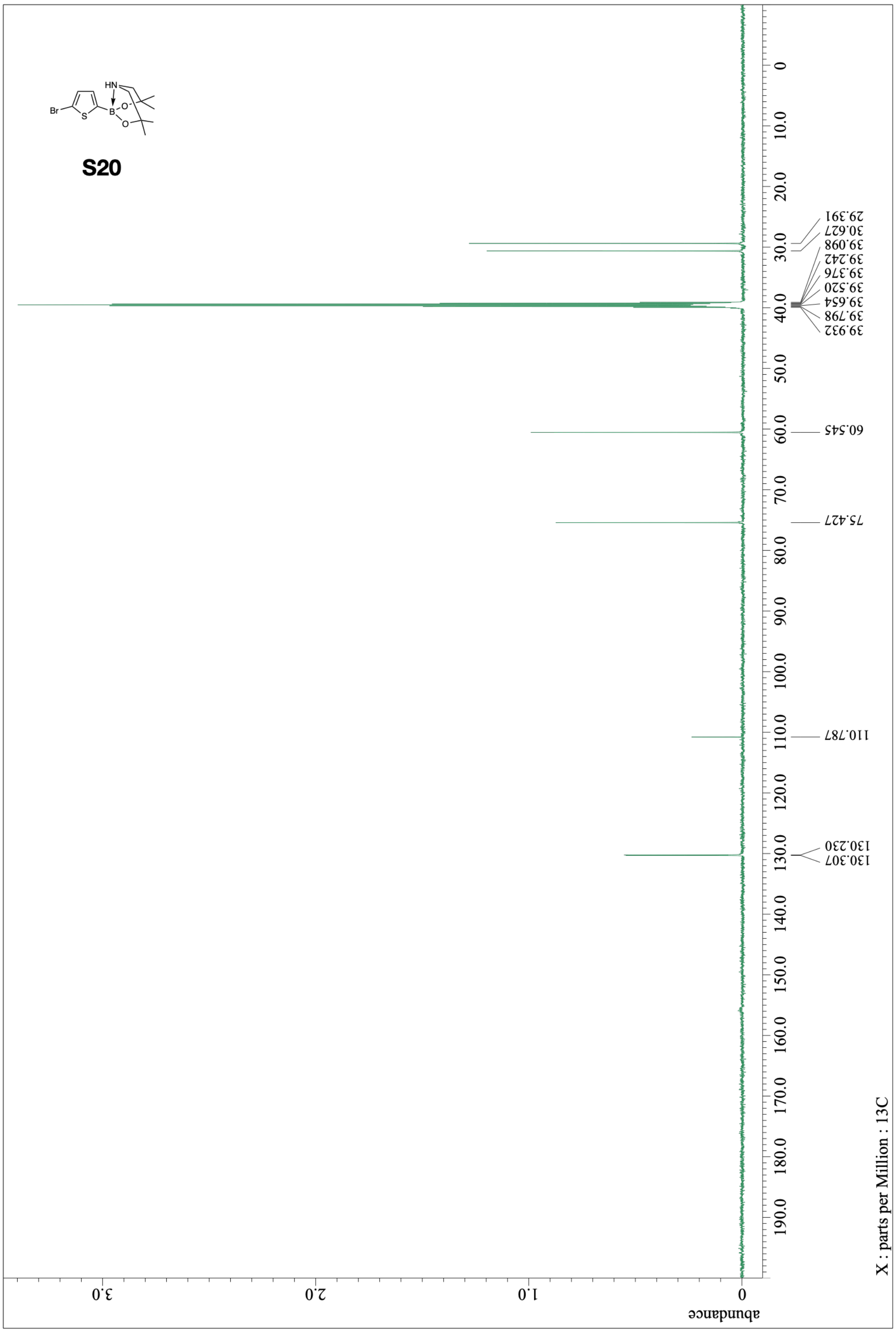

Supplementary Fig. 53. ^{13}C NMR spectrum of **S20** (DMSO-d_6, 150 MHz).
